# A role for Myo-II *zipper* and *spaghetti squash* in *Gliotactin*-dependent *Drosophila melanogaster* wing hair planar cell polarity

**DOI:** 10.1371/journal.pone.0328970

**Published:** 2025-07-23

**Authors:** Chase Holland, Natalya Cox, Hanna Dery, Mariah Williams, Gwendolyn Jones, Lindi Newton, Kristin Jurgensen, Han Chang, Alex Hord, Won Chang, Damon Montgomery, Hawkins Hochstedler, Amish Mishra, Jessica Baker

**Affiliations:** 1 Department of Biology, Taylor University, Upland, Indiana, United States of America; 2 Department of Mathematics, Taylor University, Upland, Indiana, United States of America; Pontificia Universidad Catolica de Chile, CHILE

## Abstract

Planar cell polarity, polarization in the plane of an epithelium, is critical for tissue development. The *Drosophila melanogaster* wing epithelium is an important model system for planar cell polarity establishment and has greatly informed studies in vertebrates. The well-studied Frizzled-dependent and Fat – Dachsous – Four-jointed pathways establish proximal-to-distal polarity of wing hairs, while a Frizzled-independent mechanism mediated by septate junction proteins Gliotactin, Coracle, and Varicose is required for parallel alignment of neighboring hairs. In this study, we explore a requirement for the non-muscle myosin II proteins Spaghetti Squash and Zipper in wing hair planar cell polarity. We confirm a previously recognized role in hair initiation and demonstrate a second, novel *Gliotactin*-interacting requirement for *spaghetti squash* and *zipper* in parallel alignment. Immunolabeling experiments demonstrate that Spaghetti Squash and Zipper localize to the base of the developing hairs during the same time frame that septate junction proteins transiently relocalize to the apical cell surface. This localization is abrogated in *Gliotactin* loss-of-function genotypes. We propose that Gliotactin promotes Spaghetti Squash and Zipper accumulation at the cell apical surface during wing hair extension and that this apical Myosin-II complex stabilizes the developing hair base, maintaining parallel alignment of neighboring wing hairs.

## Introduction

Polarity is a fundamental and essential characteristic of epithelial tissues and organs. Epithelial cells are polarized in the apical-basal axis through the action of cell-cell junctions and associated proteins. Epithelial sheets also polarize in the plane of the epithelium, a form of polarity known as tissue or planar cell polarity (PCP). For example, in vertebrates, PCP directs stereocilia bundle formation in the mammalian inner ear, extension of kidney tubules during embryogenesis, and orientation of cilia in ependymal cells [1–7].

The first PCP genes were identified in wing epithelium of *Drosophila melanogaster*, an important model system for both apical-basal and PCP pathways that has greatly informed studies in vertebrate PCP [8–10]. In the epithelial bilayer of the developing pupal wing, a single actin-rich hair extends from the apical surface near the distal vertex of each cell [11,12]. These hairs point distally and align in parallel with hairs from adjacent cells. Two separate, but intersecting, pathways, the Frizzled-dependent and Fat – Dachsous – Four-jointed pathways, are required for the proximal-to-distal polarity of the wing hairs [10,13]. Interestingly, mutants in both of these pathways are not characterized by a complete loss of wing hair organization [12,14,15]; rather, although distal orientation of the hairs is disrupted, individual hairs retain a parallel alignment relative to their immediate neighbors. This parallel alignment has been linked to a Frizzled-independent mechanism involving the septate junction proteins Gliotactin (Gli), Coracle (Cora), and Varicose (Vari) [16,17]. Wing hairs of *Gli* or *cora* mutants and *vari* knockdowns retain an overall distal orientation, but exhibit a parallel misalignment phenotype marked by neighboring hairs tilting towards one another [16,17].

Although mature wing hairs are most easily observed on the adult wing, development of these hairs occurs during pupation. At approximately 30 hours after puparium formation (APF), the actin-rich hairs begin extending from the distal vertices of apical cell surfaces [18–20]. By this time, Frizzled pathway components have become restricted to the distal (e.g., Frizzled, Dishevelled, and Diego), proximal (e.g., Prickle and Van Gogh/Stabismus), or proximal and distal (e.g., Flamingo/Starry night) cell boundaries and are not present on the posterior or anterior boundaries [20–25]. This asymmetry restricts the site of hair initiation in order to direct hair polarity and to limit the number of hairs per cell; *frizzled* and related mutants are characterized by hairs extending from the center of each cell surface and by multiple hairs extending from a single cell [12]. Following initiation, the wing hairs continue to extend until approximately 47 hours APF [18].

In contrast to Frizzled’s early asymmetrical localization, Gli and Cora remain symmetrically localized during early hair development; in 30 hours APF wings, Cora localizes around the entire lateral cell boundary and Gli is present at the tricellular corners [17]. Beginning around 35–36 hours APF, Gli and Cora relocalize from their lateral positions and accumulate in apical ribbons that run along the proximal-distal wing axis [17]. This relocalization is transient, and Gli and Cora lose their apical ribbon position by approximately 48 hours APF [17]. *Gli* mutant data correlating loss of ribboning with hair misalignment suggests that this relocalization is key to maintaining stability and parallel alignment of wing hairs. However, the lack of physical overlap between the Gli and Cora ribbons and the base of the developing hair suggests that this effect is indirect [17].

Given the prominence of actin in the structure of elongating hairs [11,26], actin binding proteins are intriguing candidates to mediate parallel hair alignment downstream of Gli [12,17]. However, although numerous studies have connected actin and related proteins to hair initiation and Frizzled-dependent processes [11,18,26–30], to our knowledge no studies have implicated actin related proteins in the later acting Gli-dependent hair alignment.

Here we explore the role of non-muscle myosin II in Gli-mediated PCP. Non-muscle myosin II (Myo-II) is a multimeric motor protein, physically binding to and crosslinking actin in order to regulate diverse cellular processes including adhesion, migration, cell shape change or maintenance, and mechanotransduction [31,32]. In *D. melanogaster*, the Myo-II heavy chain is encoded by *zipper* (*zip*) and the regulatory light chain is encoded by *spaghetti squash* (*sqh*; [33,34]). Both Zip and Sqh are linked to Frizzled-directed hair initiation, with *zip* and *sqh* mutant alleles genetically interacting with *frizzled* and *dishevelled* in multiple wing hair assays [29] and expression of *zip* truncation alleles leading to several wing phenotypes, including multiple wing hairs [27]. In this study, we confirm Sqh and Zip’s role in hair initiation and provide immunohistochemical and genetic evidence of a second, later requirement in Gli-dependent parallel hair alignment.

## Results

### Myo-II Zip and Sqh are required for multiple aspects of wing hair development

**Fig 1:**
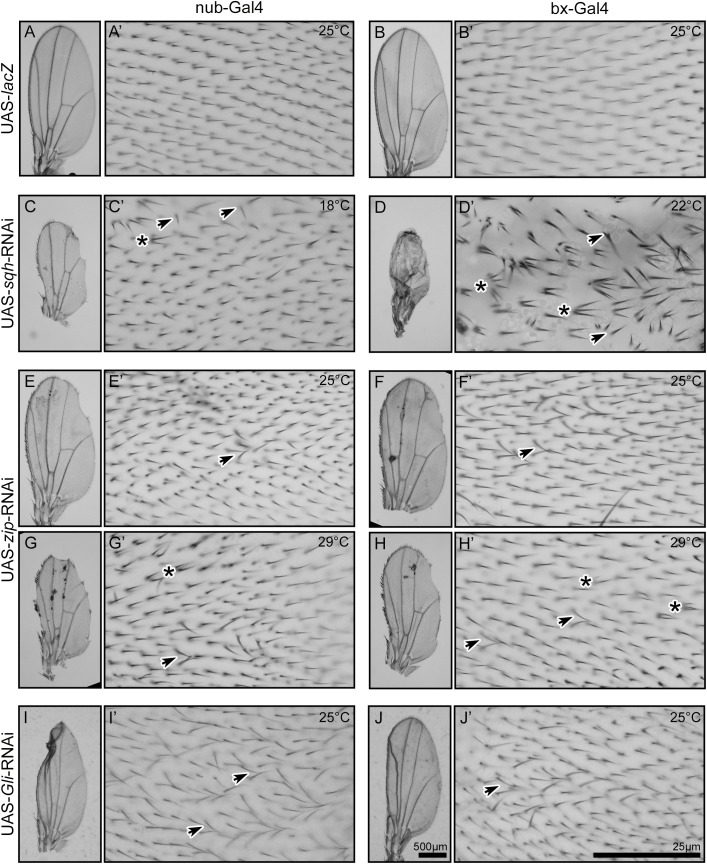
Myo-II *zip* and *sqh* are required for multiple aspects of wing hair development. Control (nub > lacZ or bx > lacZ) wings are flat and have smooth and consistent edges (A and B). Wing hairs have consistent proximal-to-distal directionality and neighboring hairs are aligned in parallel (A’ and B’). Wings with reduced Sqh or Zip or Gli were generated using wing-targeted RNAi. In contrast to wildtype wings, wings from flies with RNAi directed against either *sqh* or *zip* have an overall crumpled structure (e.g., D), serrated edges (e.g., C, E-H), and/or dark accumulations along the veins (e.g., F-H). RNAi wings also have patches of multiple wing hairs (asterisks in C’-H’) and wing hair misalignment (arrows in C’-H’). For both *sqh* and *zip*, the mutant phenotypes were more severe at higher temperatures (18°C, 22°C, 25°C, and 29°C were tested, with representative images shown). Wings from flies with RNAi directed against *Gli* were frequently crumpled, especially at the posterior distal wing edge (e.g., I). Patches of hairs had wing hair misalignment (see arrows in I’ and J’). Multiple wing hairs were not observed in *Gli*-RNAi wings. Calibration: 500 µm in A-J, 25 µm in A’-J’.

In *D. melanogaster*, the wing develops during pupation, with the wing hairs extending distally from the apical surface of each cell. The Myo-II proteins Zip and Sqh have a previously documented role early in hair initiation such that perturbing their function results in multiple hairs extending from a single cell [27,29]. However, a later role in establishing parallel alignment of the extending wing hairs has not been explored. To test this possibility, we examined the wings of *sqh* and *zip* knockdown adult flies. RNA interference (RNAi) was directed using the wing specific nub-Gal4 and bx-Gal4 drivers [35–39]. The wings of control flies (bx-Gal4 > UAS-*lacZ* and nub-Gal4 > UAS-*lacZ*) were flat and had smooth and consistent edges (Fig 1A, 1B). Wing hairs had consistent proximal-to-distal directionality and neighboring hairs were aligned in parallel (Fig 1A’, 1B’). Flies expressing *sqh*-RNAi or *zip*-RNAi exhibited a range of phenotypes affecting viability, wing morphology, and wing hairs. Despite bx-Gal4 and nub-Gal4 primarily directing expression in developing wing tissue, several RNAi lines resulted in pupal or earlier lethality (RRID:BDSC_36727, RRID:BDSC_65947, RRID:BDSC_31542, RRID:BDSC_32439). Knockdowns of *zip* and *sqh* using RRID:BDSC_37480 and RRID:BDSC_33892, respectively, resulted in surviving adult flies and were used for all further analysis. Besides the phenotype severity being higher in *sqh*-RNAi wings, no phenotypic differences were noted between *sqh*-RNAi and *zip*-RNAi wings. Penetrant wing morphology phenotypes of adult survivors included serrated wing edges (e.g., Fig 1C, E–H), branched veins (e.g., distal end of Fig 1F, H; [27]), spots of dark tissue along the veins (e.g., Fig 1F-H), and partially or fully crumpled wings (e.g., Fig 1D). These phenotypes are notable but are not directly related to hair development, so they were not investigated further in this study.

Imaging of the wing hairs revealed a multiple wing hair phenotype (Fig 1C’-1H’), consistent with Sqh and Zip’s previously documented roles in hair initiation [27,29]. Intriguingly, in addition to the anticipated multiple wing hair phenotype, some patches of the wing also exhibited a hair misalignment phenotype. Despite retaining an overall proximal-to-distal orientation, hairs were not always aligned in parallel with adjacent hairs but angled or tilted towards their neighbor (Fig 1C’-1H’ and Table 1). Notably, although some misalignment may have been secondarily caused by wing folding or multiple hairs splaying out from one another (e.g., Fig 1D’), this phenotype was also observed in flat regions of the wing devoid of multiple wing hairs. The misalignment phenotype is reminiscent of that observed in wings with loss-of-function or reduced expression of septate junction factors *Gli*, *cora*, or *vari* [16,17]. Indeed, bx-Gal4 and nub-Gal4 targeted RNAi knockdown of *Gli* resulted in frequent patches of misaligned hairs (Fig 1I-J). Notably, multiple wing hairs were not observed in *Gli* knockdown wings. Taken together, the data suggests that Sqh and Zip are required for multiple aspects of wing hair development, including hair initiation and parallel alignment of neighboring wing hairs.

To test if the misalignment phenotype was specific to the Myo-II proteins Sqh and Zip, we tested the requirement of other myosins in wing hair development. Bx-Gal4 driven RNAi targeting *dachs*, *diddum*, *Myosin heavy chain (Mhc)*, *Myosin heavy chain-like (Mhcl)*, *Myosin28B1*, *Myosin31DF* (*Myo31DF*), *Myosin61F* (*Myo61F*), and *Myosin95E* (*Myo95E*) did not reveal any consistent hair phenotypes, although there were occasional multiple wing hairs present in *dachs*, *Mhcl*, *Myo31DF*, and *Myo95E* knockdown flies (S1A-I Fig). Consistent with previously published studies of myosin VIIA *crinkled* (*ck*) loss-of-function alleles [26,29,40], RNAi knockdown of *ck* resulted in a multiple hair phenotype with 2–6 short hairs extending from each cell (S1J-J’ Fig). As a group, the hairs from each cell generally pointed in a proximal-to-distal direction, however individual hairs within each group flared out from one another in a “starburst” pattern. No obvious examples of hair misalignment were observed; however, the starburst pattern limited the analysis of a potential phenotype. Knockdown of the myosin VI *jaguar* (*jar*) yielded a multiple wing hair phenotype of 2–3 hairs per cell (S1K-K’ Fig). In contrast to *ck* RNAi flies, the hairs in the *jar* knockdown wings were long (similar to controls) and hairs originating from a single cell were parallel to one another (no starburst effect). In some regions of the wing, individual hairs had a slight curve as the hair tapered. Although the proximal-to-distal orientation of the hairs was not as consistent as observed in controls, individual hairs remained parallel to their immediate neighbors. Bx-Gal4 driven knockdown of *Myosin10A* (*Myo10A*) resulted in regions of wings with multiple hairs and some loss of proximal-to-distal orientation (S1L-L’ Fig), reminiscent of *frizzled*-pathway mutants. Loss of parallel alignment was not observed. Taken together, RNAi mediated knockdown of myosin genes yielded a range of intriguing wing hair phenotypes. However, other than *sqh*-RNAi and *zip*-RNAi, no RNAi flies phenocopied the *Gli* hair misalignment phenotype characterized by hairs tilting towards their immediate neighbors.

### *sqh* and *zip* genetically interact with *Gli* during wing hair development

**Fig 2:**
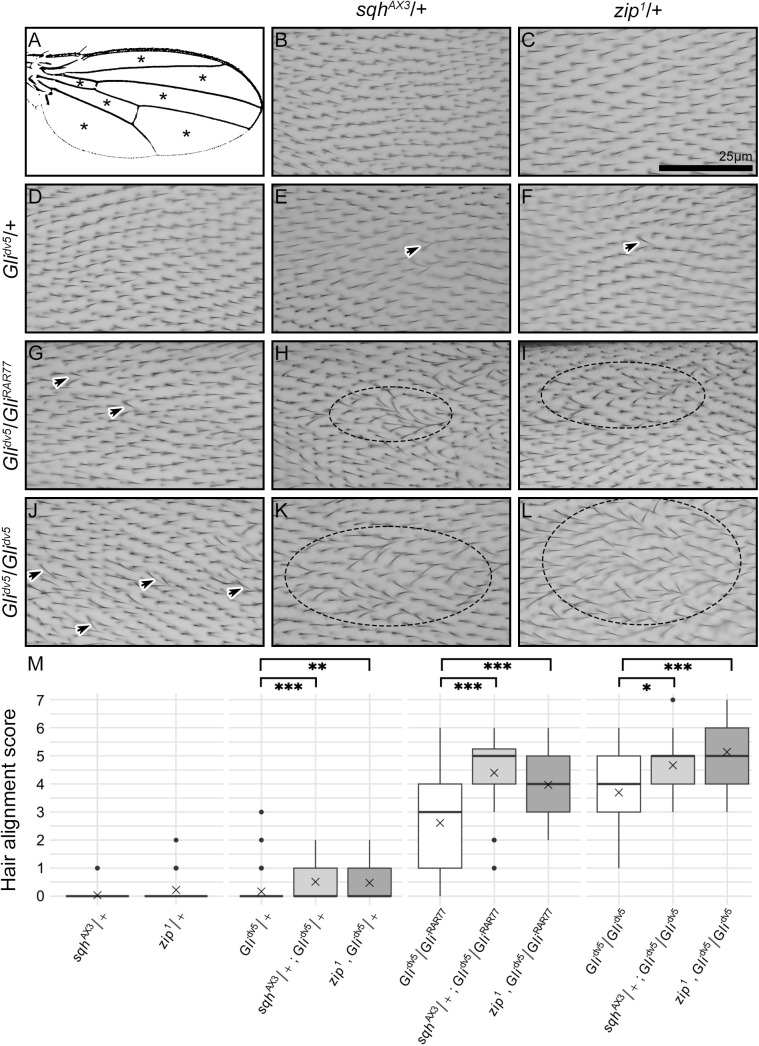
*zip*^*1*^ and *sqh*^*AX3*^ dominantly enhance *Gli* mutant wing hair misalignment. (A) *D. melanogaster* wing veins separate the wing into seven regions (indicated by asterisks). (B-L) Wing hairs from heterozygotes for *sqh*^*AX3*^ (B), *zip*^*1*^ (C), and *Gli*^*dv5*^ (D) have consistent proximal-to-distal directionality and parallel alignment of neighboring hairs. Flies doubly heterozygous for *Gli*^*dv5*^ and either *sqh*^*AX3*^ or *zip*^*1*^ occasionally have neighboring hairs that tilt towards one another (E-F, arrows). *Gli*^*dv5*^/*Gli*^*RAR77*^ (G) and *Gli*^*dv5*^/*Gli*^*dv5*^ (J) mutant wings exhibit neighboring hairs that tilt towards one another. *Gli* mutants that additionally are heterozygous for either *sqh*^*AX3*^ (H, K) or *zip*^*1*^ (I, L) have increased hair misalignment, often in patches (dotted ellipses in H, I, K, L). B-L calibration: 25 µm. (M) Misalignment severity was quantified by counting the number of regions per wing with misaligned hairs. For box plots, center lines show the median values, box limits indicate the 25^th^ and 75^th^ percentiles, whiskers extend 1.5 times the interquartile range (IQR) from the 25^th^ and 75^th^ percentiles, and dots indicate outliers beyond the 1.5 IQR. X indicates the mean value. Kruskal-Wallis and Dunn’s tests were used to identify *sqh*^*AX3*^/+ and *zip*^*1*^/ + enhanced genotypes that significantly differed from the *Gli* mutant base (comparisons indicated by the brackets). * p = 0.017, ** p = 0.005, *** p < 0.001.

The similarity of the hair misalignment phenotype in *sqh*, *zip*, and *Gli* knockdown wings suggests that *sqh* and *zip* may genetically interact with *Gli* to regulate hair alignment. If so, we reasoned that reducing the gene dosage of *sqh* or *zip* in a *Gli* mutant background would enhance the *Gli* wing hair phenotype. To test this, we quantified the misalignment phenotype by dividing the wing into seven regions using the veins as boundaries (Fig 2A) and counting the number of regions that displayed patches of misaligned hairs (as per [17]). The number of regions with misaligned hairs was termed the hair alignment score.

Heterozygotes for either *Gli*^*dv5*^, *sqh*^*AX3*^, or *zip*^*1*^ displayed a wildtype wing hair parallel alignment phenotype (compare Fig 2B-D to Fig 1A), with median wing scores of 0 and mean scores of 0.17, 0.03, and 0.22 respectively (Fig 2M). Wings that were doubly heterozygous for *Gli*^*dv5*^ and either *sqh*^*AX3*^ or *zip*^*1*^ frequently displayed parallel alignment with a median hair alignment score of 0; however, there were significantly greater instances of minor misalignments when compared to *Gli*^*dv5*^/ + , with mean wing scores of 0.51 and 0.48 respectively (Fig 2E, F, M). This minor, but statistically significant, dominant enhancement of the *Gli*^*dv5*^/ + hair alignment phenotype is consistent with *sqh* and *zip* genetically interacting with *Gli*.

**Table 1:** Quantification of *sqh*-RNAi and *zip*-RNAi wing hair misalignment.

	*bx*-Gal4				*nub*-Gal4		
	*lacZ*	*sqh*-RNAi	*zip*-RNAi		*lacZ*	*sqh*-RNAi	*zip*-RNAi
18°C	**0** (1.3)	14*** (16.1)	4* (5.4)	18°C	**0** (1.1)	4*** (6.5)	**0** (2.3)
22°C	**0** (0.7)	16*** (16.1)	n.d.	22°C	**0** (0.4)	8*** (8.1)	n.d.
25°C	**0** (0.8)	n.d.	4*** (5.3)	25°C	**0** (0.4)	n.d.	4*** (4.2)
29°C	**0** (0.6)	n.d.	8*** (8.2)	29°C	**0** (0.8)	n.d.	4*** (5.9)

Median (bold) and mean (in parentheses) percentages of misaligned wing hairs in indicated genotypes. Scored regions were selected from flat portions of each wing and encompassed 50 hairs per region. Forty wings per genotype were sampled. Due to severe wing folding, *sqh*-RNAi genotypes raised at 25°C and 29°C could not be scored. *p = 0.002, *** < 0.001, relative to temperature-matched *lacZ* control (Mann-Whitney U for 22°C, 25°C, and 29°C single pair analyses or Kruskal-Wallis followed by Dunn’s tests for 18°C multiple pair analyses).

Given the subtle differences when assessing single and double heterozygotes, we sought to further test for genetic interactions using stronger *Gli* mutants. Null mutants of *Gli* are lethal [41], however viable *Gli* hypomorphs exhibit moderate hair misalignment [17]. *Gli*^*dv5*^/*Gli*^*RAR77*^ wings had a median score of 3 (mean of 2.61) and *Gli*^*dv5*^/*Gli*^*dv5*^ wings had a median score of 4 (mean of 3.70; Fig 2G, J, M). Reducing the gene dosage of either *zip* or *sqh* in these *Gli* sensitized backgrounds dominantly enhanced the wing hair misalignment phenotype. Compared to the *Gli*^*dv5*^/*Gli*^*RAR77*^ and *Gli*^*dv5*^/*Gli*^*dv5*^ flies which retained full *zip* and *sqh* gene dosage, in *Gli*^*dv5*^/*Gli*^*RAR77*^ and *Gli*^*dv5*^/*Gli*^*dv5*^ flies also heterozygous for either *zip*^*1*^ or *sqh*^*AX3*^, the patches of misaligned hairs were qualitatively larger (compare Fig 2H, I, K, and L to Fig 2G and J) and significantly more regions of the wings displayed misaligned patches (Fig 2M). Specifically, *sqh*^*AX3*^/ + enhanced both the *Gli*^*dv5*^/*Gli*^*RAR77*^ and *Gli*^*dv5*^/*Gli*^*dv5*^ median scores to 5 (means of 4.40 and 4.67, respectively), and *zip*^*1*^/ + enhanced the *Gli*^*dv5*^/*Gli*^*RAR77*^ and *Gli*^*dv5*^/*Gli*^*dv5*^ scores to 4 and 5 respectively (means = 3.97 and 5.14).

To directly test if these enhancements were synergistic, i.e., more than additive, we compared the wing scores of *Gli* hypomorphs and of *sqh*^*AX3*^ or *zip*^*1*^ heterozygotes with those of flies simultaneously carrying both the *Gli* hypomorph and the *sqh*^*AX3*^ or *zip*^*1*^ alleles (S5 File). Wings of genotypes *sqh*^*AX3*^/ + ; *Gli*^*dv5*^/*Gli*^*RAR77*^ and *Gli*^*dv5*^*, zip*^*1*^*/Gli*^*RAR77*^ displayed alignment defects that were significantly greater than the additive effects of *sqh*^*AX3*^/+ and *Gli*^*dv5*^/*Gli*^*RAR77*^ *or zip*^*1*^/+ and *Gli*^*dv5*^/*Gli*^*RAR77*^ alone (p < 0.001 and p = 0.025, respectively). Similarly, wings of genotypes *sqh*^*AX3*^/ + ; *Gli*^*dv5*^/*Gli*^*dv5*^ and *Gli*^*dv5*^*, zip*^*1*^/*Gli*^*dv5*^ displayed alignment defects that are significantly greater than the additive effects of *sqh*^*AX3*^/+ and *Gli*^*dv5*^/*Gli*^*dv5*^ *or zip*^*1*^/+ and *Gli*^*dv5*^/*Gli*^*dv5*^ alone (p = 0.014 and p = 0.021, respectively). In contrast, although *sqh*^*AX3*^ and *zip*^*1*^ heterozygotes enhanced the wing hair phenotype of *Gli*^*dv5*^ heterozygotes (as discussed above), hair alignment score analysis did not reveal that this enhancement was synergistic.

In short, these experiments demonstrate that both *sqh*^*AX3*^ and *zip*^*1*^ dominantly enhance all tested *Gli* mutant genotypes and these enhancements are significantly more than additive for hypomorphic *Gli* allele combinations. Together, these experiments suggest that Sqh and Zip function through the Gli-mediated pathway to modulate wing hair parallel alignment.

### Sqh and Zip localize to the wing hair base during hair extension

During wing hair development in pupae, wing cells shift the localization patterns of PCP determinants. By 30 hours APF, when hair development initiates, Frizzled pathway components are asymmetrically localized on the proximal and/or distal cell boundaries [20–25]. In contrast, at 30 hours APF, the septate junction protein Cora is located around the entire cell circumference and Gli is present at the tricellular corners [11,17]. Similarly, we found that Discs large (Dlg) – a septate junction resident protein [42] – also localized around the cell periphery at 30 hours APF (S2 Fig). Gli, Cora, and Dlg started to accumulate apically in a “ribbon” pattern beginning around 35–36 hours APF and then more sharply by 38 hours (S2 Fig; [11,17]), with low levels of Cora and Dlg remaining around the cell circumference basolaterally. These apical ribbons run along the proximal-distal axis, perpendicular to the Frizzled pattern observed hours earlier. This relocalization is transient; by 46–47 hours APF, the proximal-distal apical ribbons were no longer present and Gli, Cora, and Dlg were restricted to their tricellular or lateral junctional positions (S2 Fig; [17]). Notably, the *Gli* and *cora* mutant hair misalignment phenotype arises after 38 hours APF, with the first examples of misalignment visible by 47 hours APF [17].

**Fig 3:**
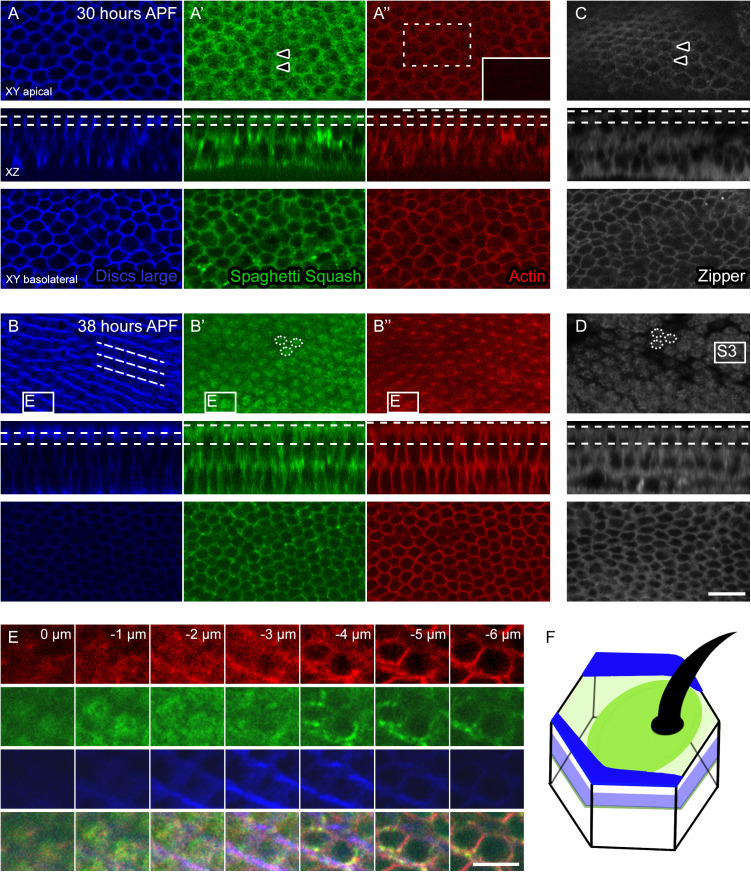
Sqh and Zip accumulate at the apical cell surface during pre-hair extension. (A-D) Apical (XY apical), mid-cell (XY basolateral), and transverse (XZ) images of wildtype pupal wings at 30 and 38 hours APF. Dotted lines in the XZ image indicate the positions of the apical and basolateral XY planes. The inset in A” displays a plane immediately above the apical cell surface, at the position of the developing hairs (inset was imaged at the position indicated by the dashed rectangle). (E) Enlarged images of the boxed region in B-B” shown at descending 1 µm Z intervals, with the 0 µm position set at the level of the extended hairs above the apical cell surface. See S3A Fig for enlarged images of boxed region in D. (F) A cartoon summary of the Dlg (blue) and Sqh and Zip (green) localization at 38 hours APF. (A-F) In wildtype pupal wings at 30 hours APF, Actin (Phalloidin, red) and Dlg (blue) surround the cell periphery at both the apical and basolateral positions (A, A”). No extended hairs are present (A,” inset). Sqh-GFP (green, A’) and Zip-GFP (grey, C) surround the cell periphery, with diffuse patterns visible along the apical cell surface, with slight concentrations at the distal vertex (arrowheads). By 38 hours APF, the developing hairs (B,” E 0 µm) extend above the apical surface of each cell and Dlg (B, E −2 to −4 µm) forms ribbons below the apical surface of the wing (dotted parallel lines track three example ribbons). Unlike the diffuse pattern at 30 hours APF, Sqh-GFP (green, B’, E −1 µm) and Zip-GFP (grey, D) are present in circular accumulations near the cell surface (dotted ellipses outline example accumulations). Note that in A-A,” the apical planes were all imaged at the same Z position, while in B-B” the apical planes differed. This reflects a shift in the most apical position at which the relevant label was detected, which can be more clearly seen in E. At both 30 and 38 hours APF, Actin, Dlg, Sqh-GFP, and Zip-GFP are present around the cell periphery basolaterally (A-D bottom panels, E −5 to −6 µm). For both 30 and 38 hours, Actin, Dlg, and Sqh-GFP were imaged in a single wing, while Zip-GFP is shown in a different wing (also labeled with Dlg, as shown in S3A Fig). The bottom row in E indicates the Actin (red), Sqh-GFP (green), and Dlg (blue) merge. Calibration: 10 µm in A-D, 5 µm in E.

We speculated that the Sqh and Zip localization pattern and timing may reflect their role in maintaining parallel alignment. In particular, given that actin is a key structural component of developing hairs [18,26] and that non-muscle myosin IIs are able to bind to and crosslink actin, we asked whether Zip and Sqh localize near the developing hair during hair extension, when the septate junction proteins form apical ribbons. To determine if Sqh and Zip localization correlated with septate junction patterns, we assessed their position at 30 and 38 hours APF using Sqh and Zip GFP fusion proteins (RRID:BDSC_57145 and RRID:BDSC_51564, respectively) [43,44]. Given the similarity of Dlg to Cora and Gli localization patterns, we utilized Dlg as a septate junction marker here and throughout the remainder of this study. Wings were also labeled with Phalloidin to visualize F-actin. At 30 hours APF, actin and Dlg were present around the cell periphery near both the apical cell surface and basolaterally (Fig 3A, A”). Consistent with this being the approximate time of hair initiation, no hair extensions above the apical plane were observed (inset in Fig 3A”). Also at 30 hours APF, Sqh-GFP and Zip-GFP were detected around the cell periphery along with diffuse patterns visible along the apical cell surface, especially near the distal vertex (Fig 3A’,C, top panels). At 38 hours APF, similar to that observed at 30 hours, actin, Dlg, Sqh-GFP, and Zip-GFP were all detected around the cell periphery below the apical cell surface (Fig 3B, D, bottom panels). In contrast, localization patterns at the apical cell surface differed from those observed at 30 hours APF. By 38 hours, the actin rich hairs extended above the apical surface of each cell (Fig 3B,” top panel). Close to the apical surface, but below the hairs, Dlg localized in proximal-distal ribbons (Fig 3B, top panel). Unlike their diffuse pattern along the apical surface at 30 hours APF, by 38 hours both Sqh-GFP and Zip-GFP were detected in circular accumulations at the surface of each cell (Fig 3B’ and 3D, top panels). In the XZ plane, these accumulations were positioned immediately below the base of the extending wing hairs and apical to the Dlg ribbons (compare Z position of top panels in Fig 3B-B”).

To more closely compare the myosin and septate junction XY and XZ positions to one another, we assessed the patterns of Sqh-GFP, Dlg, and F-actin at one micron Z-axis intervals (Fig 3E). At the most apical position (indicated as the 0 µm position), the extended hairs could be detected as faint projections above the cell surface. Minimal Dlg and Sqh-GFP labels were detected at this position. Immediately below (−1 µm), Sqh-GFP was detected in circular accumulations, which appeared to be positioned around the base of the extending hair (compare red and green in merge images at 0 and −1 µm). By −3 µm, the circular Sqh-GFP accumulations were no longer apparent, but Dlg ribbons were visible. A close examination of the XY position of Sqh-GFP at −1 µm and the Dlg ribbons at −2 to −4 µm suggests that the Dlg ribbons run along the cell boundaries, anterior and posterior to the Sqh accumulations. Thus, in both the XY and XZ planes, Dlg and Sqh-GFP are in close proximity to one another, but do not appear to co-localize near the apical cell surface. We observed similar patterns with Dlg and Zip-GFP (S3A Fig). Further below the cell surface, shown here at −5 to −6 µm, actin, Sqh-GFP, Zip-GFP, and Dlg localize around the cell periphery (Fig 3E, S3A Fig).

Taken together, our data demonstrate that Sqh and Zip’s apical localization patterns shift between 30 and 38 hours APF. By 38 hours APF, Sqh and Zip localize in apical accumulations around the base of the developing hair. The timing of this accumulation correlates with the formation of Dlg-marked septate junction ribbons along the proximal-distal wing axis. Although Sqh and Zip are in close proximity to Dlg, they do not appear to co-localize with Dlg near the developing hairs.

### Gli is required for Sqh and Zip apical accumulation

**Fig 4:**
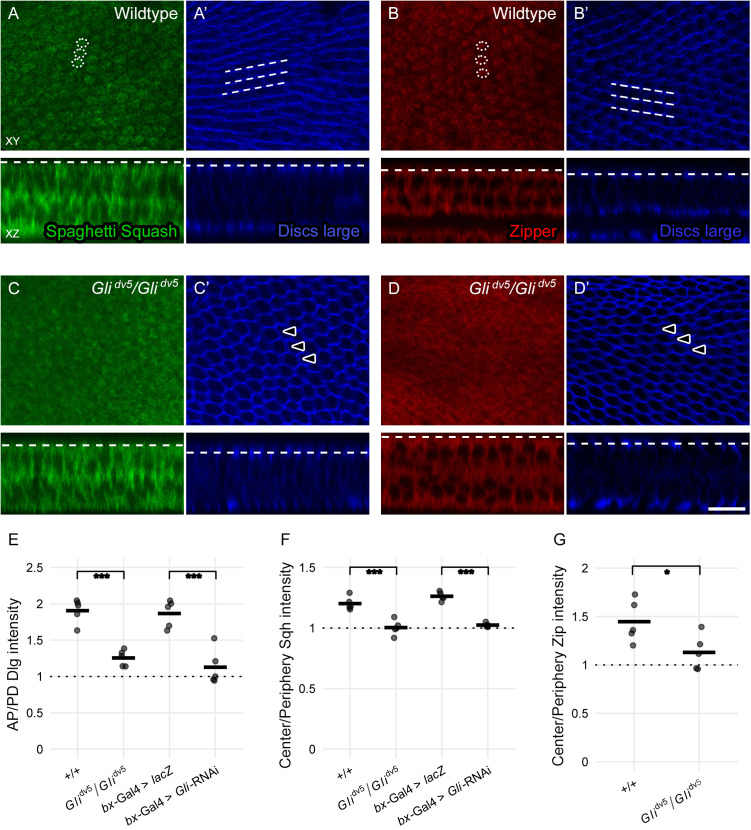
*Gli* is required for Sqh and Zip apical accumulation. (A-D) Apical (XY) and transverse (XZ) sections of pupal wings at 38 hours APF. The dotted lines in the XZ images indicate the positions of the XY planes. In wildtype wings, Dlg (blue) accumulates in apical ribbons at 38 hours APF, with minimal to no Dlg present at proximodistal cell boundaries (A’, B’; dotted parallel lines track three example ribbons). Immediately apical to these ribbons, Sqh-GFP (green) and Zip-GFP (red) are present in circular accumulations (A and B; dotted ellipses outline example accumulations). In *Gli*^*dv5*^*/Gli*^*dv5*^, Dlg does not form continuous ribbons, but remains around the lateral cell surface (e.g., C’, D’). Arrowheads indicate residual accumulation at the proximodistal cell boundaries. Unlike in wildtype, Zip and Sqh do not accumulate apically in *Gli*^*dv5*^*/Gli*^*dv5*^, but remain diffuse at the apical surface (C, D). Calibration: 10 µm. (E-G) Quantification of Dlg, Sqh, and Zip apical localization. Dlg localization in proximal-distal ribbons was quantified as the ratio of fluorescence intensity along anteroposterior (AP) cell boundaries relative to proximodistal (PD) cell boundaries. Sqh-GFP and Zip-GFP localization in apical circular accumulations was quantified as a ratio of fluorescence intensity within a circular patch around the apical cell center (CC) relative to the apical cell periphery (CP). The intensity ratios were calculated for five wings per genotype (with five cells sampled and averaged per wing). For plots, dots indicate the average ratio per wing and horizontal lines show the mean values. * p = 0.038, *** p < 0.001 (Welch’s 2-sample t-test for Zip-GFP single pair analyses or ANOVA followed by a Tukey’s HSD test for Dlg and Sqh-GFP multiple pair analyses).

The close proximity of Sqh and Zip to both the Dlg-marked septate junction ribbons and the base of the developing hair led us to hypothesize that this localization is key to their Gli-interacting role in maintaining parallel hair alignment. In the *D. melanogaster* pupal notum, cells mutant for the tricellular septate junction *anakonda* had enriched Myo-II (Sqh-GFP) at both the cell junction and the medial-apical actin meshwork [45]. In contrast, in embryonic wound repair, septate junction mutants displayed reduced Myo-II (Zip-GFP) at the actomyosin cable at the wound repair edge [46]. We predicted that Gli is required for Sqh and Zip to relocalize into the circular accumulations under the developing hair base. To test this, we compared Sqh-GFP and Zip-GFP localization at 38 hours APF in both wildtype and *Gli* hypomorphic wings. In wildtype wings, Sqh-GFP and Zip-GFP accumulated in circular patches at the apical surface of the wing cells, immediately above the Dlg-marked septate junction ribbons (Fig 4A-B’). The circular accumulations were positioned around the apical cell center, with minimal Sqh-GFP and Zip-GFP around the apical cell periphery (Fig 4A-B,F-G). In contrast, in *Gli* mutant wings, Sqh-GFP, Zip-GFP, and Dlg localization patterns were disrupted. The apical pattern of both Sqh-GFP and Zip-GFP was diffuse, with less distinct accumulations present in some samples (Fig 4C-D, F-G). This diffuse pattern correlated with a loss of septate junction ribboning. In comparison to wildtype wings, Dlg remained visible at the proximodistal cell boundaries in *Gli* mutant wings, resulting in absent or incomplete proximal-distal ribbons (Fig 4C’, D’, E). This is similar to the loss of Cora and Gli ribboning in *Gli* mutants observed by Venema et al. 2004 [17]. To test if these results were limited to the *Gli*^*dv5*^/*Gli*^*dv5*^ genotype, we also reduced *Gli* expression using Bx-Gal4 driven UAS-*Gli*-RNAi (Fig 4E-F and S3B-C Fig). Similar to the *Gli*^*dv5*^/*Gli*^*dv5*^ mutant wings, knockdown of *Gli* resulted in a diffuse pattern of Sqh-GFP and loss of Dlg ribboning. These results suggest that Gli is required for apical accumulation of Sqh and Zip and are consistent with Gli working upstream of or in concert with Sqh and Zip localization or stabilization machinery.

### Knockdown of *sqh* or *zip* does not fully prevent localization of Dlg to apical ribbons

**Fig 5:**
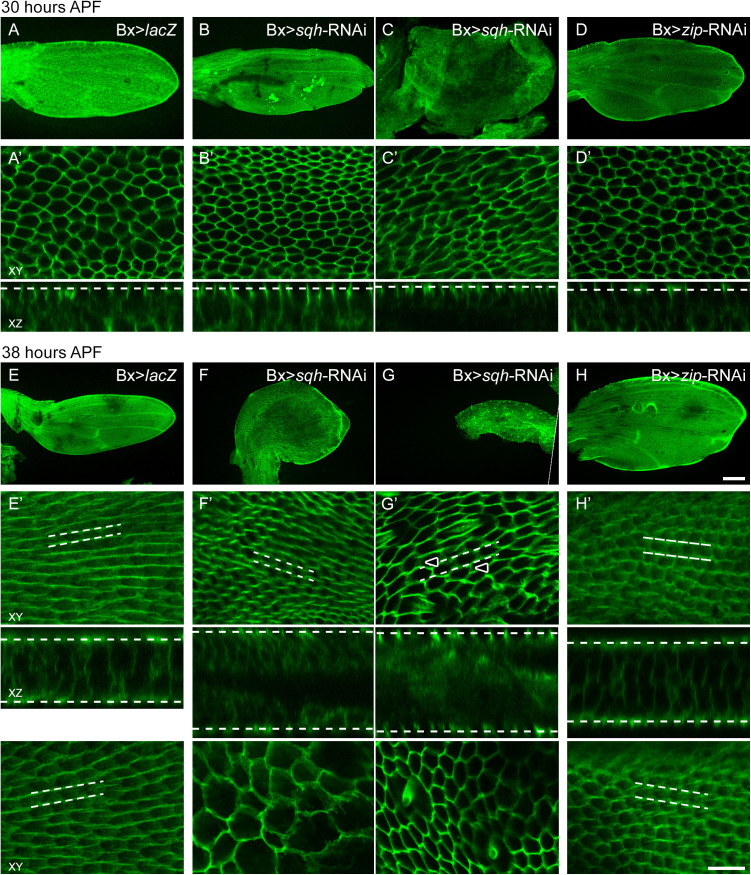
Inconsistent Dlg ribboning in wings with reduced *sqh* or *zip.* Full wing (A-H) and apical and transverse sections (XY and XZ respectively, A’-H’) of Dlg-labeled pupal wings at 30 and 38 hours APF. The dotted lines in the XZ images indicate the positions of the XY planes. At 30 hours APF, control pupal wings have a consistent arrangement of hexagonal cells with Dlg surrounding the cell peripheries (A’). By 38 hours APF, Dlg is present in apical ribbons on both the dorsal and ventral wing surfaces (E’ top and bottom panels; dotted parallel lines track two example ribbons). At both 30 and 38 hours APF, the overall wing shape is flat and regularly patterned (A and E). In contrast, wings with RNAi directed against *sqh* have disruptions in overall wing shape (B, C, F, G) and cell size (e.g., compare cell size differences in the top and bottom panels of F’). Because of the variable phenotypes, two examples of bx > *sqh*-RNAi wings at each stage are shown. Wings with reduced *zip* had normal to slightly disrupted overall wing shape (D and H). Despite these wing shape distortions, in bx > *sqh*-RNAi and bx > *zip*-RNAi wings Dlg was normally localized to the cell periphery at 30 hours APF (B’, C’). By 38 hours APF, localization patterns were varied in bx > *sqh*-RNAi wings. In some wing layers – particularly those with severe cell distortion – Dlg remained along the entire cell periphery (e.g., bottom panels in F’, G’). In other wings, apical ribboning was evident, either fully (e.g., top panel in F’) or partially (e.g., top panel in G’; arrowheads indicate examples of lower intensity of Dlg signal at proximodistal cell boundaries). In bx > *zip*-RNAi wings at 38 hours APF (H’), Dlg apical ribboning was present, but less continuous than in controls. In E’-H’, the top and bottom XY panels are apical sections of the dorsal and ventral wing surfaces taken at the same XY position. Because the Dlg localization pattern was identical in both surfaces, only a single wing surface is shown in A’-D’. Calibration: 100 µm in full wing panels, 10 µm in all others.

The disruption of apical Sqh and Zip accumulation in *Gli* mutants does not preclude the possibility that Myo-II and the septate junction are co-dependent on one another for their localization patterns during wing hair extension. Therefore, to test if *sqh* or *zip* loss-of-function disrupts formation of the septate junction ribbons, we compared Dlg in *sqh*-RNAi and *zip*-RNAi wings to Dlg in control wings. By 30 hours APF, control wings adopted a flat “paddle” shape and Dlg localized around the periphery of each cell (Fig 5A-A’). Wings with reduced *sqh* (Bx > *sqh*-RNAi) displayed a range of abnormal shapes (two examples shown in Fig 5B and 5C). Despite the resulting disruptions in cell shape, Dlg remained around the cell peripheries on both the dorsal and ventral wing surfaces in all wings imaged (Fig 5B’,C’; for simplicity, only one wing surface is shown). Wings with knockdown of *zip* (Bx > *zip*-RNAi) generally retained a normal overall wing morphology and Dlg remained localized as in the control wings (Fig 5D-D’). By 38 hours APF, Dlg formed continuous ribbons on both the dorsal and ventral wing surfaces of wildtype wings (Fig 5E’). Consistent with the wing morphology phenotypes observed at 30 hours APF, *sqh* knockdown wings were severely misshapen (Fig 5F and 5G) and individual cells had a range of sizes, with large variation observed even between surfaces of a single wing (e.g., Fig 5F’). Dlg localization was varied. In some wing layers – particularly those with severe cell distortion – Dlg remained at the cell periphery (e.g., bottom panels in Fig 5F’, 5G’). In other wing layers, Dlg ribboning was evident, either fully (Fig 5F’ top panel) or partially (Fig 5G’ top panel, note the lower intensity of Dlg signal at proximodistal cell boundaries relative to anteroposterior boundaries). In bx > *zip*-RNAi wings at 38 hours APF, wing morphology was normal or slightly misshapen and Dlg apical ribboning was present, but less continuous than in controls (Fig 5H’). Taken together, the data suggest that Sqh and Zip are not required for positioning of the septate junction at the lateral cell surface at 30 hours APF, but are required for the septate junction to fully or consistently localize into ribbons by 38 hours APF. Given that loss of ribboning correlated with severe cell morphology disruptions and that ribboning was present in wing surfaces with normal cell shape, it is likely that this requirement is an indirect effect of abnormal cell morphology.

## Discussion

The experiments we report here establish a role for Myo-II Sqh and Zip in parallel alignment of developing wing hairs of *D. melanogaster*. During hair extension, Sqh and Zip accumulate at the apical cell surface and surround the base of the developing hair. This accumulation is dependent on the septate junction protein Gli; Sqh and Zip remain diffuse in *Gli* mutants and *Gli*-RNAi knockdown wings. In *Gli*, *sqh*, and *zip* knockdown wings, patches of hairs on the adult wing display a nonparallel hair alignment phenotype. *sqh* and *zip* heterozygotes enhance a hypomorphic *Gli* phenotype, suggesting that *sqh* and *zip* genetically interact with *Gli* during hair development. Taken together, our study demonstrates that Sqh and Zip work together with or in parallel to Gli to align developing wing hairs.

### Myo-II has multiple roles in wing hair development

Studies of *D. melanogaster* wing hair development have identified dozens of genes which are often categorized based on their mutant phenotypes [3]. Mutant alleles that broaden the hair initiation site or prevent coalescence of the early hair result in a multiple wing hair phenotype (e.g., *multiple wing hair*; [28]). *frizzled* or *fat* – *dachsous* pathway mutants are characterized by loss of distal cell orientation resulting in whorls of the wing hairs [12]. Mutants for later acting genes result in a loss of parallel hair alignment (e.g., *Gli*, *vari*) [16,17]. Reducing *sqh* or *zip* resulted in frequent multiple wing hairs but no instances of *frizzled*-like whorls, which is consistent with previously published mutant and transgenic perturbations of *sqh* and *zip* [27,29]. In addition, our findings revealed a misaligned hair phenotype reminiscent of the *Gli*, *cora*, and *vari* loss-of-function phenotypes that originate during hair extension. Although the misalignment phenotype was subtle and often obscured by other hair and wing phenotypes, the involvement of *sqh* and *zip* in hair alignment was reinforced by their dominant enhancement of the *Gli* misalignment phenotype. As such, these mutant phenotypes indicate that *sqh* and *zip* have roles in hair development during both hair initiation and hair extension.

The dual role of Sqh and Zip during hair development is further supported by the shift in their localization during hair growth. At hair initiation, we found that both Sqh-GFP and Zip-GFP are diffuse apically, with subtle accumulations concentrating distally (see also [29]). In contrast, during hair extension, Sqh and Zip have accumulated at the apical cell surface, surrounding the base of the developing hair.

In addition to Sqh and Zip, several other myosins have documented roles in hair initiation through *frizzled* or *fat – dachsous* signaling [26,40,47–49]. Indeed, we noted a strong multiple wing hair phenotype in wings expressing *ck* or *jar* RNAi and a lower penetrant phenotype in wings expressing RNAi targeting *dachs*, *MhcI*, *Myo31DF*, *Myo95E*, and *Myo10A*. In contrast, none of the RNAi knockdown genotypes exhibited wing hair misalignment, although it is possible that the strong *ck* multiple hair phenotype obscured a loss of parallel alignment. This suggests that, unlike Sqh and Zip, these other myosins do not have roles in both hair initiation and parallel alignment. As caveats, it is possible that the knockdown of a required gene was not strong enough to induce a misalignment phenotype or that a gene is involved in, but not individually required for, parallel alignment. Given these possibilities, follow-up studies using a variety of alleles, overexpression assays, and genetic interaction tests are warranted. In particular, *ck* and *jar* are intriguing candidates given their strong multiple wing hair phenotype and their documented opposing effects to *sqh* and *zip* during hair initiation [27,29,47]. Notably, in both *D. melanogaster* and vertebrate auditory structures, Zip/MYH9 and Ck/MYO7A genetically and physically interact, and mutations in both *MYH9* and *MYO7A* are associated with deafness in humans [50,51].

### Novel effectors of Gli-dependent wing hair alignment

**Fig 6:**
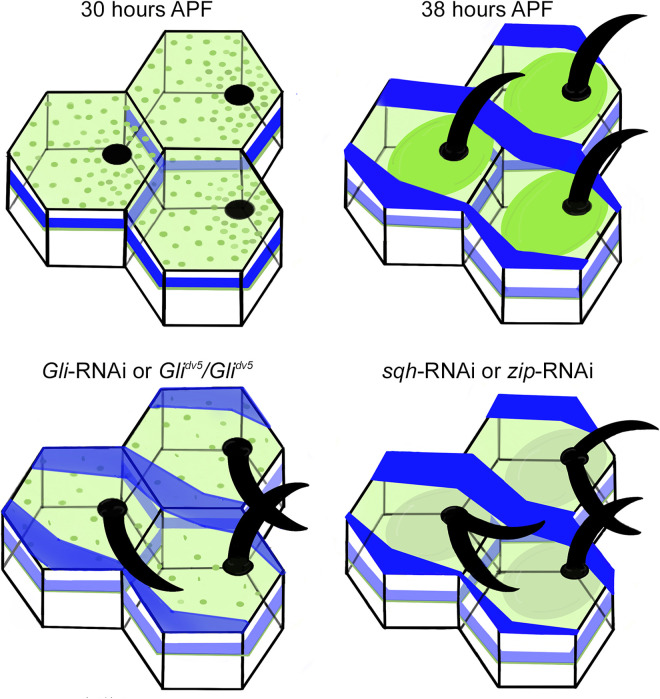
A summary model of Myo-II in wing hair development. At 30 hours APF, when hair development initiates, Dlg (blue) is restricted to the cell periphery and Sqh and Zip (green) are present around the cell periphery and diffusely at the apical cell surface. By 38 hours APF, during hair extension, Dlg localizes in apical proximal-distal ribbons, with lower levels persisting around the cell periphery. Zip and Sqh accumulate at the apical cell surface in circular disks around the base of the developing hairs. In wings with reduced *Gli*, Dlg ribbons are weakly present or absent, Sqh and Zip are diffuse at the apical cell surface, and wing hairs do not maintain parallel alignment. In wings with reduced *sqh* or *zip* that maintain normal cell morphology, Dlg localizes in apical ribbons, but wing hairs exhibit both multiple wing hair and hair misalignment phenotypes.

Despite a parallel alignment mechanism first predicted by Wong and Adler in 1993 [12] and the discovery of Gli and Cora in 2004 [17], our study implicating Sqh and Zip is the first to identify downstream effectors of Gli-dependent wing hair alignment. Based on the genetic interactions between *Gli* and both *sqh* and *zip* and the Gli-dependent localization of Sqh and Zip to the base of developing hairs, we propose a model in which Gli – and perhaps the full septate junction complex – recruits or stabilizes Sqh and Zip at the apical cell surface (Fig 6). The apical Myo-II complex then stabilizes the hair base, maintaining parallel alignment of neighboring wing hairs. Alternatively, it is possible that Gli, via an unknown mechanism, regulates actin at the hair base and actin subsequently recruits Sqh and Zip. Both models are consistent with mutant analysis. Mutant *Gli* or RNAi knockdown results in a loss of apical Sqh and Zip accumulation and leads to unstable hair bases and loss of hair alignment, but does not affect *frizzled*-dependent hair initiation [17]. In contrast, reduced expression of *sqh* or *zip* leads to both multiple wing hair and misalignment phenotypes, reflecting their dual role in both hair initiation and parallel alignment.

Sqh and Zip’s roles in wing and hair development highlight potential reasons other components of the parallel alignment pathway have not yet been identified. As with the septate junction proteins, Sqh and Zip have earlier roles in development which hinder complete loss-of-function analysis of wing hair parallel alignment. Not only are Sqh and Zip required for hair initiation, they are also required for fly viability [33,34] and development of wing tissue [27,29]; this limits use of null alleles and strong wing specific knockdowns. Mosaic clones of *Gli* do not survive [17], and mosaic clones of *sqh* or *zip* either do not survive or are small and outcompeted by neighboring wildtype or heterozygous cells [27,29]. Although we saw evidence of hair misalignment in wing-targeted RNAi knockdown experiments, the phenotype has incomplete penetrance, and stronger knockdowns led to lethality and/or highly penetrant earlier wing phenotypes (e.g., crumpled wing tissue, multiple wing hairs) that mask the misalignment phenotype. Given how few proteins have been identified since Wong and Adler first proposed the presence of a parallel alignment pathway, it is likely that other proteins required for parallel alignment also have earlier, essential roles. Therefore, we suggest that biased and unbiased genetic interaction screens utilizing *Gli* hypomorphs may be helpful methods to identify further components of this mechanism. Since both *frizzled*-dependent hair initiation and *Gli*-dependent parallel alignment mechanisms converge on the F-actin rich wing hairs, strong candidates for biased screens include other cytoskeletal regulators and known effectors of *frizzled*-dependent hair initiation.

### Possible mechanisms of Myo-II and septate junctions in PCP

Our study implicates Myo-II in Gli-mediated hair alignment, but the precise mechanism linking Myo-II to the septate junction remains unknown. One possibility is that septate junction proteins physically bind to Myo-II; past studies have suggested a physical link between the septate junction Neuroglian and Na^+^/K^+^ ATPase proteins and the actin-binding spectrin [52]. However, the lack of colocalization between Dlg and Sqh or Zip at the apical cell surface suggests that direct binding is unlikely. Another possibility supported by recent studies is an indirect septate junction – Myo-II link, with cell recycling and the involvement of the adherens junction E-cadherin emerging as possible mechanisms [45,46,53]. For example, in the pupal notum, loss of septate junction integrity increased junctional and apical Myo-II, likely through increased recycling due to a dysfunctional ESCRT-III complex [45]. Secondly, in both embryonic wound repair and the pupal notum, a change in Myo-II levels triggered by septate junction loss-of-function correlated with a similar shift in E-cadherin [45,46]. Intriguingly, Eaton et al. noted that E-cadherin displayed a similar ribboning pattern to Coracle during hair extension in pupal wing development (although data was not shown) [11]. Further studies are needed to track E-cadherin dynamics during wing hair development and test for a possible correlation between the septate junction, Myo-II, and the adherens junction during establishment of parallel wing hair alignment.

## Conclusion

Although studies in vertebrate and fly tissues hint at Frizzled-independent PCP mechanisms, these remain poorly understood [4,17,54–56]. Utilizing the *D. melanogaster* wing, which exhibits Frizzled-dependent and -independent PCP outputs, we identified a novel Gli-dependent requirement for Myo-II. This Gli-dependent mechanism demonstrates a shared effector between both Frizzled-dependent and -independent mechanisms that had been masked by earlier Myo-II requirements in cell morphogenesis. Given the diversity of processes effected by Myo-II in morphogenesis [3,31], Myo-II and other cytoskeletal regulators serve as strong candidates to mediate multifunctional effector roles in both fly and vertebrate systems. Intriguingly, Neuroligin-3 (the vertebrate Gli homolog), Frizzled, and Myo-II all contribute to differentiation and remodeling of post-synaptic densities of neuronal dendritic spines [57,58]. The complex overlay of Frizzled-dependent and -independent planar cell polarity signaling seen in the *D. melanogaster* wing is likely reflected in analogous events in the vertebrate dendritic spine. Furthermore, our study contributes to an emerging field recognizing non-barrier roles for septate junction proteins in development and homeostasis [42,46,59–63]. Knockdown of components of the vertebrate occluding tight junction, specifically claudin-1 and occludin, leads to impaired wound healing, at least in part through non-barrier mechanisms [64,65]. Given the documented tight junction – actomyosin links [66,67], we envision a prominent role for Myo-II in non-occluding mechanisms in both fly and vertebrate systems.

## Materials and methods

### *D. melanogaster* strains and genetics

All fly strains and cross schemes were maintained according to standard conditions at 25°C, unless otherwise indicated. Mutant alleles were maintained in a *w*^*1118*^ background over CyO, GMR > YFP or FM7c, Kr-GFP marked balancers (RRID:BDSC_5193 and RRID:BDSC_23230). The following mutant alleles were tested: *sqh*^*AX3*^ (RRID:BDSC_25712; [68]), *zip*^*1*^ (RRID:BDSC_4199), *Gli*^*dv5*^ [17], and *Gli*^*RAR77*^ (RRID:BDSC_25142; [17]). Zip-GFP (P{PTT-GC}zip[CC01626], RRID:BDSC_51564; [43]) and Sqh-GFP (P{sqh-GFP.RLC}, RRID:BDSC_57145; [44]) were used to test the pupal wing localization patterns of Zip and Sqh, respectively. For wing-targeted expression of transgenes, *bx*-Gal4; UAS-*dcr* (RRID:BDSC_25706, aka *ms1096*-Gal4) and UAS-*dcr*; *nub*-Gal4 (RRID:BDSC_25754) were used as drivers. For knockdown studies of *sqh* and *zip*, results from UAS-*sqh*-RNAi (RRID:BDSC_33892) and UAS-*zip*-RNAi (RRID:BDSC_37480) are shown. Other lines were tested, but led to pupal lethality (RRID:BDSC_31542 and RRID:BDSC_32439 for *sqh* and RRID:BDSC_36727 and RRID:BDSC_65947 for *zip*) and were not further utilized. UAS-*Gli*-RNAi (RRID:BDSC_58115) was used for knockdown of *Gli*. The following transgenes were used for Myosin knock-down experiments: UAS-*Mhc*-RNAi (RRID:BDSC_26299), UAS-*didum*-RNAi (RRID:BDSC_55740), UAS-*Myo61F*-RNAi (RRID:BDSC_41689), UAS-*jar*-RNAi (RRID:BDSC_28064), UAS-*Mhcl*-RNAi (RRID:BDSC_51456), UAS-*Myo10A*-RNAi (RRID:BDSC_41691), UAS-*Myo95E*-RNAi (RRID:BDSC_62967), UAS-*Myo31DF*-RNAi (RRID:BDSC_33971), UAS-*Myo28B1*-RNAi (RRID:BDSC_41717), UAS-*dachs*-RNAi (RRID:BDSC_27664), and UAS-*ck*-RNAi (RRID:BDSC_41690).

### Adult wing hair observation and statistical analysis

To prevent overcrowding, crosses were set up with 12–15 virgin females and 5–8 males and were flipped to fresh food every 2–3 days. F1 flies were collected within 48 hours of eclosing and aged to 3–5 days. Wings were mounted and analyzed as previously described [17]. Briefly, wings were rinsed in 95% ethanol followed by 85% lactic acid and then mounted in 85% lactic acid. Slides were sealed with nail polish and imaged using a Nikon Eclipse E200 microscope and a Nikon Digital Sight DS-Fi2 camera (and Nikon DS-L3 control unit). For qualitative assessment, a minimum of 10 wings per genotype were analyzed. Images were processed using ImageJ and assembled with Adobe Photoshop.

For quantification of parallel hair alignment in RNAi knockdown experiments (Fig 1 and Table 1), severe wing folding in some mutants prevented quantification of full wings. Therefore, a rectangular area encompassing 50 hairs was randomly selected from a flat portion of each wing and the number of misaligned hairs was counted (misalignment was defined as being greater than or equal to 30 degrees different than immediate neighbors). The data did not meet the normality conditions of parametric tests such as t-tests and ANOVA. Therefore, non-parametric tests were employed. Full documentation of the statistical methods is outlined in the RNAi Data Analysis Report (S4 File) and Data repository [69]. In brief, the statistical analysis was carried out in R version 4.3.2 with the packages tidyverse v2.0.0 (cleaning and manipulating data) [70], readxl v1.4.3 (loading wing scores data from excel) [71], ggplot2 v3.5.2 (making histogram and boxplots) [72], FSA v0.9.5 (performing Dunn’s test) [73], and latex2exp v0.9.6 (labeling boxplot x-axis labels) [74]. The IDE used was RStudio 2024.12.0 (Build 467) [75]. RNAi values were compared to their temperature-matched lacZ controls. For 18°C, which required multiple pair analyses, a Kruskal-Wallis test was conducted to detect any significant differences between the medians. This was followed up with a post-hoc Dunn’s test with a Bonferonni correction for multiple comparison adjustment. For 22°C, 25°C, and 29°C, which only required single pair analyses, Mann-Whitney U tests were conducted.

For quantification of parallel hair alignment for genetic interaction experiments (Fig 2), each wing was divided into seven regions using the veins as boundaries (Fig 2A) and the number of regions that displayed patches of misaligned hairs was counted (the “hair alignment score” or simply “score” for brevity) [17,69]. A misaligned patch was defined as two or more “chevrons” (with a chevron formed by two neighboring hairs tilted towards one another) and/or at least four individual tilted hairs within a region. Due to variable viability of the relevant genotypes, samples ranged from 18 to 90 wings. For consistency and to remove bias, all scoring was done by a single individual who was blinded to the genotypes. The data is based on an ordinal scale and during our analyses did not meet the normality conditions of parametric tests such as t-tests and ANOVA. Thus, non-parametric alternatives were used as appropriate statistical tests to detect significant changes in the hair alignment scores. Full documentation of the statistical methods is outlined in the Interaction Data Analysis Report (S5 File) and Data repository [69].

In brief, the statistical analysis was carried out in R version 4.3.2 [76] with the packages tidyverse v2.0.0 (cleaning and manipulating data) [70], readxl v1.4.3 (loading wing scores data from excel) [71], ggplot2 v3.5.1 (making histogram and boxplots) [77], FSA v0.10.0 (performing Dunn’s test) [73], and latex2exp v0.9.6 (labeling plot x-axis labels) [74]. The IDE used was RStudio 2023.09.1 (Build 494) [78]. The most appropriate measure on which to focus our analysis was the median scores. Filtering by the three base *Gli* genotypes (*Gli*^*dv5*^/ + , *Gli*^*dv5*^/*Gli*^*RAR77*^, and *Gli*^*dv5*^/*Gli*^*dv5*^), a Kruskal-Wallis test was conducted to detect any significant differences between the median scores. This was followed up with a post-hoc Dunn’s test with a Bonferonni correction for multiple comparison adjustment. The p-values reported in Fig 2 are the adjusted p-values from the Dunn’s test. These tests establish the significance in the differences between the scores of each *Gli* genotype alone and in combination with *sqh*^*AX3*^/+ or *zip*^*1*^/+ (as indicated by the brackets in Fig 2M).

To directly test if observed differences were more than additive (i.e., synergistic), we employed a non-parametric bootstrap approach (adapted from [79]). Bootstrapping is a robust and straightforward method to compute a null distribution of the purely additive effect; this allows for a comparison of our observed test statistic to determine if the observed effect in our original data was more than additive and if the effect was statistically significant. We generated a stratified bootstrap sample for each of the three base *Gli* genotypes (*Gli*^*dv5*^/ + , *Gli*^*dv5*^/*Gli*^*RAR77*^, and *Gli*^*dv5*^/*Gli*^*dv5*^) and for the *sqh* and *zip* heterozygotes (*sqh*^*AX3*^/+ and *zip*^*1*^/+). Next, we added the bootstrapped score values of *sqh*^*AX3*^/+ with each *Gli* base genotype to get the expected additive score of each pair. The process was repeated with *zip*^*1*^/ + . (We only computed as many scores as there were in the original dataset. For example, the original dataset had 18 scores for *sqh*^*AX3*^/ + ; *Gli*^*dv5*^/*Gli*^*dv5*^ so only 18 scores were computed in the bootstrap sample). This provided us with a simulated dataset that corresponds to the scenario that the effect is purely additive. To compute the test statistic, we calculated the median score of each genotype that paired *Gli*^*dv5*^/ + , *Gli*^*dv5*^/*Gli*^*RAR77*^, or *Gli*^*dv5*^/*Gli*^*dv5*^ with *sqh*^*AX3*^/+ or *zip*^*1*^/+ (e.g. *sqh*^*AX3*^/ + ; *Gli*^*dv5*^/+). This bootstrapping procedure was repeated 10,000 times to generate a null distribution of the test statistic (the median score of each simulated additive genotype). Finally, each p-value was calculated as the number of simulated bootstrap samples with median scores greater than or equal to the actual scores of the corresponding genotype. Six p-values were computed, one for each of the following genotypes: *sqh*^*AX3*^/ + ; *Gli*^*dv5*^/ + , *sqh*^*AX3*^/ + ; *Gli*^*dv5*^/*Gli*^*RAR77*^, *sqh*^*AX3*^/ + ; *Gli*^*dv5*^/*Gli*^*dv5*^*, zip*^*1*^*/ + ; Gli*^*dv5*^/ + , *zip*^*1*^*/ + ; Gli*^*dv5*^/*Gli*^*RAR77*^, and *zip*^*1*^*/ + ; Gli*^*dv5*^/*Gli*^*dv5*^.

### Pupal wing immunohistochemistry, imaging, and statistical analysis

To ensure consistent density and growth rates, crosses were set up with 12–15 virgin females and 5–8 males and were flipped to fresh food every 2–3 days and cultured at 25°C. Pupae at 0 hours APF were manually identified and aged as indicated. At the indicated age, the pupal case operculum and the head capsule were removed to permit penetration of fixative into the tissue. The pupae were fixed in 4% formaldehyde in 1x PBS for 20 minutes at room temperature. Wing tissue was dissected in 1x PBS + 0.1% Triton-X 100.

All immunolabeling steps were completed in 1x PBS + 0.1% Triton-X 100. After dissection, wings were incubated with 1:15 Normal Goat Serum (NGS, Sigma) for 30 minutes at room temperature, followed by primary antibody overnight at 4°C. Wings were washed three times for 10 minutes at room temperature, followed by a second incubation with 1:15 NGS for 30 minutes at room temperature and with secondary antibody and Phalloidin for 2 hours at room temperature. If used, To-Pro-3 Iodine (1:1,500, Invitrogen) was added for the last 20 minutes to label cell nuclei. Wings were then washed three times for 10 minutes at room temperature, followed by an overnight wash at 4°C. Mouse monoclonal anti-Dlg (1:30, Developmental Studies Hybridoma Bank 4F3 anti-discs large, RRID:AB_528203) and chicken polyclonal anti-GFP (1:1,000, Abcam ab13970, RRID:AB_300798) were used as primary antibodies. Alexa Fluor 488 and 647 secondary antibodies were used at a 1:150 dilution (Thermo Fisher Scientific A-11039, RRID:AB_2534096 and A-11029, RRID:AB_2534088 and A-21236, RRID:AB_2535805). F-actin was labeled with Alexa Fluor 555 Phalloidin or 647 Phalloidin (1:100, Invitrogen).

Wings were mounted in Vectashield (Vector Laboratories) and imaged using a Leica SP8 confocal microscope. Z-stacks were acquired every 0.33 µm. XY images shown in this manuscript are single slices selected from a full Z-stack. XZ images are selections from the Z-stack using the orthogonal view tool in Leica’s LAS X software. Images were processed using ImageJ and assembled with Adobe Photoshop.

To quantify the localization of Dlg in proximal-distal ribbons, we compared the fluorescence intensity of Dlg at the anterior and posterior cell boundaries (which run along the proximal-distal wing axis) to the proximodistal cell boundaries. Single Z-slices were selected at the most apical position anti-Dlg was clearly detected. For each cell sampled, ImageJ was used to calculate the Mean Gray Value of rectangular Regions of Interest (ROI) for both the anterior and posterior cell boundaries, and their respective neighboring proximodistal cell boundaries positioned immediately clockwise. From these four values, an average AP/PD ratio per cell was calculated. Five cells per wing were sampled and averaged to give an AP/PD ratio per wing.

To quantify Sqh and Zip localization in apical circular accumulations, single Z-slices were selected at the most apical position anti-GFP (Sqh-GFP or Zip-GFP) was clearly detected. For each cell sampled, ImageJ was used to calculate the Mean Gray Value and Area for a circular ROI encompassing the apical cell center (35 pixel diameter, equivalent to ~3.2 µm) and a larger circular ROI including both the cell center and the adjacent periphery (45 pixel diameter, equivalent to ~4.1µm). From these, the Mean Gray Value for the periphery was calculated. This was then used to calculate an intensity ratio of the cell center relative to the periphery (CC/CP). Five cells per wing were sampled and averaged to give an CC/CP ratio per wing.

Full documentation of the calculations and statistical methods are outlined in the Dlg and Sqh and Zip Data Analysis Reports (S6 File and S7 File) and Data repository [69]. In brief, the statistical analysis for Dlg, Sqh, and Zip localization was carried out in R version 4.3.2 with the packages tidyverse v2.0.0 (cleaning and manipulating data) [70], readxl v1.4.3 (loading wing scores data from excel) [71], ggplot2 v3.5.2 (making histogram and boxplots) [72], FSA v0.10.0 (performing Dunn’s test) [73], and latex2exp v0.9.6 (labeling boxplot x-axis labels) [74]. The IDE used was RStudio 2024.12.0 (Build 467) [75]. The Zip analysis (including data from two genotypes) satisfied the assumptions of Welch’s 2 sample t-test since wing samples were independent, the population of ratios is assumed to be normal, and the variance of the ratios for each genotype was roughly the same (Bartlett’s test p-value = 0.7376). Therefore, statistical significance was calculated using Welch’s 2 sample t-test. The Sqh and Dlg analyses (including data from four genotypes) both satisfied the assumptions of a one-way ANOVA model since wing samples were independent, the residuals of the ANOVA model were roughly normally distributed and the variance of the ratios for each genotype was roughly the same (Bartlett’s test p-value = 0.532 for Dlg, 0.1274 for Sqh). Therefore, statistical significance for Dlg and Sqh comparisons were calculated using one-way ANOVA followed by a Tukey’s HSD test.

## Supporting information

S1 Fig A subset of *D. melanogaster* myosins are required for wing hair development. Expression of myosin genes was reduced by wing targeted RNAi knockdown using Bx-Gal4. Expression of *lacZ* (A) or RNAi targeting *dachs* (B), *didum* (C), *Mhc* (D), *Mhcl* (E), *Myo28B1* (F), *Myo31DF* (G), *Myo61F* (H), and *Myo95E* (I) did not result in consistent wing hair polarity phenotypes. Occasional multiple wing hairs were observed in wings with reduced *dachs*, *Mhcl*, *Myo31DF*, or *Myo95E* (asterisks). Wings with reduced *crinkled* (J, J’) exhibit a highly penetrant multiple wing hair phenotype, with 2–6 short hairs splaying from the hair base. Knockdown of *jaguar* (K, K’) resulted in a multiple wing hair phenotype characterized by two (occasionally more) long hairs. Hairs often had a slight curve as the hair tapered (arrow). To clearly visualize hair direction, insets mark the wing hair positions of the top wing layer. Wings with reduced *Myo10A* (L, L’) displayed multiple wing hairs, misdirection of patches of hairs (e.g., see L’), and some overall wing folding (not shown). In all panels except D, wings were collected from male flies grown at 29°C. Due to low male viability, wings in D were collected from female flies grown at 29°C. Calibration: 25 µm.
(TIF)

S2 Fig Dlg relocalizes in the developing wing between 30–46 hours APF. As depicted in the top left panel, select images are apical XY and transverse XZ planes of the wing bilayer. The bilayer is apparent through the two layers of DAPI labeled nuclei (blue). Wing hairs (Phalloidin, red) extend from the apical surface of both the top and bottom cell layers. In developing pupal wings, Dlg (green) is present around the cell periphery at 30 and 34 hours APF. At these time points no hair extension was observed (insets). By 38 hours APF, Dlg relocalizes into continuous apical ribbons and developing hairs (red, inset) extend beyond the apical cell surface. Lower levels of Dlg remain at the cell periphery at the basolateral cell surfaces (see Fig 3B). At 42 hours APF, as the hairs continue to develop, the Dlg ribbons are no longer continuous. By 46 hours APF, Dlg is no longer present in apical ribbons, but is again present only around the cell periphery. Calibration: 10 µm.
(TIF)

S3 Fig Zip-GFP in wildtype and Sqh-GFP in Gli-RNAi wings at 38 hours APF. (A) Enlarged XY images of the boxed region in Fig 3D shown at descending 1 µm Z intervals. The 0 µm position is set immediately above the apical cell surface. Zip-GFP (−1 µm) is present in circular accumulations near the apical cell surface. Dlg (−2 to −4 µm) forms ribbons below the apical surface of the wing. Both Zip-GFP and Dlg are present around the cell periphery basolaterally (−5 to −6 µm). Calibration: 5 µm. (B-C) Apical (XY) and transverse (XZ) sections of pupal wings at 38 hours APF. The dotted lines in the XZ images indicate the positions of the XY planes. In Bx-Gal4 driven UAS-*lacZ* control wings, Dlg (blue) accumulates in apical ribbons at 38 hours APF, with minimal to no Dlg present at proximodistal cell boundaries (B’; dotted parallel lines track three example ribbons). Immediately apical to these ribbons, Sqh-GFP (green) is present in circular accumulations (B; dotted ellipses outline example accumulations). In Bx-Gal4 driven UAS-*Gli*-RNAi wings, Dlg does not form continuous ribbons, but remains around the lateral cell surface (C’). Arrowheads indicate residual accumulation at the proximodistal cell boundaries. Unlike in wildtype, Sqh does not accumulate apically, but remains diffuse at the apical surface (C). Calibration: 10 µm.
(TIF)

S4 File Supplemental Material 4: RNAi Data Analysis Report. A summary document of the statistical analyses used to quantify the *sqh*-RNAi and *zip*-RNAi hair misalignment phenotypes (as summarized in Table 1).
(PDF)

S5 File Supplemental Material 5: Interaction Data Analysis Report. A summary document of the statistical analyses used to test for a genetic interaction between *Gli* and *sqh* or *zip* (as summarized in Fig 2M).
(PDF)

S6 File Supplemental Material 6: Dlg Data Analysis Report. A summary document of the statistical analyses used to quantify Dlg localization (as summarized in Fig 4E).
(PDF)

S7 File Supplemental Material 7: Sqh and Zip Data Analysis Report. A summary document of the statistical analyses used to quantify Sqh and Zip localization (as summarized in Fig 4F-G).
(PDF)

## References

[1] Butler MT, Wallingford JB. Planar cell polarity in development and disease. Nat Rev Mol Cell Biol. 2017;18(6):375–88. doi: 10.1038/nrm.2017.11 28293032 PMC5826606

[2] Derish I, Lee JKH, Wong-King-Cheong M, Babayeva S, Caplan J, Leung V, et al. Differential role of planar cell polarity gene Vangl2 in embryonic and adult mammalian kidneys. PLoS One. 2020;15(3):e0230586. doi: 10.1371/journal.pone.0230586 32203543 PMC7089571

[3] Devenport D. Tissue morphodynamics: Translating planar polarity cues into polarized cell behaviors. Semin Cell Dev Biol. 2016;55:99–110. doi: 10.1016/j.semcdb.2016.03.012 26994528 PMC5005802

[4] Hirota Y, Meunier A, Huang S, Shimozawa T, Yamada O, Kida YS. Planar polarity of multiciliated ependymal cells involves the anterior migration of basal bodies regulated by non-muscle myosin II. Development. 2010;137(18):3037–46. 20685736 10.1242/dev.050120

[5] Kunimoto K, Bayly RD, Vladar EK, Vonderfecht T, Gallagher A-R, Axelrod JD. Disruption of Core Planar Cell Polarity Signaling Regulates Renal Tubule Morphogenesis but Is Not Cystogenic. Curr Biol. 2017;27(20):3120-3131.e4. doi: 10.1016/j.cub.2017.09.011 29033332 PMC5683414

[6] Mirzadeh Z, Han YG, Soriano-Navarro M, García-Verdugo JM, Alvarez-Buylla A. Cilia Organize Ependymal Planar Polarity. J Neurosci. 2010;30(7):2600–10. 20164345 10.1523/JNEUROSCI.3744-09.2010 PMC2873868

[7] Tarchini B, Lu X. New insights into regulation and function of planar polarity in the inner ear. Neurosci Lett. 2019;709:134373. doi: 10.1016/j.neulet.2019.134373 31295539 PMC6732021

[8] Goodrich LV, Strutt D. Principles of planar polarity in animal development. Development. 2011;138(10):1877–92. doi: 10.1242/dev.054080 21521735 PMC3082295

[9] Peng Y, Axelrod JD. Asymmetric protein localization in planar cell polarity: mechanisms, puzzles, and challenges. Curr Top Dev Biol. 2012;101:33–53. doi: 10.1016/B978-0-12-394592-1.00002-8 23140624 PMC4854750

[10] Yang Y, Mlodzik M. Wnt-Frizzled/planar cell polarity signaling: cellular orientation by facing the wind (Wnt). Annu Rev Cell Dev Biol. 2015;31:623–46. doi: 10.1146/annurev-cellbio-100814-125315 26566118 PMC4673888

[11] Eaton S, Wepf R, Simons K. Roles for Rac1 and Cdc42 in planar polarization and hair outgrowth in the wing of Drosophila. J Cell Biol. 1996;135(5):1277–89. doi: 10.1083/jcb.135.5.1277 8947551 PMC2121092

[12] Wong LL, Adler PN. Tissue polarity genes of Drosophila regulate the subcellular location for prehair initiation in pupal wing cells. J Cell Biol. 1993;123(1):209–21. doi: 10.1083/jcb.123.1.209 8408199 PMC2119819

[13] Strutt H, Strutt D. How do the fat–Dachsous and core planar polarity pathways act together and independently to coordinate polarized cell behaviours?. Open Biology. 2021;11(2):200356. 33561385 10.1098/rsob.200356 PMC8061702

[14] Adler PN, Charlton J, Liu J. Mutations in the cadherin superfamily member gene dachsous cause a tissue polarity phenotype by altering frizzled signaling. Development. 1998;125(5):959–68. doi: 10.1242/dev.125.5.959 9449678

[15] Gubb D, García-Bellido A. A genetic analysis of the determination of cuticular polarity during development in Drosophila melanogaster. J Embryol Exp Morphol. 1982;68:37–57. 6809878

[16] Moyer KE, Jacobs JR. Varicose: a MAGUK required for the maturation and function of Drosophila septate junctions. BMC Dev Biol. 2008;8:99. doi: 10.1186/1471-213X-8-99 18847477 PMC2575209

[17] Venema DR, Zeev-Ben-Mordehai T, Auld VJ. Transient apical polarization of Gliotactin and Coracle is required for parallel alignment of wing hairs in Drosophila. Dev Biol. 2004;275(2):301–14. doi: 10.1016/j.ydbio.2004.07.040 15501220

[18] Guild GM, Connelly PS, Ruggiero L, Vranich KA, Tilney LG. Actin filament bundles in Drosophila wing hairs: hairs and bristles use different strategies for assembly. Mol Biol Cell. 2005;16(8):3620–31. doi: 10.1091/mbc.e05-03-0185 15917291 PMC1182302

[19] Ren N, Zhu C, Lee H, Adler PN. Gene expression during Drosophila wing morphogenesis and differentiation. Genetics. 2005;171(2):625–38. 15998724 10.1534/genetics.105.043687 PMC1456776

[20] Strutt DI. Asymmetric localization of frizzled and the establishment of cell polarity in the Drosophila wing. Mol Cell. 2001;7(2):367–75. doi: 10.1016/s1097-2765(01)00184-8 11239465

[21] Axelrod JD. Unipolar membrane association of Dishevelled mediates Frizzled planar cell polarity signaling. Genes Dev. 2001;15(10):1182–7. doi: 10.1101/gad.890501 11358862 PMC313798

[22] Bastock R, Strutt H, Strutt D. Strabismus is asymmetrically localised and binds to Prickle and Dishevelled during Drosophila planar polarity patterning. Development. 2003;130(13):3007–14. doi: 10.1242/dev.00526 12756182

[23] Feiguin F, Hannus M, Mlodzik M, Eaton S. The ankyrin repeat protein Diego mediates frizzled-dependent planar polarization. Developmental Cell. 2001;1(1):93–101. 11703927 10.1016/s1534-5807(01)00010-7

[24] Tree DRP, Shulman JM, Rousset R, Scott MP, Gubb D, Axelrod JD. Prickle mediates feedback amplification to generate asymmetric planar cell polarity signaling. Cell. 2002;109(3):371–81. doi: 10.1016/s0092-8674(02)00715-8 12015986

[25] Usui T, Shima Y, Shimada Y, Hirano S, Burgess RW, Schwarz TL, et al. Flamingo, a seven-pass transmembrane cadherin, regulates planar cell polarity under the control of Frizzled. Cell. 1999;98(5):585–95. doi: 10.1016/s0092-8674(00)80046-x 10490098

[26] Turner CM, Adler PN. Distinct roles for the actin and microtubule cytoskeletons in the morphogenesis of epidermal hairs during wing development in Drosophila. Mechanisms of Development. 1998;70(1):181–92. 9510034 10.1016/s0925-4773(97)00194-9

[27] Franke JD, Montague RA, Kiehart DP. Nonmuscle myosin II is required for cell proliferation, cell sheet adhesion and wing hair morphology during wing morphogenesis. Dev Biol. 2010;345(2):117–32. doi: 10.1016/j.ydbio.2010.06.028 20599890 PMC3712330

[28] Lu Q, Schafer DA, Adler PN. The Drosophila planar polarity gene multiple wing hairs directly regulates the actin cytoskeleton. Development. 2015;142(14):2478–86. 26153232 10.1242/dev.122119 PMC4510862

[29] Winter CG, Wang B, Ballew A, Royou A, Karess R, Axelrod JD, et al. Drosophila Rho-associated kinase (Drok) links Frizzled-mediated planar cell polarity signaling to the actin cytoskeleton. Cell. 2001;105(1):81–91. doi: 10.1016/s0092-8674(01)00298-7 11301004

[30] Yan J, Lu Q, Fang X, Adler PN. Rho1 has multiple functions in Drosophila wing planar polarity. Dev Biol. 2009;333(1):186–99. doi: 10.1016/j.ydbio.2009.06.027 19576201 PMC2728161

[31] Quintanilla MA, Hammer JA, Beach JR. Non-muscle myosin 2 at a glance. J Cell Sci. 2023;136(5):jcs260890. doi: 10.1242/jcs.260890 36917212 PMC10411949

[32] Vicente-Manzanares M, Ma X, Adelstein RS, Horwitz AR. Non-muscle myosin II takes centre stage in cell adhesion and migration. Nat Rev Mol Cell Biol. 2009;10(11):778–90. doi: 10.1038/nrm2786 19851336 PMC2834236

[33] Karess RE, Chang XJ, Edwards KA, Kulkarni S, Aguilera I, Kiehart DP. The regulatory light chain of nonmuscle myosin is encoded by spaghetti-squash, a gene required for cytokinesis in Drosophila. Cell. 1991;65(7):1177–89. doi: 10.1016/0092-8674(91)90013-o 1905980

[34] Young PE, Richman AM, Ketchum AS, Kiehart DP. Morphogenesis in Drosophila requires nonmuscle myosin heavy chain function. Genes Dev. 1993;7(1):29–41. doi: 10.1101/gad.7.1.29 8422986

[35] Lunde K, Biehs B, Nauber U, Bier E. The knirps and knirps-related genes organize development of the second wing vein in Drosophila. Development. 1998;125(21):4145–54. doi: 10.1242/dev.125.21.4145 9753669

[36] Men TT, Binh TD, Yamaguchi M, Huy NT, Kamei K. Function of Lipid Storage Droplet 1 (Lsd1) in Wing Development of Drosophila melanogaster. Int J Mol Sci. 2016;17(5):648. doi: 10.3390/ijms17050648 27136547 PMC4881474

[37] Tortoriello G, de Celis JF, Furia M. Linking pseudouridine synthases to growth, development and cell competition. FEBS J. 2010;277(15):3249–63. doi: 10.1111/j.1742-4658.2010.07731.x 20608977

[38] Azpiazu N, Morata G. Function and regulation of homothorax in the wing imaginal disc of Drosophila. Development. 2000;127(12):2685–93. 10821766 10.1242/dev.127.12.2685

[39] del Álamo Rodríguez D, Terriente Felix J, Díaz-Benjumea FJ. The role of the T-box gene optomotor-blind in patterning the Drosophila wing. Developmental Biology. 2004;268(2):481–92. 15063183 10.1016/j.ydbio.2004.01.005

[40] Kiehart DP, Franke JD, Chee MK, Montague RA, Chen T-L, Roote J, et al. Drosophila crinkled, mutations of which disrupt morphogenesis and cause lethality, encodes fly myosin VIIA. Genetics. 2004;168(3):1337–52. doi: 10.1534/genetics.104.026369 15579689 PMC1448781

[41] Auld VJ, Fetter RD, Broadie K, Goodman CS. Gliotactin, a novel transmembrane protein on peripheral glia, is required to form the blood-nerve barrier in Drosophila. Cell. 1995;81(5):757–67. 7539719 10.1016/0092-8674(95)90537-5

[42] Rice C, De O, Alhadyian H, Hall S, Ward RE. Expanding the junction: new insights into non-occluding roles for septate junction proteins during development. Journal of Developmental Biology. 2021;9(1):11. 33801162 10.3390/jdb9010011 PMC8006247

[43] Buszczak M, Paterno S, Lighthouse D, Bachman J, Planck J, Owen S, et al. The carnegie protein trap library: a versatile tool for Drosophila developmental studies. Genetics. 2007;175(3):1505–31. doi: 10.1534/genetics.106.065961 17194782 PMC1840051

[44] Royou A, Field C, Sisson JC, Sullivan W, Karess R. Reassessing the role and dynamics of nonmuscle myosin II during furrow formation in early Drosophila embryos. Mol Biol Cell. 2004;15(2):838–50. doi: 10.1091/mbc.e03-06-0440 14657248 PMC329397

[45] Esmangart de Bournonville T, Jaglarz MK, Durel E, Le Borgne R. ESCRT-III-dependent adhesive and mechanical changes are triggered by a mechanism detecting alteration of septate junction integrity in Drosophila epithelial cells. Elife. 2024;13:e91246. doi: 10.7554/eLife.91246 38305711 PMC10959524

[46] Carvalho L, Patricio P, Ponte S, Heisenberg C-P, Almeida L, Nunes AS, et al. Occluding junctions as novel regulators of tissue mechanics during wound repair. J Cell Biol. 2018;217(12):4267–83. doi: 10.1083/jcb.201804048 30228162 PMC6279375

[47] Djiane A, Mlodzik M. The Drosophila GIPC homologue can modulate myosin based processes and planar cell polarity but is not essential for development. PLoS One. 2010;5(6):e11228. doi: 10.1371/journal.pone.0011228 20574526 PMC2888583

[48] Lin C, Katanaev VL. Kermit interacts with Gαo, Vang, and motor proteins in Drosophila planar cell polarity. PLoS One. 2013;8(10):e76885. doi: 10.1371/journal.pone.0076885 24204696 PMC3805608

[49] Mao Y, Rauskolb C, Cho E, Hu W-L, Hayter H, Minihan G, et al. Dachs: an unconventional myosin that functions downstream of Fat to regulate growth, affinity and gene expression in Drosophila. Development. 2006;133(13):2539–51. doi: 10.1242/dev.02427 16735478

[50] Li T, Bellen HJ, Groves AK. Using Drosophila to study mechanisms of hereditary hearing loss. Dis Model Mech. 2018;11(6):dmm031492. doi: 10.1242/dmm.031492 29853544 PMC6031363

[51] Li T, Giagtzoglou N, Eberl DF, Jaiswal SN, Cai T, Godt D, et al. The E3 ligase Ubr3 regulates Usher syndrome and MYH9 disorder proteins in the auditory organs of Drosophila and mammals. Elife. 2016;5:e15258. doi: 10.7554/eLife.15258 27331610 PMC4978524

[52] Dubreuil RR. Functional links between membrane transport and the spectrin cytoskeleton. J Membr Biol. 2006;211(3):151–61. doi: 10.1007/s00232-006-0863-y 17091212

[53] De O, Rice C, Zulueta-Coarasa T, Fernandez-Gonzalez R, Ward RE 4th. Septate junction proteins are required for cell shape changes, actomyosin reorganization and cell adhesion during dorsal closure in Drosophila. Front Cell Dev Biol. 2022;10:947444. doi: 10.3389/fcell.2022.947444 36238688 PMC9553006

[54] Cetera M, Leybova L, Woo FW, Deans M, Devenport D. Planar cell polarity-dependent and independent functions in the emergence of tissue-scale hair follicle patterns. Dev Biol. 2017;428(1):188–203. doi: 10.1016/j.ydbio.2017.06.003 28599846 PMC5549468

[55] Kirjavainen A, Laos M, Anttonen T, Pirvola U. The Rho GTPase Cdc42 regulates hair cell planar polarity and cellular patterning in the developing cochlea. Biol Open. 2015;4(4):516–26. doi: 10.1242/bio.20149753 25770185 PMC4400594

[56] Vichas A, Zallen JA. Translating cell polarity into tissue elongation. Semin Cell Dev Biol. 2011;22(8):858–64. doi: 10.1016/j.semcdb.2011.09.013 21983030 PMC4752253

[57] Cohen RS. The Postsynaptic Density. Neuroscience in the 21st Century: From Basic to Clinical. Cham: Springer International Publishing. 2022. p. 751–88.

[58] Voglewede MM, Zhang H. Polarity proteins: Shaping dendritic spines and memory. Dev Biol. 2022;488:68–73. doi: 10.1016/j.ydbio.2022.05.007 35580729 PMC9953585

[59] Hall S, Ward RE 4th. Septate Junction Proteins Play Essential Roles in Morphogenesis Throughout Embryonic Development in Drosophila. G3 (Bethesda). 2016;6(8):2375–84. doi: 10.1534/g3.116.031427 27261004 PMC4978892

[60] Laprise P, Paul SM, Boulanger J, Robbins RM, Beitel GJ, Tepass U. Epithelial polarity proteins regulate Drosophila tracheal tube size in parallel to the luminal matrix pathway. Curr Biol. 2010;20(1):55–61. doi: 10.1016/j.cub.2009.11.017 20022244 PMC2821987

[61] Nelson KS, Furuse M, Beitel GJ. The Drosophila Claudin Kune-kune is required for septate junction organization and tracheal tube size control. Genetics. 2010;185(3):831–9. doi: 10.1534/genetics.110.114959 20407131 PMC2907205

[62] Wang S, Jayaram SA, Hemphälä J, Senti K-A, Tsarouhas V, Jin H, et al. Septate-junction-dependent luminal deposition of chitin deacetylases restricts tube elongation in the Drosophila trachea. Curr Biol. 2006;16(2):180–5. doi: 10.1016/j.cub.2005.11.074 16431370

[63] Holtwick V, Schubert A, Rust K. Distinct Non-occluding Functions of Septate Junction Components in Signaling Pathway Regulation and Cell Polarity During Epithelial Development. BioRxiv [Preprint]. 2024 bioRxiv 2024.09.06.611635 [posted 2024 Sep 7; cited 2025 Jun 13]: [34 p.] Available from: https://www.biorxiv.org/content/10.1101/2024.09.06.611635v1 10.1101/2024.09.06.611635

[64] Volksdorf T, Heilmann J, Eming SA, Schawjinski K, Zorn-Kruppa M, Ueck C, et al. Tight Junction Proteins Claudin-1 and Occludin Are Important for Cutaneous Wound Healing. Am J Pathol. 2017;187(6):1301–12. doi: 10.1016/j.ajpath.2017.02.006 28412298

[65] Safferling K, Sütterlin T, Westphal K, Ernst C, Breuhahn K, James M, et al. Wound healing revised: a novel reepithelialization mechanism revealed by in vitro and in silico models. J Cell Biol. 2013;203(4):691–709. 24385489 10.1083/jcb.201212020 PMC3840932

[66] Sluysmans S, Vasileva E, Spadaro D, Shah J, Rouaud F, Citi S. The role of apical cell-cell junctions and associated cytoskeleton in mechanotransduction. Biol Cell. 2017;109(4):139–61. doi: 10.1111/boc.201600075 28220498

[67] Balda MS, Matter K. Tight junctions as regulators of tissue remodelling. Curr Opin Cell Biol. 2016;42:94–101. doi: 10.1016/j.ceb.2016.05.006 27236618

[68] Jordan P, Karess R. Myosin light chain-activating phosphorylation sites are required for oogenesis in Drosophila. J Cell Biol. 1997;139(7):1805–19. 9412474 10.1083/jcb.139.7.1805 PMC2132636

[69] Mishra A. Statistical Analyses of Wing Hair Alignment. Zenodo. Available from: doi: 10.5281/zenodo.15627916

[70] Wickham H, Averick M, Bryan J, Chang W, McGowan L, François R, et al. Welcome to the Tidyverse. JOSS. 2019;4(43):1686. doi: 10.21105/joss.01686

[71] Wickham H, Bryan J. readxl: Read Excel Files. R package version 1.4.3 [software]. 2023 [cited 2025 Jun 9]. Available from: https://github.com/tidyverse/readxl; https://readxl.tidyverse.org

[72] Wickham H. ggplot2: Elegant Graphics for Data Analysis. Version 3.5.2 [software]. New York: Springer; 2016 [cited 2025 Jun 9]. Available from: https://ggplot2.tidyverse.org

[73] Ogle DH, Doll JC, Wheeler AP, Dinno A. FSA: Simple Fisheries Stock Assessment Methods. R package version 0.10.0 [software]. 2025 [cited 2025 Jun 9]. doi: 10.32614/CRAN.package.FSA Available from: https://CRAN.R-project.org/package=FSA.

[74] Meschiari S. latex2exp: Use LaTeX Expressions in Plots. 2022.

[75] RStudio: Integrated Development Environment for R. Boston (MA): Posit Software, PBC. 2024.

[76] R Core Team. R: A Language and Environment for Statistical Computing. Version 4.3.2 [software]. Vienna (Austria): R Foundation for Statistical Computing; 2023 [cited 2025 Jun 9]. Available from: https://www.R-project.org/.

[77] Wickham H. ggplot2: Elegant Graphics for Data Analysis. Version 3.5.1 [software]. New York: Springer-Verlag; 2016 [cited 2025 Jun 9]. Available from: https://ggplot2.tidyverse.org

[78] Posit team. RStudio: Integrated Development Environment for R. Version 2023.09.1 (Build 494) [software]. Boston (MA): Posit Software, PBC; 2023 [cited 2025 Jun 9]. Available from: https://posit.co/.

[79] Ahmad SM, Tansey TR, Busser BW, Nolte MT, Jeffries N, Gisselbrecht SS, et al. Two forkhead transcription factors regulate the division of cardiac progenitor cells by a Polo-dependent pathway. Dev Cell. 2012;23(1):97–111. doi: 10.1016/j.devcel.2012.05.011 22814603 PMC3401414

