# Supplementary figures and images for: A role for Myo-II zipper and spaghetti squash in Gliotactin-dependent Drosophila melanogaster wing hair planar cell polarity
Source: PLoS One. 2025 Jul 23;20(7):e0328970. doi: 10.1371/journal.pone.0328970 (PMC12286378; doi:10.1371/journal.pone.0328970)

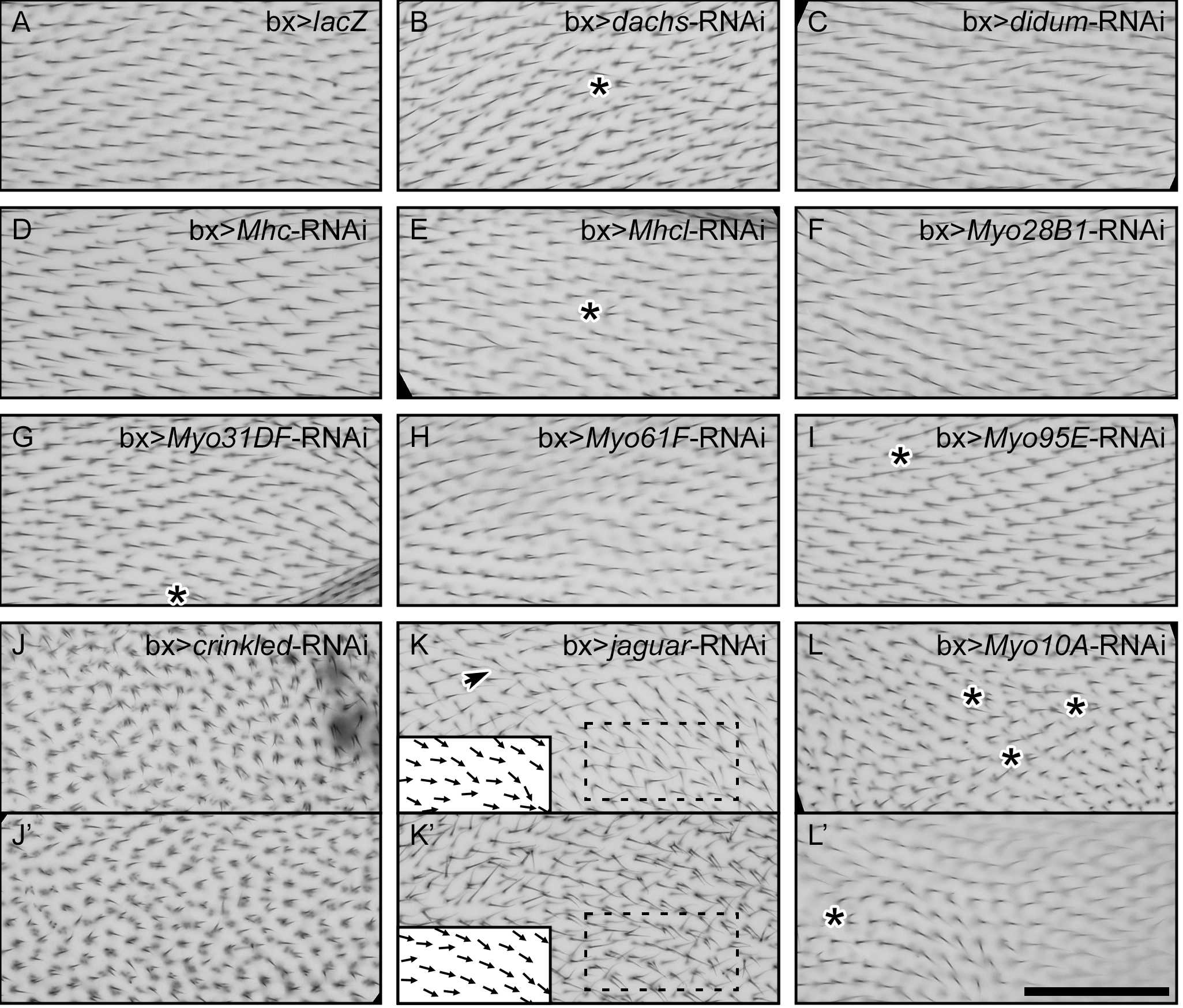

Supplement: S1 Fig — Expression of myosin genes was reduced by wing targeted RNAi knockdown using Bx-Gal4. Expression of lacZ (A) or RNAi targeting dachs (B), didum (C), Mhc (D), Mhcl (E), Myo28B1 (F), Myo31DF (G), Myo61F (H), and Myo95E (I) did not result in consistent wing hair polarity phenotypes. Occasional multiple wing hairs were observed in wings with reduced dachs, Mhcl, Myo31DF, or Myo95E (asterisks). Wings with reduced crinkled (J, J’) exhibit a highly penetrant multiple wing hair phenotype, with 2–6 short hairs splaying from the hair base. Knockdown of jaguar (K, K’) resulted in a multiple wing hair phenotype characterized by two (occasionally more) long hairs. Hairs often had a slight curve as the hair tapered (arrow). To clearly visualize hair direction, insets mark the wing hair positions of the top wing layer. Wings with reduced Myo10A (L, L’) displayed multiple wing hairs, misdirection of patches of hairs (e.g., see L’), and some overall wing folding (not shown). In all panels except D, wings were collected from male flies grown at 29°C. Due to low male viability, wings in D were collected from female flies grown at 29°C. Calibration: 25 µm. (TIF) [file pone.0328970.s001.tif]

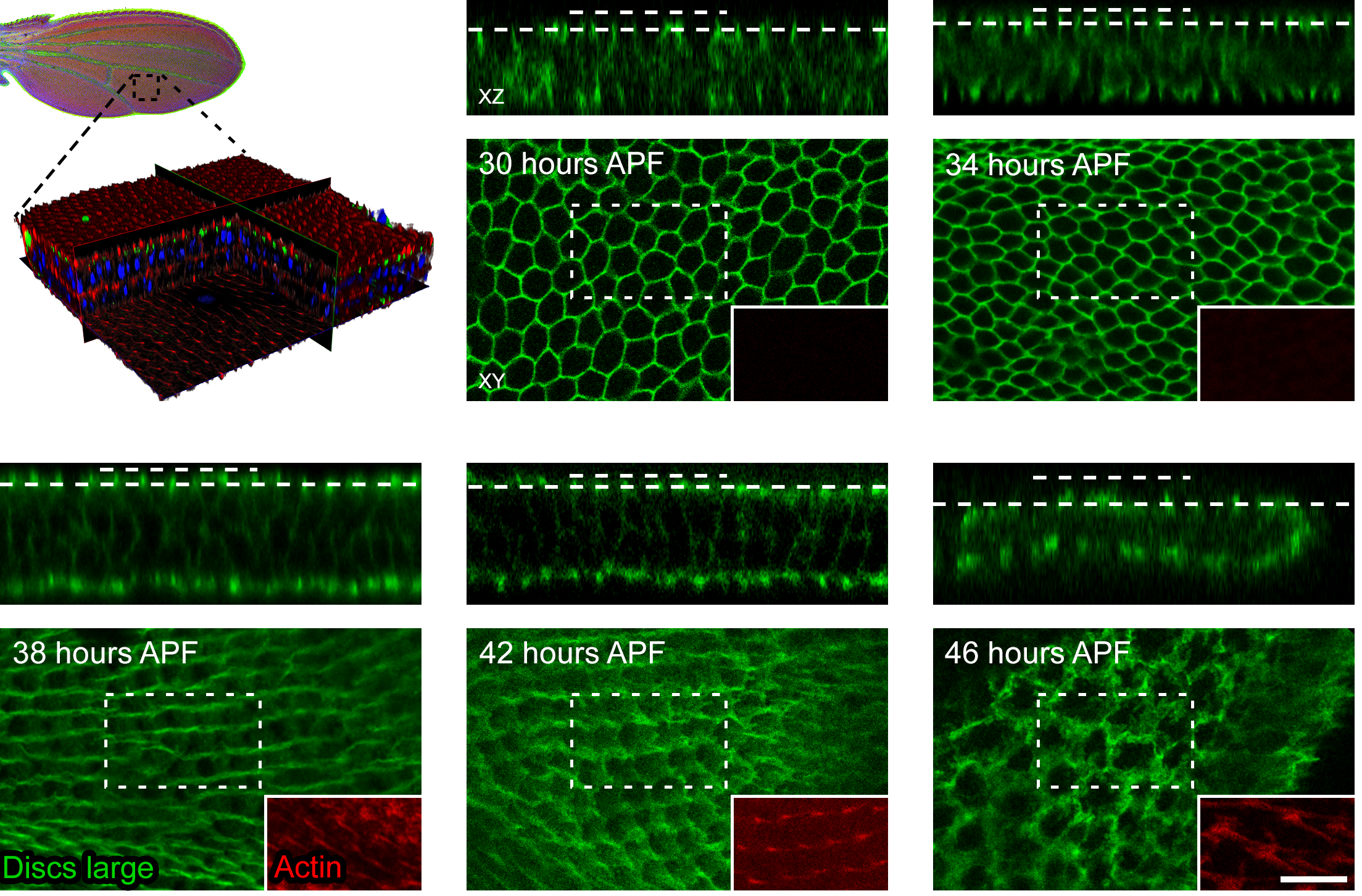

Supplement: S2 Fig — As depicted in the top left panel, select images are apical XY and transverse XZ planes of the wing bilayer. The bilayer is apparent through the two layers of DAPI labeled nuclei (blue). Wing hairs (Phalloidin, red) extend from the apical surface of both the top and bottom cell layers. In developing pupal wings, Dlg (green) is present around the cell periphery at 30 and 34 hours APF. At these time points no hair extension was observed (insets). By 38 hours APF, Dlg relocalizes into continuous apical ribbons and developing hairs (red, inset) extend beyond the apical cell surface. Lower levels of Dlg remain at the cell periphery at the basolateral cell surfaces (see Fig 3B). At 42 hours APF, as the hairs continue to develop, the Dlg ribbons are no longer continuous. By 46 hours APF, Dlg is no longer present in apical ribbons, but is again present only around the cell periphery. Calibration: 10 µm. (TIF) [file pone.0328970.s002.tif]

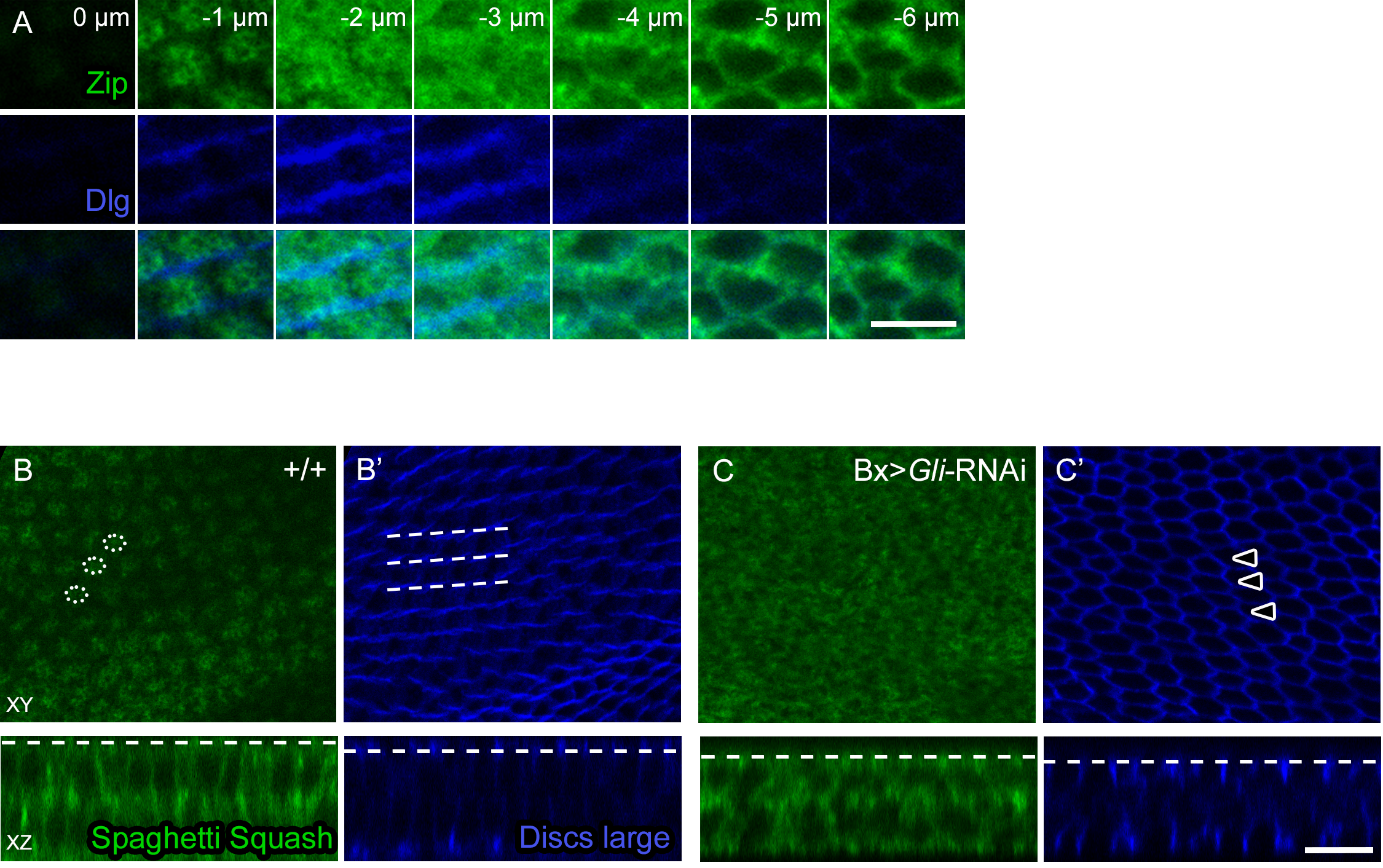

Supplement: S3 Fig — (A) Enlarged XY images of the boxed region in Fig 3D shown at descending 1 µm Z intervals. The 0 µm position is set immediately above the apical cell surface. Zip-GFP (−1 µm) is present in circular accumulations near the apical cell surface. Dlg (−2 to −4 µm) forms ribbons below the apical surface of the wing. Both Zip-GFP and Dlg are present around the cell periphery basolaterally (−5 to −6 µm). Calibration: 5 µm. (B-C) Apical (XY) and transverse (XZ) sections of pupal wings at 38 hours APF. The dotted lines in the XZ images indicate the positions of the XY planes. In Bx-Gal4 driven UAS-lacZ control wings, Dlg (blue) accumulates in apical ribbons at 38 hours APF, with minimal to no Dlg present at proximodistal cell boundaries (B’; dotted parallel lines track three example ribbons). Immediately apical to these ribbons, Sqh-GFP (green) is present in circular accumulations (B; dotted ellipses outline example accumulations). In Bx-Gal4 driven UAS-Gli-RNAi wings, Dlg does not form continuous ribbons, but remains around the lateral cell surface (C’). Arrowheads indicate residual accumulation at the proximodistal cell boundaries. Unlike in wildtype, Sqh does not accumulate apically, but remains diffuse at the apical surface (C). Calibration: 10 µm. (TIF) [file pone.0328970.s003.tif]
