# Supplementary material for: A role for Myo-II zipper and spaghetti squash in Gliotactin-dependent Drosophila melanogaster wing hair planar cell polarity
Source: PLoS One. 2025 Jul 23;20(7):e0328970. doi: 10.1371/journal.pone.0328970 (PMC12286378; doi:10.1371/journal.pone.0328970)
Supplement: S4 File — A summary document of the statistical analyses used to quantify the sqh-RNAi and zip-RNAi hair misalignment phenotypes (as summarized in Table 1). (PDF) [file pone.0328970.s004.pdf]

Supplemental Material 4: RNAi Data Analysis Report

May 30, 2025

This file performs the statistical analyses comparing the percentage of wing hair misalignments (termed "score") for varying temperatures of various genotypes.

Data description

The dataset columns have the following descriptions.

Column Name	Description
`sample_ID`	Unique identifier for each sample
`genotype`	Genotype classification
`temp`	Temperature (18, 22, 25, 29) measured in degrees celsius
`score`	Percentage of misaligned hairs in a 50 hair region

Genotype description

Genotype	Description
`bx-Gal4`	bx-Gal4
`lacZ`	UAS-lacZ
`sqh-RNAi`	UAS-sqh-RNAi
`zip-RNAi`	UAS-zip-RNAi
`nub-Gal4`	nub-Gal4

Load necessary libraries and do setup

```
library(tidyverse)
```

```
## Warning: package 'dplyr' was built under R version 4.3.3
```

```
## -- Attaching core tidyverse packages ------------------------ tidyverse 2.0.0 --
## v dplyr     1.1.4     v readr     2.1.4
## v forcats   1.0.0     v stringr   1.5.0
## v ggplot2   3.5.2     v tibble    3.2.1
## v lubridate 1.9.3     v tidyr     1.3.0
## v purrr     1.0.2
## -- Conflicts ------------------------------------------ tidyverse_conflicts() --
```

```
## x dplyr::filter() masks stats::filter()
## x dplyr::lag()    masks stats::lag()
## i Use the conflicted package (<http://conflicted.r-lib.org/>) to force all conflicts to become error
```

```r
library(readxl)
library(ggplot2)
library(broom)
library(FSA) # Perform Dunn's test with Bonferroni adjustment
```

```
## Warning: package 'FSA' was built under R version 4.3.3

## ## FSA v0.9.5. See citation('FSA') if used in publication.
## ## Run fishR() for related website and fishR('IFAR') for related book.
```

```r
library(latex2exp)
```

```
## Warning: package 'latex2exp' was built under R version 4.3.3
```

Get Bx-Gal4 data ready

Load and clean data

```r
raw_bx_data <- read_excel("data/RNAiscores.xlsx", sheet = 1)

# Reorder and set Genotype factor
raw_bx_data <- raw_bx_data %>%
  mutate(genotype = factor(genotype, levels = c('lacZ', 'sqh-RNAi', 'zip-RNAi')),
         temp = factor(temp))

# Print out summary of the data
str(raw_bx_data)
```

```
## tibble [360 x 4] (S3: tbl_df/tbl/data.frame)
##  $ sample_ID: num [1:360] 1 2 3 4 5 6 7 8 9 10 ...
##  $ genotype : Factor w/ 3 levels "lacZ","sqh-RNAi",..: 1 1 1 1 1 1 1 1 1 1 ...
##  $ temp     : Factor w/ 4 levels "18","22","25",..: 1 1 1 1 1 1 1 1 1 1 ...
##  $ score    : num [1:360] 6 0 0 0 2 2 4 2 0 0 ...
```

```r
head(raw_bx_data)
```

```
## # A tibble: 6 x 4
##   sample_ID genotype temp  score
##       <dbl> <fct>    <fct> <dbl>
## 1         1 lacZ     18        6
## 2         2 lacZ     18        0
## 3         3 lacZ     18        0
## 4         4 lacZ     18        0
## 5         5 lacZ     18        2
## 6         6 lacZ     18        2
```

```
summary(raw_bx_data)
```

```
##    sample_ID          genotype   temp         score
##  Min.   :  1.00   lacZ    :160   18:120   Min.   : 0.000
##  1st Qu.: 90.75   sqh-RNAi: 80   22: 80   1st Qu.: 0.000
##  Median :180.50   zip-RNAi:120   25: 80   Median : 2.000
##  Mean   :180.50                  29: 80   Mean   : 6.028
##  3rd Qu.:270.25                           3rd Qu.:10.000
##  Max.   :360.00                           Max.   :52.000
```

Explore

This section gives some summary statistics and initial visualizations of the dataset.

Means and Standard deviations of Score

```
summarized_bx_data <- raw_bx_data %>%
  group_by(temp, genotype) %>%
  summarize(mean_score = mean(score, na.rm = TRUE),
            sd_score = sd(score, na.rm = TRUE),
            median_score = median(score, na.rm = TRUE))
```

```
## 'summarise()' has grouped output by 'temp'. You can override using the
## '.groups' argument.
```

```
# Print out summary of the data
head(summarized_bx_data, n=10)
```

```
## # A tibble: 9 x 5
## # Groups:   temp [4]
##   temp  genotype mean_score sd_score median_score
##   <fct> <fct>         <dbl>    <dbl>        <dbl>
## 1 18    lacZ           1.25     2.85            0
## 2 18    sqh-RNAi      16.1     11.6            14
## 3 18    zip-RNAi       5.35     5.89            4
## 4 22    lacZ           0.65     1.66            0
## 5 22    sqh-RNAi      16.1      9.28           16
## 6 25    lacZ           0.75     1.96            0
## 7 25    zip-RNAi       5.3      5.33            4
## 8 29    lacZ           0.6      1.58            0
## 9 29    zip-RNAi       8.15     6.54            8
```

Plots of Score distributions

```r
# Custom labels for genotype fill
custom_labels <- c(
  "bx-Gal4" = TeX("bx-Gal4"),
  "lacZ" = TeX("UAS-lacZ"),
  "sqh-RNAi" = TeX("UAS-sqh-RNAi"),
  "zip-RNAi" = TeX("UAS-zip-RNAi"),
  "nub-Gal4" = TeX("nub-Gal4")
)

# Set seed for reproducibility
set.seed(2)

# Plot
p <- ggplot(raw_bx_data, aes(x = temp, y = score, fill = genotype)) +
  geom_jitter(position = position_jitterdodge(jitter.width = 0.2, dodge.width = 0.8),
              alpha = 0.5, shape = 21, color = "black") +  # points with fill
  stat_summary(fun = median, geom = "crossbar", width = 0.7,
               color = "black",
               position = position_dodge(width = 0.8)) +  # median line
  labs(title = "Scores by Temperature and Genotype", x = "Temp", y = "Score", fill = "Genotype") +
  scale_fill_discrete(labels = custom_labels) +
  theme_minimal()
  # theme(legend.position = c(1, 1), legend.justification = c(1.75, 1),
  #       legend.background = element_rect(fill = "white", color = "black", linewidth = 0.1)
  #     )
# Optional: Save the plot
# ggsave("figures/rnai_temp_and_genotype.tiff", plot = p, width = 7, height = 3, dpi = 300)
p
```

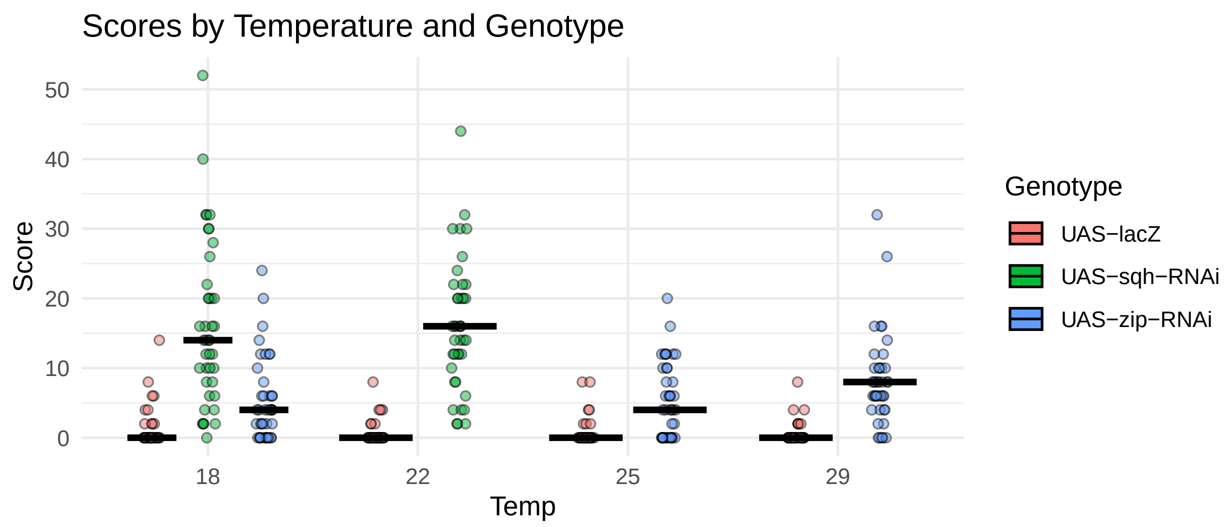


```r
ggplot(raw_bx_data, aes(x = score)) +
  geom_histogram(binwidth = 1, fill = "steelblue", color = "black") +
  facet_grid(temp ~ genotype) +
  labs(title = "Histogram of Scores by Temperature and Genotype",
       x = "Score", y = "Count") +
  theme_minimal()
```

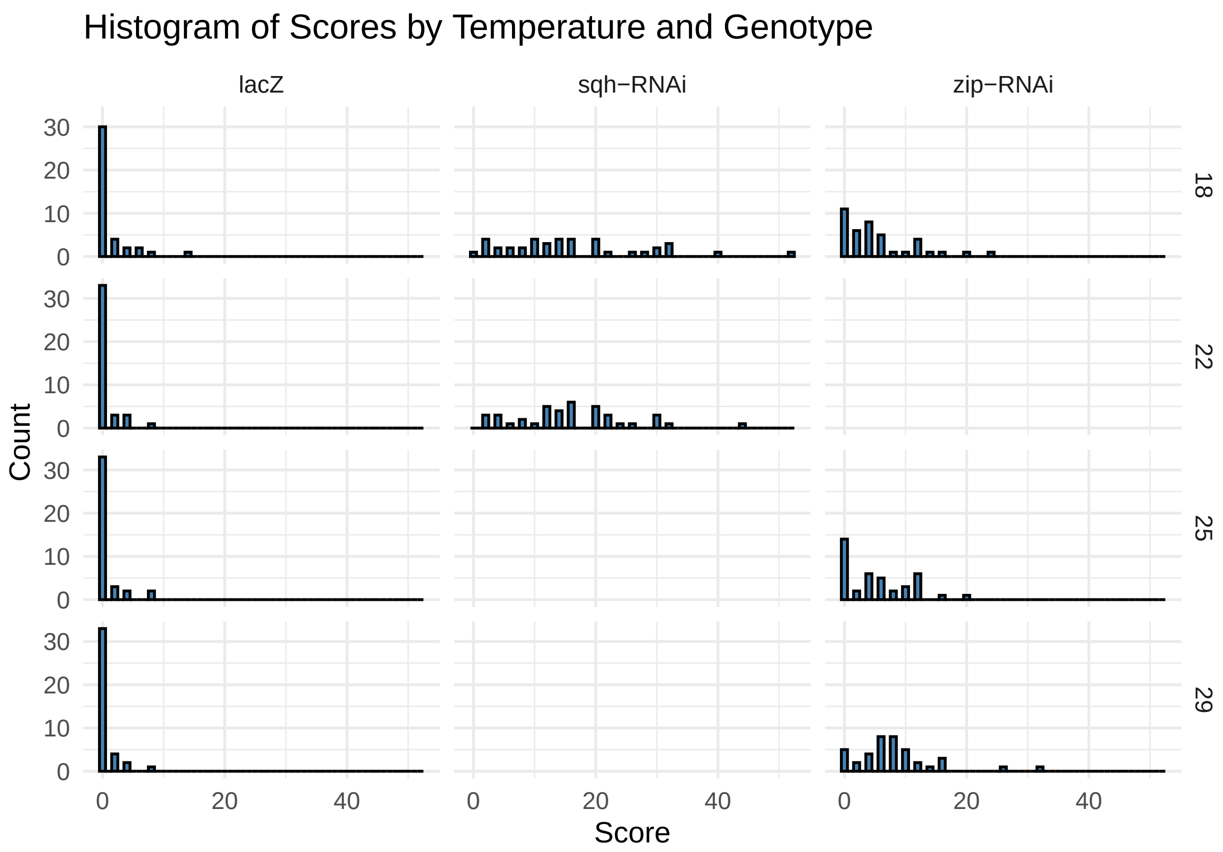

The plot above shows the distribution of the scores by temperature in each genotype category. We investigate if these differences are statistically significant in the next section.

Analysis

ANOVA assumptions are violated (residuals of ANOVA model are not normally distributed). So, we use non-parametric approaches to compare medians.

Non-parametric Methods: 18 temp

For the 18 temp samples, we use the Kruskal-Wallis test to compare medians followed by a post-hoc test for pairwise differences.

```
# Subset data for temp == "18"
data_18temp <- raw_bx_data %>% filter(temp == "18")

# Perform Kruskal-Wallis test
kruskal_result <- kruskal.test(score ~ genotype, data = data_18temp)
kruskal_result
```

```
##
##  Kruskal-Wallis rank sum test
##
```

```
## data:  score by genotype
## Kruskal-Wallis chi-squared = 59.612, df = 2, p-value = 1.136e-13
```

```r
dunn_result <- dunnTest(score ~ genotype, data = data_18temp, method = "bonferroni")
dunn_result
```

```
## Dunn (1964) Kruskal-Wallis multiple comparison

##   p-values adjusted with the Bonferroni method.

##            Comparison         Z      P.unadj        P.adj
## 1     lacZ - sqh-RNAi -7.706867 1.289436e-14 3.868307e-14
## 2     lacZ - zip-RNAi -3.451057 5.583951e-04 1.675185e-03
## 3 sqh-RNAi - zip-RNAi  4.255810 2.082933e-05 6.248800e-05
```

Non-parametric Methods: 22 temp

For the 22 temp samples, we use the Mann-Whitney U test to compare medians.

```r
# Subset only the two temp levels
data_22temp <- raw_bx_data %>% filter(temp == "22")

# Mann-Whitney U test (a.k.a. Wilcoxon rank-sum test)
wilcox_result <- wilcox.test(score ~ genotype, data = data_22temp, exact=FALSE)
wilcox_result
```

```
##
##  Wilcoxon rank sum test with continuity correction
##
## data:  score by genotype
## W = 26, p-value = 1.103e-14
## alternative hypothesis: true location shift is not equal to 0
```

Non-parametric Methods: 25 temp

For the 25 temp samples, we use the Mann-Whitney U test to compare medians.

```r
# Subset only the two temp levels
data_25temp <- raw_bx_data %>% filter(temp == "25")

# Mann-Whitney U test (a.k.a. Wilcoxon rank-sum test)
wilcox_result <- wilcox.test(score ~ genotype, data = data_25temp, exact=FALSE)
wilcox_result
```

```
##
##  Wilcoxon rank sum test with continuity correction
##
## data:  score by genotype
## W = 370, p-value = 3.579e-06
## alternative hypothesis: true location shift is not equal to 0
```

Non-parametric Methods: 29 temp

For the 29 temp samples, we use the Mann-Whitney U test to compare medians.

```
# Subset only the two temp levels
data_29temp <- raw_bx_data %>% filter(temp == "29")

# Mann-Whitney U test (a.k.a. Wilcoxon rank-sum test)
wilcox_result <- wilcox.test(score ~ genotype, data = data_29temp, exact=FALSE)
wilcox_result
```

```
##
##  Wilcoxon rank sum test with continuity correction
##
## data:  score by genotype
## W = 147.5, p-value = 2.879e-11
## alternative hypothesis: true location shift is not equal to 0
```

The non-parametric analyses show that all pairings of genotypes within a given temperature have statistically different median scores.

Get nub-Gal4 data ready

Load and clean data

```
raw_nub_data <- read_excel("data/RNAiscores.xlsx", sheet = 2)

# Reorder and set Genotype factor
raw_nub_data <- raw_nub_data %>%
  mutate(genotype = factor(genotype, levels = c('lacZ', 'sqh-RNAi', 'zip-RNAi')),
         temp = factor(temp))

# Print out summary of the data
str(raw_nub_data)
```

```
## tibble [360 x 4] (S3: tbl_df/tbl/data.frame)
##  $ sample_ID: num [1:360] 1 2 3 4 5 6 7 8 9 10 ...
##  $ genotype : Factor w/ 3 levels "lacZ","sqh-RNAi",..: 1 1 1 1 1 1 1 1 1 1 ...
##  $ temp     : Factor w/ 4 levels "18","22","25",..: 1 1 1 1 1 1 1 1 1 1 ...
##  $ score    : num [1:360] 0 0 0 0 4 0 0 2 0 0 ...
```

```
head(raw_nub_data)
```

```
## # A tibble: 6 x 4
##   sample_ID genotype temp  score
##       <dbl> <fct>    <fct> <dbl>
## 1         1 lacZ     18        0
## 2         2 lacZ     18        0
## 3         3 lacZ     18        0
```

```
## 4          4 lacZ     18        0
## 5          5 lacZ     18        4
## 6          6 lacZ     18        0
```

```
summary(raw_nub_data)
```

```
##    sample_ID          genotype    temp         score
##  Min.   :  1.00   lacZ     :160   18:120   Min.   : 0.000
##  1st Qu.: 90.75   sqh-RNAi: 80    22: 80   1st Qu.: 0.000
##  Median :180.50   zip-RNAi:120    25: 80   Median : 0.000
##  Mean   :180.50                   29: 80   Mean   : 3.272
##  3rd Qu.:270.25                            3rd Qu.: 4.000
##  Max.   :360.00                            Max.   :32.000
```

Explore

This section gives some summary statistics and initial visualizations of the dataset.

Means and Standard deviations of Score

```
summarized_nub_data <- raw_nub_data %>%
  group_by(temp, genotype) %>%
  summarize(mean_score = mean(score, na.rm = TRUE),
            sd_score = sd(score, na.rm = TRUE),
            median_score = median(score, na.rm = TRUE))
```

```
## `summarise()` has grouped output by 'temp'. You can override using the
## `.groups` argument.
```

```
# Print out summary of the data
str(summarized_nub_data)
```

```
## gropd_df [9 x 5] (S3: grouped_df/tbl_df/tbl/data.frame)
##  $ temp        : Factor w/ 4 levels "18","22","25",..: 1 1 1 2 2 3 3 4 4
##  $ genotype    : Factor w/ 3 levels "lacZ","sqh-RNAi",..: 1 2 3 1 2 1 3 1 3
##  $ mean_score  : num [1:9] 1.1 6.45 2.3 0.4 8.05 0.35 4.15 0.75 5.9
##  $ sd_score    : num [1:9] 3.23 5.74 3.02 1.03 6.22 ...
##  $ median_score: num [1:9] 0 4 0 0 8 0 4 0 4
##  - attr(*, "groups")= tibble [4 x 2] (S3: tbl_df/tbl/data.frame)
##   ..$ temp : Factor w/ 4 levels "18","22","25",..: 1 2 3 4
##   ..$ .rows: list<int> [1:4]
##   .. ..$ : int [1:3] 1 2 3
##   .. ..$ : int [1:2] 4 5
##   .. ..$ : int [1:2] 6 7
##   .. ..$ : int [1:2] 8 9
##   .. ..@ ptype: int(0)
##   ..- attr(*, ".drop")= logi TRUE
```

```r
head(summarized_nub_data, n=10)
```

```
## # A tibble: 9 x 5
## # Groups:   temp [4]
##   temp  genotype mean_score sd_score median_score
##   <fct> <fct>         <dbl>    <dbl>        <dbl>
## 1 18    lacZ           1.1      3.23            0
## 2 18    sqh-RNAi       6.45     5.74            4
## 3 18    zip-RNAi       2.3      3.02            0
## 4 22    lacZ           0.4      1.03            0
## 5 22    sqh-RNAi       8.05     6.22            8
## 6 25    lacZ           0.35     1.00            0
## 7 25    zip-RNAi       4.15     3.96            4
## 8 29    lacZ           0.75     2.01            0
## 9 29    zip-RNAi       5.9      6.13            4
```

```r
summary(summarized_nub_data)
```

```
##  temp      genotype   mean_score       sd_score      median_score
##  18:3   lacZ    :4   Min.   :0.350   Min.   :1.001   Min.   :0.000
##  22:2   sqh-RNAi:2   1st Qu.:0.750   1st Qu.:2.010   1st Qu.:0.000
##  25:2   zip-RNAi:3   Median :2.300   Median :3.233   Median :0.000
##  29:2                Mean   :3.272   Mean   :3.593   Mean   :2.222
##                      3rd Qu.:5.900   3rd Qu.:5.738   3rd Qu.:4.000
##                      Max.   :8.050   Max.   :6.218   Max.   :8.000
```

Plots of Score distributions

```r
# Plot
p <- ggplot(raw_nub_data, aes(x = temp, y = score, fill = genotype)) +
  geom_jitter(position = position_jitterdodge(jitter.width = 0.2, dodge.width = 0.8),
              alpha = 0.5, shape = 21, color = "black") +  # points with fill
  stat_summary(fun = median, geom = "crossbar", width = 0.7,
               color = "black",
               position = position_dodge(width = 0.8)) +  # median line
  labs(title = "Scores by Temperature and Genotype", x = "Temp", y = "Score", fill = "Genotype") +
  scale_fill_discrete(labels = custom_labels) +
  theme_minimal()
  # theme(
  #   legend.position = c(1, 1),
  #   legend.justification = c(4, 1),
  #   legend.background = element_rect(fill = "white", color = "black", linewidth = 0.1)
  # )

# Optional: Save the plot
# ggsave("figures/nub_temp_and_genotype.tiff", plot = p, width = 7, height = 3, dpi = 300)

p
```

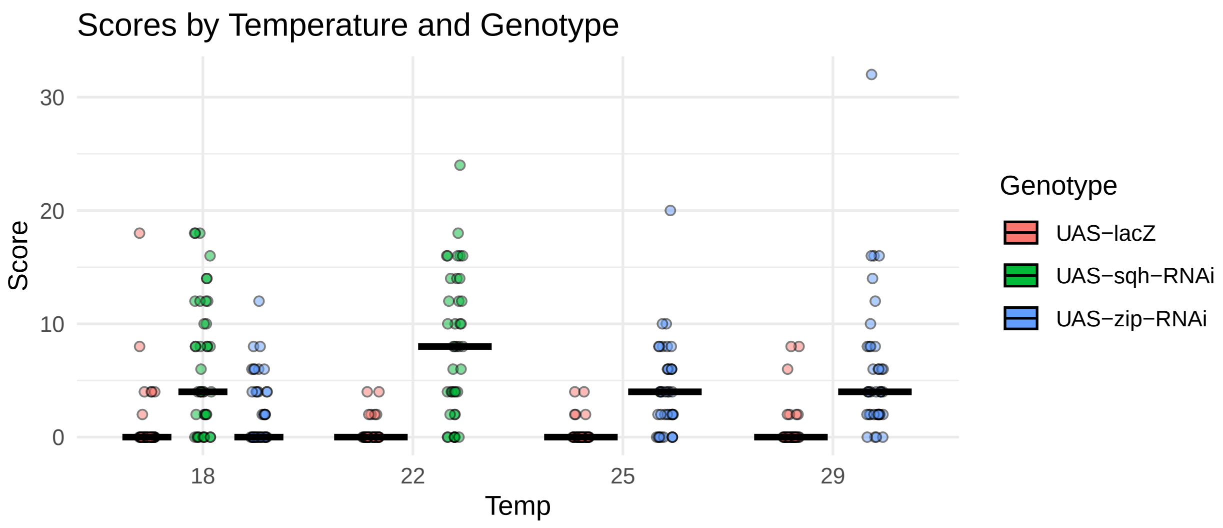


```r
ggplot(raw_nub_data, aes(x = score)) +
  geom_histogram(binwidth = 1, fill = "steelblue", color = "black") +
  facet_grid(temp ~ genotype) +
  labs(title = "Histogram of Scores by Temperature and Genotype",
       x = "Score", y = "Count") +
  theme_minimal()
```

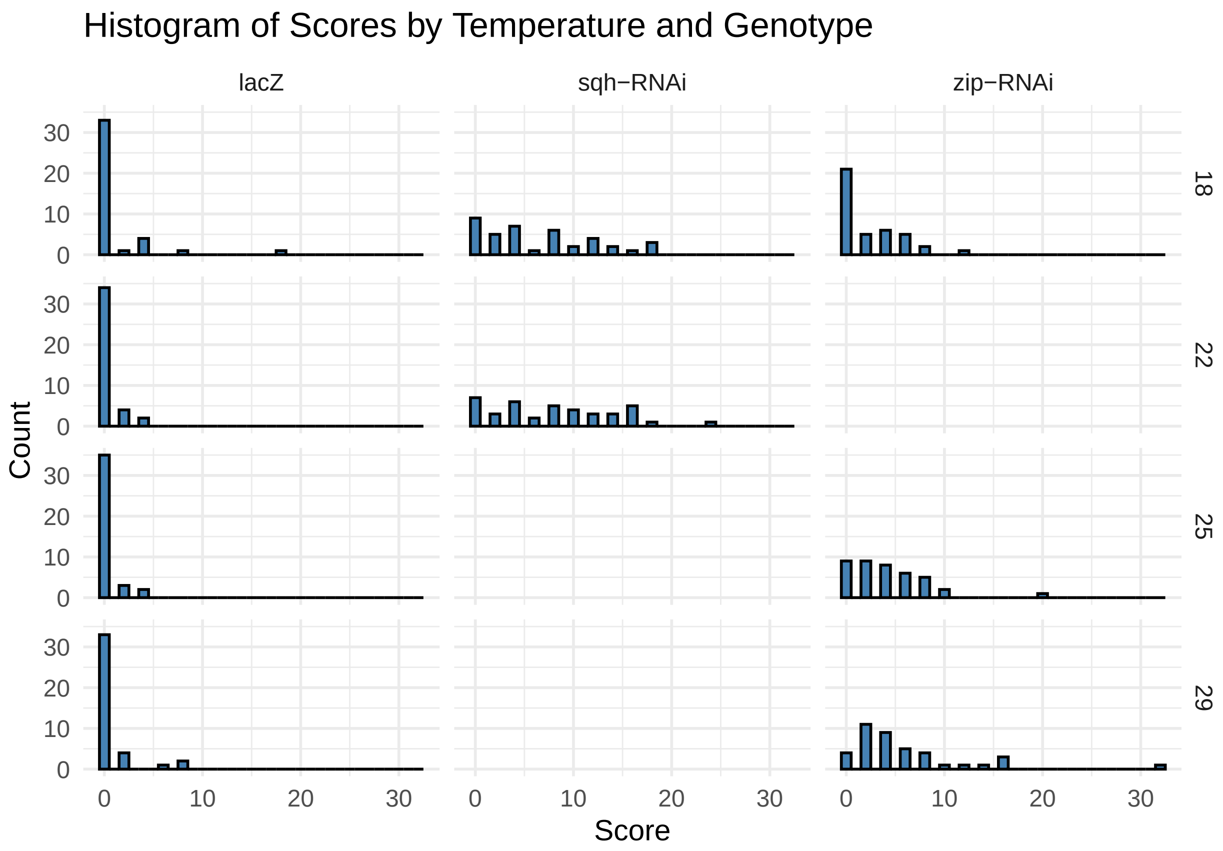

The plot above shows the distribution of the scores by temperature in each genotype category. We investigate if these differences are statistically significant in the next section.

Analysis

ANOVA assumptions are violated (residuals of ANOVA model are not normally distributed). So, we use non-parametric approaches to compare medians.

Non-parametric Methods: 18 temp

For the 18 temp samples, we use the Kruskall-Wallis test to compare medians followed by a post-hoc test for pairwise differences.

```
# Subset data for temp == "18"
data_18temp <- raw_nub_data %>% filter(temp == "18")

# Perform Kruskal-Wallis test
kruskal_result <- kruskal.test(score ~ genotype, data = data_18temp)
kruskal_result
```

```
##
##  Kruskal-Wallis rank sum test
##
## data:  score by genotype
## Kruskal-Wallis chi-squared = 31.674, df = 2, p-value = 1.325e-07
```

```
dunn_result <- dunnTest(score ~ genotype, data = data_18temp, method = "bonferroni")
dunn_result
```

```
## Dunn (1964) Kruskal-Wallis multiple comparison

##   p-values adjusted with the Bonferroni method.

##             Comparison         Z      P.unadj        P.adj
## 1     lacZ - sqh-RNAi -5.583109 2.362572e-08 7.087717e-08
## 2     lacZ - zip-RNAi -2.177499 2.944332e-02 8.832997e-02
## 3 sqh-RNAi - zip-RNAi  3.405609 6.601663e-04 1.980499e-03
```

Non-parametric Methods: 22 temp

For the 22 temp samples, we use the Mann-Whitney U test to compare medians.

```
# Subset only the two temp levels
data_22temp <- raw_nub_data %>% filter(temp == "22")

# Mann-Whitney U test (a.k.a. Wilcoxon rank-sum test)
wilcox_result <- wilcox.test(score ~ genotype, data = data_22temp, exact=FALSE)
wilcox_result
```

```
##
##  Wilcoxon rank sum test with continuity correction
##
## data:  score by genotype
## W = 179, p-value = 1.303e-10
## alternative hypothesis: true location shift is not equal to 0
```

Non-parametric Methods: 25 temp

For the 25 temp samples, we use the Mann-Whitney U test to compare medians.

```
# Subset only the two temp levels
data_25temp <- raw_nub_data %>% filter(temp == "25")

# Mann-Whitney U test (a.k.a. Wilcoxon rank-sum test)
wilcox_result <- wilcox.test(score ~ genotype, data = data_25temp, exact=FALSE)
wilcox_result
```

```
##
##  Wilcoxon rank sum test with continuity correction
##
## data:  score by genotype
## W = 242, p-value = 3.716e-09
## alternative hypothesis: true location shift is not equal to 0
```

Non-parametric Methods: 29 temp

For the 29 temp samples, we use the Mann-Whitney U test to compare medians.

```
# Subset only the two temp levels
data_29temp <- raw_nub_data %>% filter(temp == "29")

# Mann-Whitney U test (a.k.a. Wilcoxon rank-sum test)
wilcox_result <- wilcox.test(score ~ genotype, data = data_29temp, exact=FALSE)
wilcox_result
```

```
##
##  Wilcoxon rank sum test with continuity correction
##
## data:  score by genotype
## W = 192.5, p-value = 6.275e-10
## alternative hypothesis: true location shift is not equal to 0
```

The non-parametric analyses show that all pairings of genotypes within a given temperature have statistically different median scores. The only exception at the 0.05 level of significance is the comparison of `lacZ - zip-RNAi` for 18 temp which had a p-value of about 0.088.
