# Supplementary material for: A role for Myo-II zipper and spaghetti squash in Gliotactin-dependent Drosophila melanogaster wing hair planar cell polarity
Source: PLoS One. 2025 Jul 23;20(7):e0328970. doi: 10.1371/journal.pone.0328970 (PMC12286378; doi:10.1371/journal.pone.0328970)
Supplement: S5 File — A summary document of the statistical analyses used to test for a genetic interaction between Gli and sqh or zip (as summarized in Fig 2M). (PDF) [file pone.0328970.s005.pdf]

Supplemental Material 5: Interaction Data Analysis Report

December 19, 2024

This file reproduces the figures and statistics reported in the manuscript for quantifying the interaction effects observed. As a guide, this table helps map variable names in the code to the biological meanings:

Code Category Name	Code Variable Name	Biological Variable Name
`new_gene`	`zip/+`	$zip^{1}/+$
	`sqh/+`	$sqh^{AX3}/+$
	`+/+`	Gli^{+}/Gli^{+}
	`dv5/+`	$Gli^{dv5}/+$
`dv5_base`	`dv5/RAR`	Gli^{dv5}/Gli^{RAR77}
	`dv5/dv5`	Gli^{dv5}/Gli^{dv5}

Load necessary libraries and do setup

```
library(tidyverse)
```

```
## Warning: package 'dplyr' was built under R version 4.3.3
```

```
## -- Attaching core tidyverse packages ------------------------ tidyverse 2.0.0 --
## v dplyr     1.1.4     v readr     2.1.4
## v forcats   1.0.0     v stringr   1.5.0
## v ggplot2   3.5.2     v tibble    3.2.1
## v lubridate 1.9.3     v tidyr     1.3.0
## v purrr     1.0.2
## -- Conflicts ------------------------------------------ tidyverse_conflicts() --
## x dplyr::filter() masks stats::filter()
## x dplyr::lag()    masks stats::lag()
## i Use the conflicted package (<http://conflicted.r-lib.org/>) to force all conflicts to become errors
```

```
library(readxl)
library(ggplot2)
library(FSA)
```

```
## Warning: package 'FSA' was built under R version 4.3.3
```

```
## ## FSA v0.9.5. See citation('FSA') if used in publication.
## ## Run fishR() for related website and fishR('IFAR') for related book.
```

```r
library(latex2exp)
```

```
## Warning: package 'latex2exp' was built under R version 4.3.3
```

```r
# Set bootstrap samples
NUM_BOOTSTRAPS = 10000
```

Get data ready

Load and clean data

```r
raw_data <- read_excel("data/interactionscores.xlsx",
    col_types = c("skip", "numeric", "numeric",
                  "skip", "numeric", "numeric", "numeric",
                  "skip", "numeric", "numeric", "numeric",
                  "skip", "numeric", "numeric", "numeric"))

# Select the first 90 rows
WingScores_by_cols <- head(raw_data, 90)

str(WingScores_by_cols)
```

```
## tibble [90 x 11] (S3: tbl_df/tbl/data.frame)
##  $ +/+ & zip/+    : num [1:90] 0 0 0 0 1 0 1 1 1 0 ...
##  $ +/+ & sqh/+    : num [1:90] 0 0 0 0 0 0 0 0 0 0 ...
##  $ dv5/+ & +/+    : num [1:90] 0 0 0 0 1 0 2 0 1 1 ...
##  $ dv5/+ & zip/+  : num [1:90] 0 0 1 0 1 1 2 0 0 2 ...
##  $ dv5/+ & sqh/+  : num [1:90] 1 0 1 1 0 2 1 0 0 0 ...
##  $ dv5/RAR & +/+  : num [1:90] 5 3 3 5 4 5 4 3 2 3 ...
##  $ dv5/RAR & zip/+: num [1:90] 6 3 4 3 3 5 2 2 4 4 ...
##  $ dv5/RAR & sqh/+: num [1:90] 2 5 2 4 6 6 5 5 5 6 ...
##  $ dv5/dv5 & +/+  : num [1:90] 6 4 5 4 2 6 4 4 4 4 ...
##  $ dv5/dv5 & zip/+: num [1:90] 5 5 5 6 4 4 4 7 6 5 ...
##  $ dv5/dv5 & sqh/+: num [1:90] 4 4 7 6 6 5 3 4 6 4 ...
```

```r
head(WingScores_by_cols)
```

```
## # A tibble: 6 x 11
##   `+/+ & zip/+` `+/+ & sqh/+` `dv5/+ & +/+` `dv5/+ & zip/+` `dv5/+ & sqh/+`
##           <dbl>         <dbl>         <dbl>           <dbl>           <dbl>
## 1             0             0             0               0               1
## 2             0             0             0               0               0
## 3             0             0             0               1               1
## 4             0             0             0               0               1
## 5             1             0             1               1               0
## 6             0             0             0               1               2
## # i 6 more variables: `dv5/RAR & +/+` <dbl>, `dv5/RAR & zip/+` <dbl>,
## #   `dv5/RAR & sqh/+` <dbl>, `dv5/dv5 & +/+` <dbl>, `dv5/dv5 & zip/+` <dbl>,
## #   `dv5/dv5 & sqh/+` <dbl>
```

Pivot data into long format

```
WingScores_long <- WingScores_by_cols %>%
  pivot_longer(
    cols = everything(),          # Specify all columns to be pivoted
    names_to = "mutation",        # Name of the new column for variable names
    values_to = "score"           # Name of the new column for scores
  ) %>%
  drop_na()

dv5_levels <- c("+/+", "dv5/+", "dv5/RAR", "dv5/dv5")
WingScores <- WingScores_long %>%
  separate(mutation, into = c("dv5_base", "new_gene"), sep = "&", convert = TRUE) %>%
  mutate(dv5_base = trimws(dv5_base),
         new_gene = trimws(new_gene),
         dv5_base = factor(dv5_base, levels = dv5_levels, ordered = TRUE),
         new_gene = as.factor(new_gene))

str(WingScores)
```

```
## tibble [656 x 3] (S3: tbl_df/tbl/data.frame)
##  $ dv5_base: Ord.factor w/ 4 levels "+/+"<"dv5/+"<..: 1 1 2 2 2 3 3 3 4 4 ...
##  $ new_gene: Factor w/ 3 levels "+/+","sqh/+",..: 3 2 1 3 2 1 3 2 1 3 ...
##  $ score   : num [1:656] 0 0 0 0 1 5 6 2 6 5 ...
```

```
head(WingScores)
```

```
## # A tibble: 6 x 3
##   dv5_base new_gene score
##   <ord>    <fct>    <dbl>
## 1 +/+      zip/+        0
## 2 +/+      sqh/+        0
## 3 dv5/+    +/+          0
## 4 dv5/+    zip/+        0
## 5 dv5/+    sqh/+        1
## 6 dv5/RAR  +/+          5
```

```
summary(WingScores)
```

```
##     dv5_base    new_gene       score      
##  +/+    :180   +/+  :269   Min.   :0.000  
##  dv5/+  :177   sqh/+:191   1st Qu.:0.000  
##  dv5/RAR:164   zip/+:196   Median :1.000  
##  dv5/dv5:135               Mean   :1.805  
##                            3rd Qu.:4.000  
##                            Max.   :7.000
```

Explore

This section gives some summary statistics and initial visualizations of the dataset.

Counts of scores (how many samples for each crossed category)

```
count_table <- WingScores %>%
  count(dv5_base, new_gene)

# Convert the count table into a two-way table
counts_two_way_table <- count_table %>%
  pivot_wider(names_from = new_gene, values_from = n)

rownames(counts_two_way_table) <- NULL
counts_two_way_table <- counts_two_way_table %>% column_to_rownames(var = "dv5_base")

print(counts_two_way_table)
```

```
##          sqh/+ zip/+ +/+
## +/+         90    90  NA
## dv5/+       43    44  90
## dv5/RAR     40    34  90
## dv5/dv5     18    28  89
```

Medians of score

```
# Summarize the data to get the median score for each combination of dv5_base and new_gene
median_scores <- WingScores %>%
  group_by(dv5_base, new_gene) %>%
  summarize(median_score = median(score, na.rm = FALSE))
```

```
## `summarise()` has grouped output by 'dv5_base'. You can override using the
## `.groups` argument.
```

```
med_summary_table <- xtabs(median_score ~ dv5_base + new_gene, data = median_scores)
med_summary_table['+/+', '+/+'] <- NA
print(med_summary_table)
```

```
##           new_gene
## dv5_base  +/+ sqh/+ zip/+
##   +/+           0     0
##   dv5/+     0     0     0
##   dv5/RAR   3     5     4
##   dv5/dv5   4     5     5
```

Means of score

```r
# Summarize the data to get the mean score for each combination of dv5_base and new_gene
mean_scores <- WingScores %>%
  group_by(dv5_base, new_gene) %>%
  summarize(mean_score = mean(score, na.rm = TRUE))
```

```
## `summarise()` has grouped output by 'dv5_base'. You can override using the
## `.groups` argument.
```

```r
mean_summary_table <- xtabs(mean_score ~ dv5_base + new_gene, data = mean_scores)
mean_summary_table['+/+', '+/+'] <- NA
print(mean_summary_table)
```

```
##          new_gene
## dv5_base         +/+       sqh/+      zip/+
##   +/+                 0.03333333 0.22222222
##   dv5/+   0.16666667 0.51162791 0.47727273
##   dv5/RAR 2.61111111 4.40000000 3.97058824
##   dv5/dv5 3.69662921 4.66666667 5.14285714
```

Plots of score distributions

The histograms show the skewness of the data and indicate issues of normality of the distribution of scores. Since the data is measured on an ordinal scale with 7 possible values and does not satisfy the conditions of normality necessary to conduct ANOVA, we opt for focusing on the medians and using non-parametric test alternatives for assessing significance.

The dashed lines in the histograms are the medians for the respective distribution.

```r
median_scores <- WingScores %>%
  group_by(dv5_base, new_gene) %>%
  summarize(median_score = median(score, na.rm = TRUE))
```

```
## `summarise()` has grouped output by 'dv5_base'. You can override using the
## `.groups` argument.
```

```r
# Histogram with two factors
ggplot(WingScores, aes(x = score, fill = dv5_base)) +
  geom_histogram(binwidth = 1, alpha = 0.7, position = "dodge") +
  facet_wrap(dv5_base ~ new_gene, nrow = 4, ncol = 3) +
  # theme_minimal() +
  labs(title = "Histogram of Scores by new_gene, Faceted by dv5_base",
       x = "Score",
       y = "Count") +
  scale_fill_discrete(name = "dv5_base") +
  geom_vline(data = median_scores, aes(xintercept = median_score),
             color = "blue", linetype = "dashed", linewidth = 1)
```

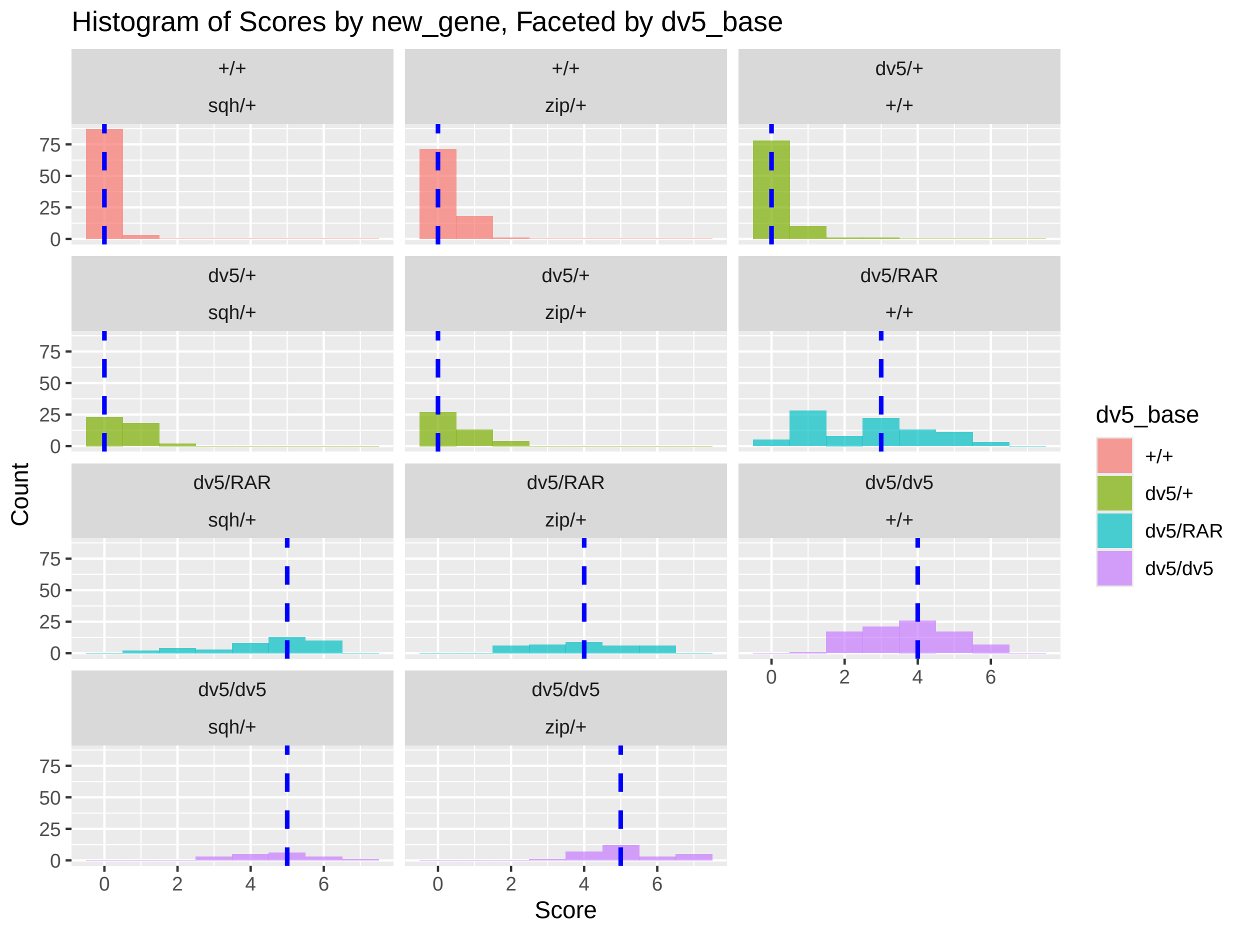

These boxplots clearly show differences in medians between levels within the same category. They also show a spread of scores in each category. The means on these plots are marked with an “X”.

```
# Combine group and level into a single variable for x-axis
WingScores$x_combined <- paste(WingScores$dv5_base,
                                         WingScores$new_gene, sep = ";")

# Make labels for the boxplot x-axis
custom_labels <- c(
  "+/+;sqh/+"=TeX("$\\textit{sqh}^{AX3}/+$"),
  "+/+;zip/+"=TeX("$\\textit{zip}^1/+$"),
  "dv5/+;+/+"=TeX("$\\textit{Gli}^{dv5}/+$"),
  "dv5/+;sqh/+"=TeX("$\\textit{sqh}^{AX3}/+$ ; $\\textit{Gli}^{dv5}/+$"),
  "dv5/+;zip/+"=TeX("$ \\textit{zip}^1$ , $\\textit{Gli}^{dv5}/+$"),
  "dv5/dv5;+/+"=TeX("$\\textit{Gli}^{dv5}/\\textit{Gli}^{dv5}$"),
  "dv5/dv5;sqh/+"=TeX("$\\textit{sqh}^{AX3}/+$ ; $\\textit{Gli}^{dv5}/\\textit{Gli}^{dv5}$"),
  "dv5/dv5;zip/+"=TeX("$\\textit{zip}^1$ , $ \\textit{Gli}^{dv5}/\\textit{Gli}^{dv5}$"),
  "dv5/RAR;+/+"=TeX("$\\textit{Gli}^{dv5}/\\textit{Gli}^{RAR77}$"),
  "dv5/RAR;sqh/+"=TeX("$\\textit{sqh}^{AX3}/+$ ; $\\textit{Gli}^{dv5}/\\textit{Gli}^{RAR77}$"),
  "dv5/RAR;zip/+"=TeX("$\\textit{zip}^1$ , $\\textit{Gli}^{dv5}/\\textit{Gli}^{RAR77}$")
  )

# Boxplot
p <- ggplot(WingScores, aes(x = x_combined, y = score, fill = new_gene)) +
```

```
  geom_boxplot(position = position_dodge(width = 1.0), width = 0.8) +
  stat_summary(fun = mean, geom = "point", shape = 4, size = 3, color = "black",
               position = position_dodge(width = 1.0)) +
  facet_wrap(~ dv5_base, scales = "free_x", nrow=1) +
  scale_fill_manual(values = c("white","lightgrey","darkgrey")) +
  scale_x_discrete(labels = custom_labels) +
  labs(title = "Side-by-Side Box Plots", x = NULL,
       y = "Hair alignment score", fill = NULL) +
  theme_minimal() +
  theme(axis.text.x = element_text(angle = 60, hjust = 1)) +
  guides(fill=FALSE) +
  scale_y_continuous(
  breaks = seq(0, 7, by = 1), # Setting breaks for every unit
  labels = seq(0, 7, by = 1),  # Corresponding labels for the breaks
  minor_breaks = NULL
  )
```

```
## Warning: The `<scale>` argument of `guides()` cannot be `FALSE`. Use "none" instead as
## of ggplot2 3.3.4.
## This warning is displayed once every 8 hours.
## Call `lifecycle::last_lifecycle_warnings()` to see where this warning was
## generated.
```

```
# Save the plot
# ggsave("figures/hair_scores_boxplots_scaled.tiff", plot = p, width = 10, height = 5, dpi = 300)
p
```

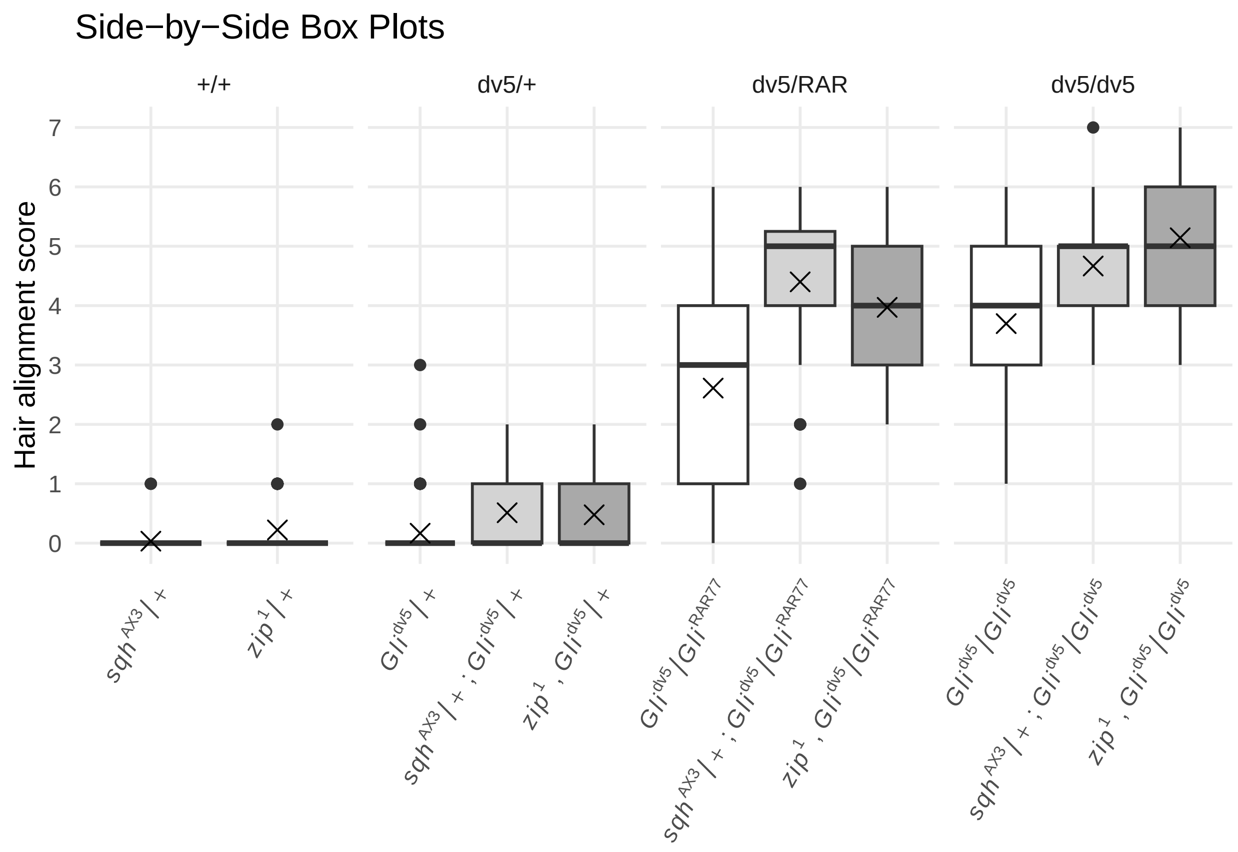

Analysis

Through investigation, we found that trying to use two-way ANOVA with interaction leads to failure of the assumptions of normality of residuals and homogeneity of variance of residuals. Thus, we have to resort to non-parametric methods.

Kruskal-Wallis Test

A non-parametric test to see if there is a significant difference in the distributions of scores across the 3 levels of the `new_gene` factor for each level of the `dv5_base` factor. The following code and output shows that for each of the 4 levels of Gli, there is a significant difference in the distributions of the scores between the 3 `new_gene` levels.

```
# Filter data for $Gli^+/Gli^+$
gli0_level_data <- WingScores %>% filter(dv5_base == '+/+')
kruskal_test <- kruskal.test(score ~ new_gene, data = gli0_level_data)
print(kruskal_test)
```

```
##
##  Kruskal-Wallis rank sum test
##
## data:  score by new_gene
## Kruskal-Wallis chi-squared = 13.228, df = 1, p-value = 0.0002758
```

```r
# Filter data for dv5/+
gli1_level_data <- WingScores %>% filter(dv5_base == 'dv5/+')
kruskal_test <- kruskal.test(score ~ new_gene, data = gli1_level_data)
print(kruskal_test)
```

```
##
##  Kruskal-Wallis rank sum test
##
## data:  score by new_gene
## Kruskal-Wallis chi-squared = 18.756, df = 2, p-value = 8.458e-05
```

```r
# Filter data for dv5/RAR
gli2_level_data <- WingScores %>% filter(dv5_base == 'dv5/RAR')
kruskal_test <- kruskal.test(score ~ new_gene, data = gli2_level_data)
print(kruskal_test)
```

```
##
##  Kruskal-Wallis rank sum test
##
## data:  score by new_gene
## Kruskal-Wallis chi-squared = 35.569, df = 2, p-value = 1.889e-08
```

```r
# Filter data for dv5/dv5
gli3_level_data <- WingScores %>% filter(dv5_base == 'dv5/dv5')
kruskal_test <- kruskal.test(score ~ new_gene, data = gli3_level_data)
print(kruskal_test)
```

```
##
##  Kruskal-Wallis rank sum test
##
## data:  score by new_gene
## Kruskal-Wallis chi-squared = 26.71, df = 2, p-value = 1.585e-06
```

Dunn's test for pairwise comparisons (post-hoc analyses)

The Dunn's test adjusted p-values are the ones we report in the manuscript (annotated on the boxplot). Below, we check within each `dv5_base` category which pairs show significant differences in their medians using Dunn's test. The values we use are the ones in the column labeled `P.adj`.

When `dv5_base = +/+`

```r
dunnTest(score ~ new_gene, data=gli0_level_data, method="bonferroni")
```

```
## Dunn (1964) Kruskal-Wallis multiple comparison

##   p-values adjusted with the Bonferroni method.

##      Comparison         Z      P.unadj        P.adj
## 1 sqh/+ - zip/+ -3.637058 0.0002757703 0.0002757703
```

For Gli^{+}/Gli^{+}, there is no significant difference in the medians of the scores when the `new_gene` is `+/+` or when it is `zip/+`. However, the other two pairs show a significant difference in median scores.

When dv5_base = dv5/+

```
dunnTest(score ~ new_gene, data=gli1_level_data, method="bonferroni")
```

```
## Dunn (1964) Kruskal-Wallis multiple comparison

##   p-values adjusted with the Bonferroni method.

##      Comparison          Z      P.unadj       P.adj
## 1   +/+ - sqh/+ -3.8536106 0.0001163887 0.000349166
## 2   +/+ - zip/+ -3.1239660 0.0017843108 0.005352932
## 3 sqh/+ - zip/+  0.6516349 0.5146367289 1.000000000
```

For the `dv5/+` Gli level, there is no significant difference in the medians of the scores when the `new_gene` is `sqh/+` or when it is `zip/+`. However, the other two pairs show a significant difference in median scores.

When dv5_base = dv5/RAR

```
dunnTest(score ~ new_gene, data=gli2_level_data, method="bonferroni")
```

```
## Dunn (1964) Kruskal-Wallis multiple comparison

##   p-values adjusted with the Bonferroni method.

##      Comparison         Z      P.unadj        P.adj
## 1   +/+ - sqh/+ -5.490198 4.014842e-08 1.204453e-07
## 2   +/+ - zip/+ -3.823784 1.314191e-04 3.942574e-04
## 3 sqh/+ - zip/+  1.172748 2.408968e-01 7.226903e-01
```

For the `dv5/RAR` Gli level, there is no significant difference in the medians of the scores when the `new_gene` is `sqh/+` or when it is `zip/+`. However, the other two pairs show a significant difference in median scores.

When dv5_base = dv5/dv5

```
dunnTest(score ~ new_gene, data=gli3_level_data, method="bonferroni")
```

```
## Dunn (1964) Kruskal-Wallis multiple comparison

##   p-values adjusted with the Bonferroni method.

##      Comparison         Z      P.unadj        P.adj
## 1   +/+ - sqh/+ -2.770320 5.600117e-03 1.680035e-02
## 2   +/+ - zip/+ -4.830048 1.365001e-06 4.095003e-06
## 3 sqh/+ - zip/+ -1.094347 2.738030e-01 8.214091e-01
```

For the `dv5/dv5` Gli level, there is no significant difference in the medians of the scores when the `new_gene` is `sqh/+` or when it is `zip/+`. However, the other two pairs show a significant difference in median scores.

Bootstrapping for finding the signficance of the interactions

The strategy is described as follows:

1. Generate a stratified bootstrap sample for each of the `dv5_base` levels with no `new_gene` mutation.
2. Generate a stratified bootstrap sample for each of the `new_gene` mutations (`sqh/+` and `zip/+`) where `dv5_base = +/+`.
3. Add the response values for each cross of the `new_gene` and `dv5_base` levels to get the expected additive value. Only compute as many added values as there were in the original dataset. So, for example, the original dataset had 18 values for `dv5/dv5` with `sqh/+` so only 18 rows will be computed in the bootstrap sample.
4. Find the median of each crossed-level.
5. Repeat this procedure 10,000 times to generate a null distribution.
6. Calculate a p-value as the number of bootstrap samples with median values greater than or equal to the observed value of the crossed levels of the original data. There will be 6 p-values to compute since there are 3 levels of interest of the `dv5_base` and 2 levels of interest of the `new_gene`.

The analysis in this section helps to establish how significant is the more-than-additive effect we see of combining, for example, `sqh/+` with `dv5/RAR` (resulting in a score increase of 2 when 0 is expected).

Function to calculate the expected medians of combining base levels from each factor

```
# Define the bootstrapping function
bootstrap_expected <- function(data, counts_two_way_table, n_bootstrap = NUM_BOOTSTRAPS) {
  set.seed(123) # For reproducibility
  boot_expected <- replicate(n_bootstrap, {

    # Resample the data stratified
    resample_data <- data %>%
      group_by(dv5_base, new_gene) %>%
      slice_sample(prop = 1, replace = TRUE) %>%
      filter((dv5_base == "+/+" & new_gene != "+/+") |
              (dv5_base != "+/+" & new_gene == "+/+")) %>%
      ungroup()

    # Calculate the expected additive effects
    # Loop over the vectors and filter data
    gli_vals <- c("dv5/+", "dv5/RAR", "dv5/dv5")
    new_gene_vals <- c("sqh/+", "zip/+")

    expected_data <- data.frame()
    for (i in gli_vals) {
      for (j in new_gene_vals) {
        # Filter the data by corresponding values in gli_vals and new_gene_vals
        gli_only <- resample_data %>%
          filter(dv5_base == i, new_gene == '+/+')

        new_gene_only <- resample_data %>%
          filter(dv5_base == '+/+', new_gene == j)

        # Only add as many values together as there were in the original dataset for the crossed levels
        num_to_compute <- counts_two_way_table[i, j]
```

```
        sum <- head(gli_only, num_to_compute)$score + head(new_gene_only, num_to_compute)$score
        expected <- data.frame(dv5_base = rep(NA, num_to_compute),
                              new_gene = rep(NA, num_to_compute),
                              score = rep(NA, num_to_compute))
        expected$score <- sum
        expected$dv5_base <- i
        expected$new_gene <- j
        expected_data <- bind_rows(expected_data, expected)
      }
    }
    resample_data <- bind_rows(resample_data, expected_data)

    # Compute average for the resampled data
    resample_medians <- resample_data %>%
      group_by(dv5_base, new_gene) %>%
      summarize(average_response = median(score), .groups = 'drop') %>%
      filter((dv5_base != "+/+" & new_gene != "+/+"))

    # Combine expected into a vector
    boot_expected <- resample_medians$average_response
  })
  return(boot_expected)
}

# Perform the bootstrapping
boot_results <- bootstrap_expected(WingScores, counts_two_way_table, n_bootstrap = NUM_BOOTSTRAPS)

# Convert bootstrapped differences to a long format data frame for ggplot
boot_expected_long <- as.data.frame(t(boot_results)) %>%
  pivot_longer(cols = everything(), names_to = "Comparison", values_to = "Expected")

# Create labels for the comparisons
comparison_labels <- c("gli1_sqh_expected", "gli1_zip_expected",
                       "gli2_sqh_expected", "gli2_zip_expected",
                       "gli3_sqh_expected", "gli3_zip_expected"
  )

# Add labels to the data frame
boot_expected_long <- boot_expected_long %>%
  mutate(Comparison = factor(Comparison, labels = comparison_labels))
```

Visualize

The plot below shows the distribution of the expected medians of the crossed levels of various bootstrap samples. The dashed red lines in each subplot indicate the observed median score for the respective combination of `dv5_base` and `new_gene` from the original data.

```
# Save the true averages in a dataframe
true_vals <- data.frame(Comparison = comparison_labels,
                        true_scores = as.vector(t(med_summary_table[2:4, 2:3])))

# Histogram of bootstrapped differences
ggplot(boot_expected_long, aes(x = Expected, fill = Comparison)) +
```

```r
  geom_histogram(binwidth = 0.5, alpha = 0.7, position = 'identity') +
  geom_vline(data = true_vals, aes(xintercept = true_scores),
             color = "red", linetype = "dashed", size = 1) +
  facet_wrap(~ Comparison, scales = "free",  nrow = 4, ncol = 2) +
  theme_minimal() +
  labs(title = "Distribution of Bootstrapped Medians",
       x = "Expected Medians",
       y = "Frequency")
```

```
## Warning: Using ‘size‘ aesthetic for lines was deprecated in ggplot2 3.4.0.
## i Please use ‘linewidth‘ instead.
## This warning is displayed once every 8 hours.
## Call ‘lifecycle::last_lifecycle_warnings()‘ to see where this warning was
## generated.
```

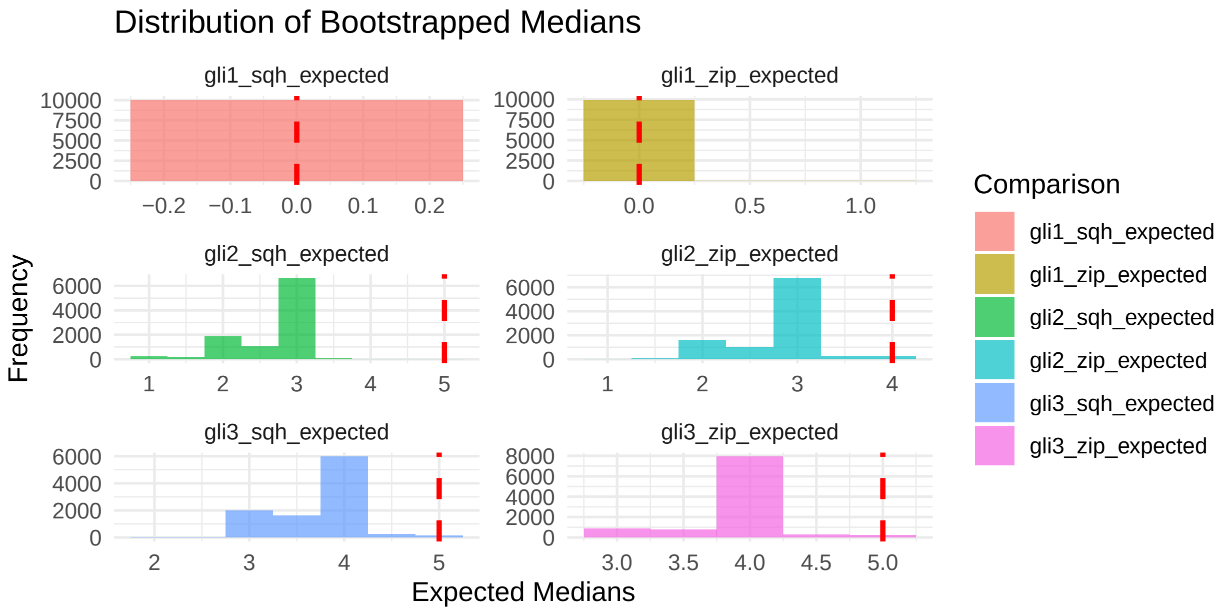

To compute p-values for the interaction, we compute the proportion of interaction values that were greater than or equal to the true (observed) median score value for the respective combination of `dv5_base` and `new_gene`. The p-values can be interpreted in the standard sense: the probability of obtaining this evidence by chance against the null hypothesis. The null hypothesis is that the effect of the `new_gene` (mutation) and `dv5_base` (Gli) is additive. Thus, a low p-value suggests that our evidence is strong against the null hypothesis and the effects we see in the respective combination of Gli and mutation is more than additive.

```r
merged_expected_true <- merge(true_vals, boot_expected_long, by = 'Comparison')

results <- merged_expected_true %>%
  group_by(Comparison) %>%
  summarise(num_extreme = sum(Expected >= true_scores),
            prop_extreme = mean(Expected >= true_scores))

print(results)
```

```
## # A tibble: 6 x 3
```

```
##   Comparison        num_extreme prop_extreme
##   <chr>                   <int>        <dbl>
## 1 gli1_sqh_expected       10000       1
## 2 gli1_zip_expected       10000       1
## 3 gli2_sqh_expected           0       0
## 4 gli2_zip_expected         254       0.0254
## 5 gli3_sqh_expected         142       0.0142
## 6 gli3_zip_expected         212       0.0212
```

To read the table above, consider the row with "Comparison"=`gli3_zip_expected`. The `num_extreme` =212, which tells how many of the bootstrap samples had a median score equal to or greater than the true value for that group from the original data.

The p-values for the significance of this result is given in the column titled `prop_extreme`.
