# Supplementary material for: A role for Myo-II zipper and spaghetti squash in Gliotactin-dependent Drosophila melanogaster wing hair planar cell polarity
Source: PLoS One. 2025 Jul 23;20(7):e0328970. doi: 10.1371/journal.pone.0328970 (PMC12286378; doi:10.1371/journal.pone.0328970)
Supplement: S6 File — A summary document of the statistical analyses used to quantify Dlg localization (as summarized in Fig 4E). (PDF) [file pone.0328970.s006.pdf]

Supplemental Material 6: Dlg Data Analysis Report

June 2, 2025

This file performs the statistical analyses on the Dlg pixel intensity of the anterior-posterior and proximodistal cell boundaries for various genotypes. In this document, we group by fly number to compute one `Average_AP_PD` value for the fly for each genotype (termed "score").

Data description

The dataset columns have the following descriptions.

Column Name	Description
`Fly_number`	Unique identifier for each fly
`Genotype`	Genotype classification
`Cell_number`	Cell number within each fly (5 cells per fly)
`Anterior`	Anterior cell boundary average pixel intensity value
`Proximodistal1`	First proximodistal cell boundary average pixel intensity value
`A/PD1`	Anterior divided by proximodistal measurement (1st)
`Posterior`	Posterior cell boundary average pixel intensity value
`Proximodistal 2`	Second proximodistal cell boundary average pixel intensity value
`P/PD2`	Posterior divided by proximodistal measurement (2nd)
`Average_AP_PD`	Average of A/PD1 and P/PD2 (single value for each cell)

The key columns are the `Genotype` and the `Average_AP_PD`. The `Average_AP_PD` is a single value for each cell that signifies the ratio of the pixel intensity of the anterior-posterior cell boundaries relative to proximodistal cell boundaries.

Genotype description

Genotype	Description
`+_+`	wildtype
`dv5_dv5`	Gli^{dv5}/Gli^{dv5}
`BxGal4_+`	bx-Gal4>lacZ
`BxGal4_GliRNAi`	bx-Gal4>Gli-RNAi

Load necessary libraries and do setup

```
library(tidyverse)
```

```
## Warning: package 'dplyr' was built under R version 4.3.3
```

```
## -- Attaching core tidyverse packages ------------------------ tidyverse 2.0.0 --
## v dplyr     1.1.4     v readr     2.1.4
## v forcats   1.0.0     v stringr   1.5.0
## v ggplot2   3.5.2     v tibble    3.2.1
## v lubridate 1.9.3     v tidyr     1.3.0
## v purrr     1.0.2
## -- Conflicts ------------------------------------------ tidyverse_conflicts() --
## x dplyr::filter() masks stats::filter()
## x dplyr::lag()    masks stats::lag()
## i Use the conflicted package (<http://conflicted.r-lib.org/>) to force all conflicts to become error
```

```
library(readxl)
library(ggplot2)
library(broom)
library(latex2exp)
```

```
## Warning: package 'latex2exp' was built under R version 4.3.3
```

Get data ready

Load and clean data

```
raw_dlg_quant_data <- read_excel("data/dlg_quantification.xlsx")

# Reorder and set Genotype factor
raw_dlg_quant_data <- raw_dlg_quant_data %>%
  mutate(Genotype = factor(Genotype, levels = c('+_+', 'dv5_dv5', 'BxGal4_+', 'BxGal4_GliRNAi')))

# Print out summary of the data
str(raw_dlg_quant_data)
```

```
## tibble [100 x 10] (S3: tbl_df/tbl/data.frame)
##  $ Fly_number     : num [1:100] 1 2 3 4 5 6 7 8 9 10 ...
##  $ Genotype       : Factor w/ 4 levels "+_+","dv5_dv5",..: 1 3 2 4 1 3 2 4 1 3 ...
##  $ Cell_number    : num [1:100] 1 2 3 4 5 6 7 8 9 10 ...
##  $ Anterior       : num [1:100] 179.9 177.6 83.1 148.8 154.8 ...
##  $ Proximodistal1 : num [1:100] 88.8 75.6 75.1 135.1 98.6 ...
##  $ A/PD1          : num [1:100] 2.03 2.35 1.11 1.1 1.57 ...
##  $ Posterior      : num [1:100] 108.8 146.7 89.2 120.6 161.5 ...
##  $ Proximodistal 2: num [1:100] 76.4 71.9 79.2 98.2 92.9 ...
##  $ P/PD2          : num [1:100] 1.42 2.04 1.13 1.23 1.74 ...
##  $ Average_AP_PD  : num [1:100] 1.72 2.19 1.12 1.16 1.65 ...
```

```r
head(raw_dlg_quant_data)
```

```
## # A tibble: 6 x 10
##   Fly_number Genotype      Cell_number Anterior Proximodistal1 `A/PD1` Posterior
##        <dbl> <fct>               <dbl>    <dbl>          <dbl>   <dbl>     <dbl>
## 1          1 +_+                     1    180.            88.8    2.03      109.
## 2          2 BxGal4_+                2    178.            75.6    2.35      147.
## 3          3 dv5_dv5                 3     83.1           75.1    1.11       89.2
## 4          4 BxGal4_GliRN~           4    149.           135.     1.10      121.
## 5          5 +_+                     5    155.            98.6    1.57      162.
## 6          6 BxGal4_+                6    134.            81.6    1.64      124.
## # i 3 more variables: `Proximodistal 2` <dbl>, `P/PD2` <dbl>,
## #   Average_AP_PD <dbl>
```

```r
summary(raw_dlg_quant_data)
```

```
##    Fly_number              Genotype    Cell_number        Anterior
##  Min.   : 1.00   +_+           :25   Min.   :  1.00   Min.   : 63.48
##  1st Qu.: 5.75   dv5_dv5       :25   1st Qu.: 25.75   1st Qu.: 98.20
##  Median :10.50   BxGal4_+      :25   Median : 50.50   Median :118.72
##  Mean   :10.50   BxGal4_GliRNAi:25   Mean   : 50.50   Mean   :120.49
##  3rd Qu.:15.25                       3rd Qu.: 75.25   3rd Qu.:140.95
##  Max.   :20.00                       Max.   :100.00   Max.   :196.37
##  Proximodistal1       A/PD1          Posterior      Proximodistal 2
##  Min.   : 33.20   Min.   :0.6531   Min.   : 66.46   Min.   : 44.11
##  1st Qu.: 62.14   1st Qu.:1.2320   1st Qu.: 94.37   1st Qu.: 62.10
##  Median : 84.33   Median :1.4726   Median :115.73   Median : 76.82
##  Mean   : 82.53   Mean   :1.5485   Mean   :117.65   Mean   : 81.80
##  3rd Qu.: 98.25   3rd Qu.:1.8299   3rd Qu.:137.27   3rd Qu.: 98.66
##  Max.   :138.39   Max.   :2.9894   Max.   :198.92   Max.   :147.60
##      P/PD2        Average_AP_PD
##  Min.   :0.6294   Min.   :0.7289
##  1st Qu.:1.1174   1st Qu.:1.1839
##  Median :1.4952   Median :1.4967
##  Mean   :1.5293   Mean   :1.5389
##  3rd Qu.:1.9117   3rd Qu.:1.8777
##  Max.   :2.6733   Max.   :2.5116
```

```r
dlg_quant_data <- raw_dlg_quant_data %>%
  group_by(Fly_number, Genotype) %>%
  summarize(score = mean(Average_AP_PD))
```

```
## `summarise()` has grouped output by 'Fly_number'. You can override using the
## `.groups` argument.
```

```r
# Print out summary of the data
head(dlg_quant_data)
```

```
## # A tibble: 6 x 3
## # Groups:   Fly_number [6]
##   Fly_number Genotype       score
```

```
##        <dbl> <fct>          <dbl>
## 1          1 +_+            1.98
## 2          2 BxGal4_+       2.00
## 3          3 dv5_dv5        1.14
## 4          4 BxGal4_GliRNAi 0.944
## 5          5 +_+            2.01
## 6          6 BxGal4_+       2.04
```

Notice that for the 5 randomly sampled cells from the wing of the same fly, we compute the mean of their Average AP/PD values and call that the `score` for that fly. This should give a more accurate measurement of the change in the cells for that fly.

At this point, we work with `dlg_quant_data` that contains the following columns for the analysis:

Column name	Description
`Fly_number`	Unique identifier for each fly
`Genotype`	Genotype classification
`score`	Mean of the Average AP/PD values for the 5 cells sampled from the same fly wing. This number is the single value that represents the AP/PD ratio of the "typical" cell in a wing of that fly.

Explore

This section gives some summary statistics and initial visualizations of the dataset.

Means and Standard deviations of Score

```
summary_cell_val_data <- dlg_quant_data %>%
  group_by(Genotype) %>%
  summarize(mean_cell_val = mean(score, na.rm = TRUE),
            sd_cell_val = sd(score, na.rm = TRUE)
)

summary_cell_val_data
```

```
## # A tibble: 4 x 3
##   Genotype       mean_cell_val sd_cell_val
##   <fct>                  <dbl>       <dbl>
## 1 +_+                     1.91       0.168
## 2 dv5_dv5                 1.25       0.111
## 3 BxGal4_+                1.87       0.188
## 4 BxGal4_GliRNAi          1.13       0.247
```

Plots of Score distributions

```
custom_labels <- c(
  "+_+" = TeX("+/+"),
  "dv5_dv5" = TeX("$\\textit{Gli}^{dv5}/\\textit{Gli}^{dv5}$"),
  "BxGal4_+" = TeX("\\textit{bx}-Gal4$>$\\textit{lacZ}"),
  "BxGal4_GliRNAi" = TeX("\\textit{bx}-Gal4$>$\\textit{Gli}-RNAi")
)

set.seed(2)  # Set seed for the jitter to be fixed
p <- ggplot(dlg_quant_data, aes(x = Genotype, y = score)) +
  geom_jitter(width = 0.05, alpha = 0.6) +  # jittered points
  stat_summary(fun = mean, geom = "crossbar", width=0.5, color = "black",
               position = position_dodge(width = 1.0)) +  # mean marker
  labs(title = "Scores by Genotype with Mean", x = NULL, y = "Dlg AP/DV") +
  geom_hline(yintercept = 1, linetype = "dotted", color = "black") +  # dotted line at y = 1
  scale_x_discrete(labels = custom_labels) +
  scale_y_continuous(
    breaks = seq(0.0, 2.5, by = 0.5), # Setting breaks for every unit
    labels = seq(0.0, 2.5, by = 0.5),  # Corresponding labels for the breaks
    minor_breaks = NULL,
    limits = c(0, 2.5)
  ) +
  theme_minimal() +
  theme(axis.text.x = element_text(angle = 30, hjust = 1))
# Save the plot
# ggsave("figures/dlg_scores.tiff", plot = p, width = 7, height = 3, dpi = 300)
```

```r
# ggsave("figures/dlg_scores.svg", plot = p, device="svg", width = 2.5, height = 3.1, dpi = 300)
p
```

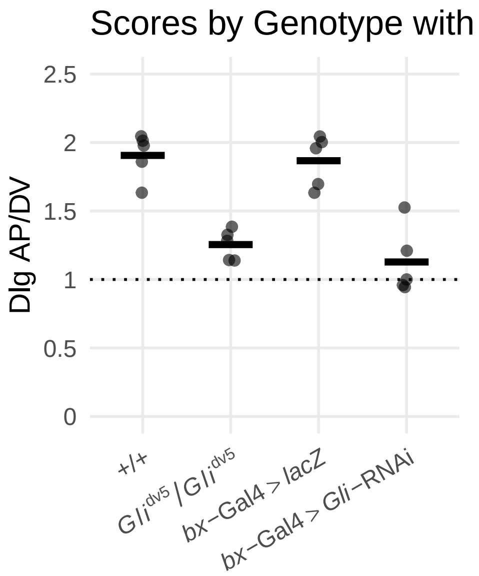

The plot above shows the distribution of the scores in each genotype category. The black horizontal line shows the mean score for that genotype. The plot above shows that there appears to be a difference in the means for each genotype's score values. We investigate if these differences are statistically significant in the next section using an ANOVA test.

Analysis

Check one-way ANOVA assumptions

We begin by first checking the ANOVA model assumptions. The plots and analyses below show that the residuals of the ANOVA model are roughly normally distributed (QQ-plot follows a straight-line pattern) and the variances of the score values for each genotype are roughly equal. Finally, since the cells were sampled randomly from different fruitflies, the samples in each genotype group are independent of the samples from the other genotype groups and mean of all cells from one fruitfly is independent from the mean of the cells from the other fruitflies within the same genotype group.

```r
anova_model <- aov(score ~ Genotype, data = dlg_quant_data)

#create Q-Q plot to compare this dataset to a theoretical normal distribution
qqnorm(anova_model$residuals)
qqline(anova_model$residuals)
```

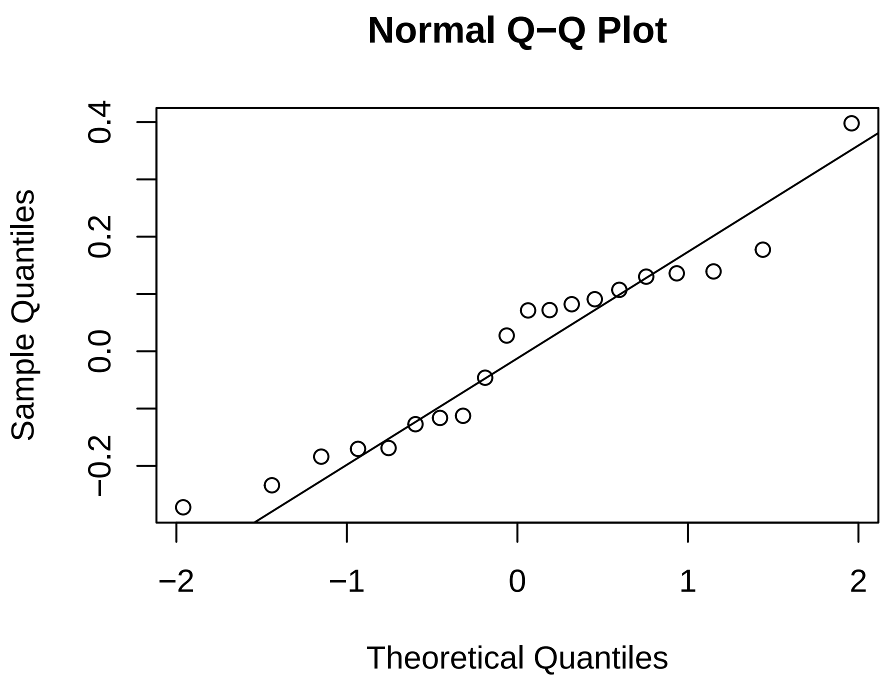


```
bartlett.test(score ~ Genotype, data = dlg_quant_data)
```

```
##
##  Bartlett test of homogeneity of variances
##
## data:  score by Genotype
## Bartlett's K-squared = 2.1995, df = 3, p-value = 0.532
```

The one-way ANOVA model analysis

```
summary(anova_model)
```

```
##             Df Sum Sq Mean Sq F value   Pr(>F)
## Genotype     3 2.4604  0.8201      24 3.65e-06 ***
## Residuals   16 0.5468  0.0342
## ---
## Signif. codes:  0 '***' 0.001 '**' 0.01 '*' 0.05 '.' 0.1 ' ' 1
```

```
post_hoc_test <- TukeyHSD(anova_model)
post_hoc_test
```

```
##   Tukey multiple comparisons of means
##     95% family-wise confidence level
##
## Fit: aov(formula = score ~ Genotype, data = dlg_quant_data)
```

```
##
## $Genotype
##                                 diff        lwr        upr     p adj
## dv5_dv5-+_+              -0.65105062 -0.9855658 -0.3165354 0.0002247
## BxGal4_+-+_+             -0.03883903 -0.3733542  0.2956761 0.9868837
## BxGal4_GliRNAi-+_+       -0.77811530 -1.1126305 -0.4436001 0.0000299
## BxGal4_+-dv5_dv5          0.61221159  0.2776964  0.9467268 0.0004281
## BxGal4_GliRNAi-dv5_dv5   -0.12706468 -0.4615799  0.2074505 0.7022959
## BxGal4_GliRNAi-BxGal4_+ -0.73927626 -1.0737914 -0.4047611 0.0000545
```

```r
# Convert Tukey result to a tidy data frame
tukey_df <- tidy(post_hoc_test)
# Plot with rotated x-axis labels
ggplot(tukey_df, aes(x = contrast, y = estimate)) +
  geom_point() +
  geom_errorbar(aes(ymin = conf.low, ymax = conf.high), width = 0.2) +
  geom_hline(yintercept = 0, linetype = "dashed") +
  theme_minimal() +
  theme(axis.text.x = element_text(angle = 45, hjust = 1)) +
  labs(title = "95% family-wise confidence level", y = "Difference in Means", x = "Comparison")
```

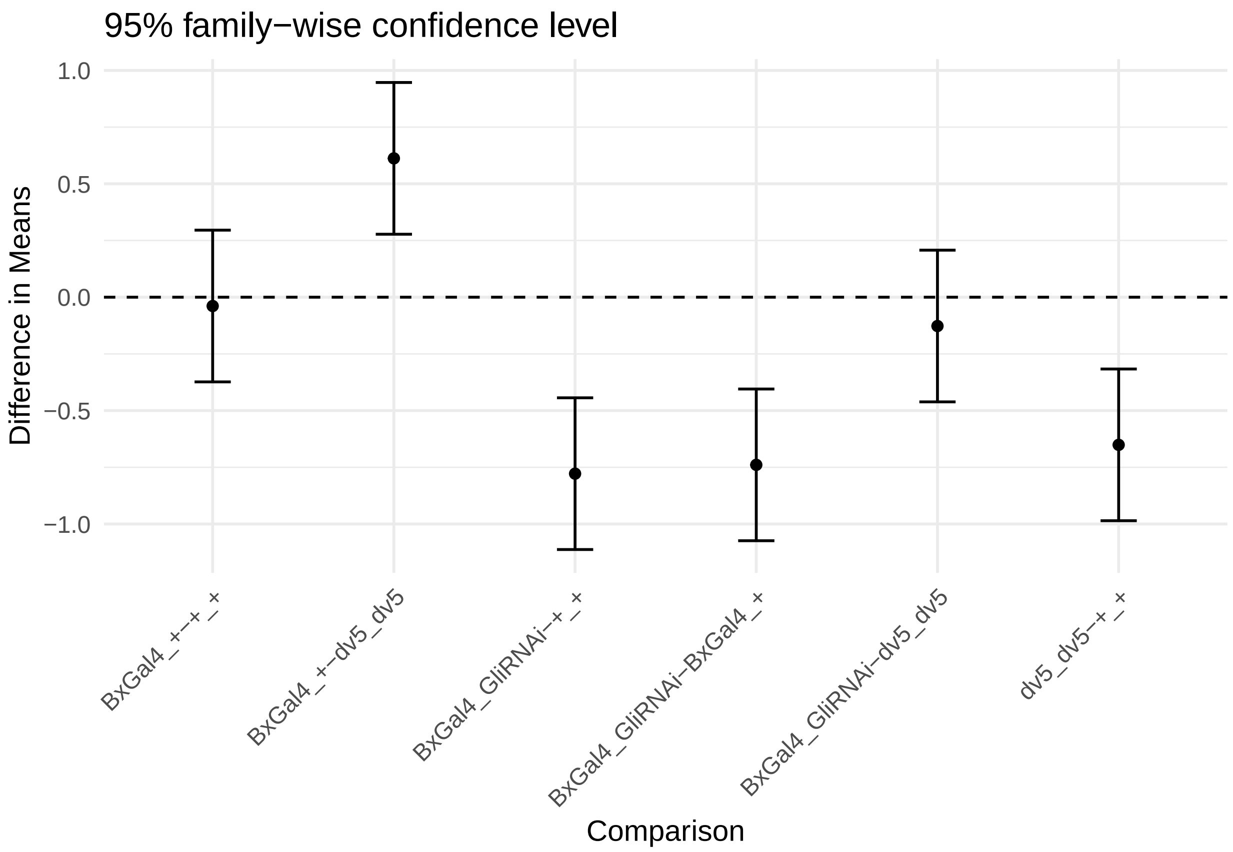

The one-way ANOVA model shows that there is a statistically significant difference between the mean of the score values across the 4 genotypes. A post-hoc analysis using Tukey's HSD reveals the pairs that show the most significant differences.

All pairings show significant differences except for the comparisons `BxGal4_+` to `+_+` ($p = 0.986$) and `dv5_dv5` to `BxGal4_GliRNAi` ($p = 0.702$).

The comparisons of interest are as follows:

- Genotype `+_+` to `dv5_dv5` shows that the score values drop by 0.65 which is statistically significant. That means the AP:PD ratio goes from being 1.90 to 1.25 when looking at cells from the `+_+` fly wings to the `dv5_dv5` fly wings.
- Genotype `BxGal4_+` to `BxGal4_GliRNAi` shows that the score values drop by 0.74 which is statistically significant. That means the AP:PD ratio goes from being 1.87 to 1.13 when looking at cells from the `BxGal4_+` fly wings to the `BxGal4_GliRNAi` fly wings.
