# Supplementary material for: A role for Myo-II zipper and spaghetti squash in Gliotactin-dependent Drosophila melanogaster wing hair planar cell polarity
Source: PLoS One. 2025 Jul 23;20(7):e0328970. doi: 10.1371/journal.pone.0328970 (PMC12286378; doi:10.1371/journal.pone.0328970)
Supplement: S7 File — A summary document of the statistical analyses used to quantify Sqh and Zip localization (as summarized in Fig 4F-G). (PDF) [file pone.0328970.s007.pdf]

Supplemental Material 7: Sqh and Zip Data Analysis Report

June 2, 2025

This file performs the statistical analyses on the pixel intensity of sqh and zip flies cells. In this document, we group by fly number to compute one `Ratio_35TOperiphery` value (termed "score") for the fly for each genotype.

Data description

The dataset columns have the following descriptions.

Column Name	Description
`Fly_number`	Unique identifier for each fly
`Type`	MRLC (i.e. Sqh) or ZIP
`Genotype`	Genotype classification
`Cell_number`	Cell number sampled
`35by35_area`	Area (in pixels) of the 35x35 pixel region centered on the cell
`35by35_mean`	Mean pixel intensity within the 35x35 region
`45by45_area`	Area (in pixels) of the 45x45 pixel region centered on the cell
`45by45_mean`	Mean pixel intensity within the 45x45 region
`35A*M`	Product of area and mean intensity for the 35x35 region
`45A*M`	Product of area and mean intensity for the 45x45 region
`periphery_mean`	Mean pixel intensity in the peripheral region around the cell
`Ratio_35TOperiphery`	Ratio of the 35by35_mean to the periphery_mean, indicating relative intensity

The key columns are the `Genotype` and the `Ratio_35TOperiphery`. The `Ratio_35TOperiphery` is a single value for each cell that signifies the ratio of the pixel intensity of the inside of the cell to it's periphery.

Genotype description

Genotype	Description
MRLC_+_+	Sqh-GFP/+
MRLC_dv5_dv5	Gli^{dv5}/Gli^{dv5}; Sqh-GFP/+
MRLC_BxGal4_+	Bx-Gal4/+; UAS-lacZ/+
MRLC_BxGal4_GliRNAi	Bx-Gal4/+; UAS-Gli-RNAi/+
ZIPgfp_+_+	Zip-GFP/+
ZIPgfp_dv5_dv5	Zip-GFP, Gli^{dv5}/Gli^{dv5}

Load necessary libraries and do setup

```r
library(tidyverse)
```

```
## Warning: package 'dplyr' was built under R version 4.3.3

## -- Attaching core tidyverse packages ------------------------ tidyverse 2.0.0 --
## v dplyr     1.1.4     v readr     2.1.4
## v forcats   1.0.0     v stringr   1.5.0
## v ggplot2   3.5.2     v tibble    3.2.1
## v lubridate 1.9.3     v tidyr     1.3.0
## v purrr     1.0.2
## -- Conflicts ------------------------------------------ tidyverse_conflicts() --
## x dplyr::filter() masks stats::filter()
## x dplyr::lag()    masks stats::lag()
## i Use the conflicted package (<http://conflicted.r-lib.org/>) to force all conflicts to become error
```

```r
library(readxl)
library(ggplot2)
library(broom)
library(latex2exp)
```

```
## Warning: package 'latex2exp' was built under R version 4.3.3
```

Get data ready

Load and clean data

```r
raw_sqh_zip_quant_data <- read_excel("data/sqh_zip_quantification.xlsx")

# Reorder and set Genotype factor
raw_sqh_zip_quant_data <- raw_sqh_zip_quant_data %>%
mutate(Genotype = factor(Genotype, levels = c('MRLC_+_+', 'MRLC_dv5_dv5',
                                              'MRLC_BxGal4_+', 'MRLC_BxGal4_GliRNAi',
                                              'ZIPgfp_+_+', 'ZIPgfp_dv5_dv5')))

# Print out summary of the data
str(raw_sqh_zip_quant_data)
```

```
## tibble [150 x 12] (S3: tbl_df/tbl/data.frame)
##  $ Fly_number        : num [1:150] 1 1 1 1 1 2 2 2 2 2 ...
##  $ Type              : chr [1:150] "MRLC" "MRLC" "MRLC" "MRLC" ...
##  $ Genotype          : Factor w/ 6 levels "MRLC_+_+","MRLC_dv5_dv5",..: 1 1 1 1 1 3 3 3 3 3 ...
##  $ Cell_number       : num [1:150] 1 2 3 4 5 6 7 8 9 10 ...
##  $ 35by35_area       : num [1:150] 973 973 973 973 973 973 973 973 973 973 ...
##  $ 35by35_mean       : num [1:150] 67.3 49.3 56.1 60.6 54.1 ...
##  $ 45by45_area       : num [1:150] 1597 1597 1597 1597 1597 ...
##  $ 45by45_mean       : num [1:150] 61.3 46.2 53.7 57.6 51.6 ...
```

```
##  $ 35A*M              : num [1:150] 65515 47973 54624 58962 52596 ...
##  $ 45A*M              : num [1:150] 97966 73722 85792 91997 82348 ...
##  $ periphery_mean     : num [1:150] 52 41.3 49.9 52.9 47.7 ...
##  $ Ratio_35TOperiphery: num [1:150] 1.29 1.19 1.12 1.14 1.13 ...
```

```r
head(raw_sqh_zip_quant_data)
```

```
## # A tibble: 6 x 12
##   Fly_number Type  Genotype      Cell_number `35by35_area` `35by35_mean`
##        <dbl> <chr> <fct>               <dbl>         <dbl>         <dbl>
## 1          1 MRLC  MRLC_+_+                1           973          67.3
## 2          1 MRLC  MRLC_+_+                2           973          49.3
## 3          1 MRLC  MRLC_+_+                3           973          56.1
## 4          1 MRLC  MRLC_+_+                4           973          60.6
## 5          1 MRLC  MRLC_+_+                5           973          54.1
## 6          2 MRLC  MRLC_BxGal4_+           6           973          69.2
## # i 6 more variables: `45by45_area` <dbl>, `45by45_mean` <dbl>, `35A*M` <dbl>,
## #   `45A*M` <dbl>, periphery_mean <dbl>, Ratio_35TOperiphery <dbl>
```

```r
summary(raw_sqh_zip_quant_data)
```

```
##    Fly_number       Type                              Genotype   Cell_number
##  Min.   : 1.0   Length:150         MRLC_+_+             :25   Min.   :  1.00
##  1st Qu.: 8.0   Class :character   MRLC_dv5_dv5         :25   1st Qu.: 38.25
##  Median :15.5   Mode  :character   MRLC_BxGal4_+        :25   Median : 75.50
##  Mean   :15.5                      MRLC_BxGal4_GliRNAi:25   Mean   : 75.50
##  3rd Qu.:23.0                      ZIPgfp_+_+           :25   3rd Qu.:112.75
##  Max.   :30.0                      ZIPgfp_dv5_dv5       :25   Max.   :150.00
##   35by35_area   35by35_mean      45by45_area    45by45_mean
##  Min.   :973   Min.   : 13.90   Min.   :1597   Min.   : 12.99
##  1st Qu.:973   1st Qu.: 28.02   1st Qu.:1597   1st Qu.: 26.03
##  Median :973   Median : 42.96   Median :1597   Median : 41.59
##  Mean   :973   Mean   : 43.18   Mean   :1597   Mean   : 41.29
##  3rd Qu.:973   3rd Qu.: 51.83   3rd Qu.:1597   3rd Qu.: 49.93
##  Max.   :973   Max.   :133.76   Max.   :1597   Max.   :132.61
##      35A*M            45A*M         periphery_mean     Ratio_35TOperiphery
##  Min.   : 13526   Min.   : 20748   Min.   :  8.386   Min.   :0.7489
##  1st Qu.: 27263   1st Qu.: 41575   1st Qu.: 23.780   1st Qu.:1.0128
##  Median : 41802   Median : 66422   Median : 38.146   Median :1.1501
##  Mean   : 42019   Mean   : 65942   Mean   : 38.338   Mean   :1.1782
##  3rd Qu.: 50429   3rd Qu.: 79739   3rd Qu.: 47.186   3rd Qu.:1.2855
##  Max.   :130146   Max.   :211777   Max.   :130.819   Max.   :2.0487
```

Filter to just MRLC (sqh)

```r
sqh_quant_data <- raw_sqh_zip_quant_data %>%
  filter(Type == "MRLC") %>%
  mutate(Genotype = droplevels(Genotype)) %>%
  group_by(Fly_number, Genotype) %>%
  summarize(score = mean(Ratio_35TOperiphery))
```

```
## `summarise()` has grouped output by 'Fly_number'. You can override using the
## `.groups` argument.
```

```r
# Print out summary of the data
head(sqh_quant_data)
```

```
## # A tibble: 6 x 3
## # Groups:   Fly_number [6]
##   Fly_number Genotype              score
##        <dbl> <fct>                 <dbl>
## 1          1 MRLC_+_+              1.18
## 2          2 MRLC_BxGal4_+         1.25
## 3          3 MRLC_dv5_dv5          0.991
## 4          4 MRLC_BxGal4_GliRNAi   1.02
## 5          7 MRLC_+_+              1.15
## 6          8 MRLC_BxGal4_+         1.26
```

Filter to just ZIP (zip)

```r
zip_quant_data <- raw_sqh_zip_quant_data %>%
  filter(Type == "ZIP") %>%
  mutate(Genotype = droplevels(Genotype)) %>%
  group_by(Fly_number, Genotype) %>%
  summarize(score = mean(Ratio_35TOperiphery))
```

```
## `summarise()` has grouped output by 'Fly_number'. You can override using the
## `.groups` argument.
```

```r
# Print out summary of the data
str(zip_quant_data)
```

```
## gropd_df [10 x 3] (S3: grouped_df/tbl_df/tbl/data.frame)
##  $ Fly_number: num [1:10] 5 6 11 12 17 18 23 24 29 30
##  $ Genotype  : Factor w/ 2 levels "ZIPgfp_+_+","ZIPgfp_dv5_dv5": 1 2 1 2 1 2 1 2 1 2
##  $ score     : num [1:10] 1.73 1.39 1.36 0.96 1.62 ...
##  - attr(*, "groups")= tibble [10 x 2] (S3: tbl_df/tbl/data.frame)
##   ..$ Fly_number: num [1:10] 5 6 11 12 17 18 23 24 29 30
##   ..$ .rows     : list<int> [1:10]
##   .. ..$ : int 1
##   .. ..$ : int 2
##   .. ..$ : int 3
##   .. ..$ : int 4
##   .. ..$ : int 5
##   .. ..$ : int 6
##   .. ..$ : int 7
##   .. ..$ : int 8
##   .. ..$ : int 9
##   .. ..$ : int 10
##   .. ..@ ptype: int(0)
##   ..- attr(*, ".drop")= logi TRUE
```

```r
head(zip_quant_data)
```

```
## # A tibble: 6 x 3
## # Groups:   Fly_number [6]
##   Fly_number Genotype       score
##        <dbl> <fct>          <dbl>
## 1          5 ZIPgfp_+_+     1.73
## 2          6 ZIPgfp_dv5_dv5 1.39
## 3         11 ZIPgfp_+_+     1.36
## 4         12 ZIPgfp_dv5_dv5 0.960
## 5         17 ZIPgfp_+_+     1.62
## 6         18 ZIPgfp_dv5_dv5 1.21
```

```r
summary(zip_quant_data)
```

```
##    Fly_number              Genotype     score
##  Min.   : 5.00   ZIPgfp_+_+    :5   Min.   :0.9602
##  1st Qu.:11.25   ZIPgfp_dv5_dv5:5   1st Qu.:1.1370
##  Median :17.50                      Median :1.2705
##  Mean   :17.50                      Mean   :1.2881
##  3rd Qu.:23.75                      3rd Qu.:1.3842
##  Max.   :30.00                      Max.   :1.7253
```

Notice that for the 5 randomly sampled cells from the wing of the same fly, we compute the mean of their `Ratio_35TOperiphery` values and call that the `score` for that fly. This should give a more accurate measurement of the change in the cells for that fly.

At this point, we work with `BLANK_quant_data` (where `BLANK` is either `sqh` or `zip`) that contains the following columns for the analysis:

Column name	Description
`Fly_number`	Unique identifier for each fly
`Genotype`	Genotype classification
`score`	Mean of the `Ratio_35TOperiphery` values for the 5 cells sampled from the same fly wing. This number is the single value that represents the `Ratio_35TOperiphery` of the “typical” cell in a wing of that fly.

MRLC (sqh) Analysis

This section just shows the MRLC analysis.

Explore

This subsection gives some summary statistics and initial visualizations of the dataset.

Means and Standard deviations of Score

```
summary_cell_val_data <- sqh_quant_data %>%
  group_by(Genotype) %>%
  summarize(mean_cell_val = mean(score, na.rm = TRUE),
            sd_cell_val = sd(score, na.rm = TRUE)
)

summary_cell_val_data
```

```
## # A tibble: 4 x 3
##   Genotype            mean_cell_val sd_cell_val
##   <fct>                       <dbl>       <dbl>
## 1 MRLC_+_+                     1.20      0.0546
## 2 MRLC_dv5_dv5                 1.00      0.0618
## 3 MRLC_BxGal4_+                1.26      0.0360
## 4 MRLC_BxGal4_GliRNAi          1.02      0.0164
```

Plots of Score distributions

```
# Custom labels
custom_labels <- c(
  "MRLC_+_+" = TeX("+/+"),
  "MRLC_dv5_dv5" = TeX("$\\textit{Gli}^{dv5}/\\textit{Gli}^{dv5}"),
  "MRLC_BxGal4_+" = TeX("\\textit{bx}-Gal4$>$\\textit{lacZ}"),
  "MRLC_BxGal4_GliRNAi" = TeX("\\textit{bx}-Gal4$>$\\textit{Gli}-RNAi")
)

# Set seed for reproducibility
set.seed(2)

# Plot
p <- ggplot(sqh_quant_data, aes(x = Genotype, y = score)) +
  geom_jitter(width = 0.05, alpha = 0.6) +  # jittered points
  stat_summary(fun = mean, geom = "crossbar", width = 0.5, color = "black",
               position = position_dodge(width = 1.0)) +  # mean marker
  labs(title = "Sqh Scores by Genotype", x = NULL, y = "Sqh center/periphery") +
  geom_hline(yintercept = 1, linetype = "dotted", color = "black") +  # dotted line at y = 1
  scale_x_discrete(labels = custom_labels) +
  scale_y_continuous(
    breaks = seq(0.0, 1.5, by = 0.5), # Setting breaks for every unit
```

```
    labels = seq(0.0, 1.5, by = 0.5),  # Corresponding labels for the breaks
    minor_breaks = NULL,
    limits = c(0.0, 1.5)
  ) +
  theme_minimal() +
  theme(axis.text.x = element_text(angle = 30, hjust = 1))

# Optional: Save the plot
# ggsave("figures/sqh_scores.tiff", plot = p, width = 7, height = 3, dpi = 300)
# ggsave("figures/sqh_scores.svg", plot = p, device="svg", width = 2.5, height = 3.1, dpi = 300)

p
```

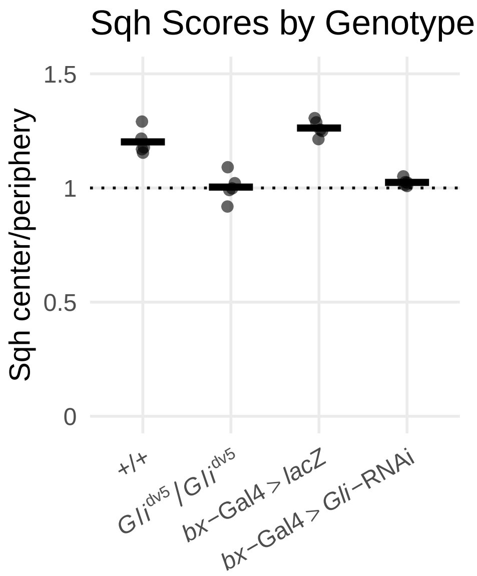

The plot above shows the distribution of the scores in each genotype category. The black points show the actual values and X shows the mean score for that genotype. The plot above shows that there appears to be a difference in the means for each genotype's score values. We investigate if these differences are statistically significant in the next section using an ANOVA test.

Analysis

Check one-way ANOVA assumptions

We begin by first checking the ANOVA model assumptions. The plots and analyses below show that the residuals of the ANOVA model are roughly normally distributed (QQ-plot follows a straight-line pattern) and the variances of the score values for each genotype are roughly equal. Finally, since the cells were sampled randomly from different fruitflies, the samples in each genotype group are independent of the samples from the other genotype groups and mean of all cells from one fruitfly is independent from the mean of the cells from the other fruitflies within the same genotype group.

```
anova_model <- aov(score ~ Genotype, data = sqh_quant_data)

#create Q-Q plot to compare this dataset to a theoretical normal distribution
```

```r
qqnorm(anova_model$residuals)
qqline(anova_model$residuals)
```

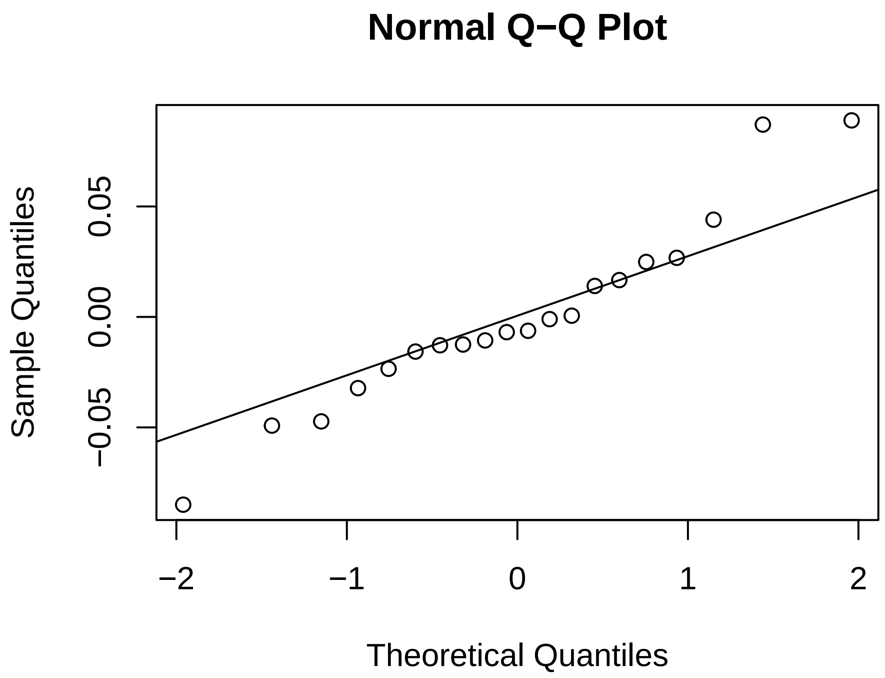


```r
bartlett.test(score ~ Genotype, data = sqh_quant_data)
```

```
##
##  Bartlett test of homogeneity of variances
##
## data:  score by Genotype
## Bartlett's K-squared = 5.6955, df = 3, p-value = 0.1274
```

The one-way ANOVA model analysis

```r
summary(anova_model)
```

```
##             Df  Sum Sq Mean Sq F value   Pr(>F)
## Genotype     3 0.24808 0.08269   39.53 1.26e-07 ***
## Residuals   16 0.03347 0.00209
## ---
## Signif. codes:  0 '***' 0.001 '**' 0.01 '*' 0.05 '.' 0.1 ' ' 1
```

```r
post_hoc_test <- TukeyHSD(anova_model)
post_hoc_test
```

```
##   Tukey multiple comparisons of means
##     95% family-wise confidence level
##
## Fit: aov(formula = score ~ Genotype, data = sqh_quant_data)
##
## $Genotype
##                                           diff         lwr         upr     p adj
## MRLC_dv5_dv5-MRLC_+_+             -0.19789628 -0.28065612 -0.11513643 0.0000215
## MRLC_BxGal4_+-MRLC_+_+             0.06081786 -0.02194198  0.14357771 0.1943225
## MRLC_BxGal4_GliRNAi-MRLC_+_+      -0.17744081 -0.26020066 -0.09468096 0.0000772
## MRLC_BxGal4_+-MRLC_dv5_dv5         0.25871414  0.17595429  0.34147398 0.0000007
## MRLC_BxGal4_GliRNAi-MRLC_dv5_dv5   0.02045547 -0.06230438  0.10321531 0.8927902
## MRLC_BxGal4_GliRNAi-MRLC_BxGal4_+ -0.23825867 -0.32101852 -0.15549882 0.0000021
```

```r
# Convert Tukey result to a tidy data frame
tukey_df <- tidy(post_hoc_test)
# Plot with rotated x-axis labels
ggplot(tukey_df, aes(x = contrast, y = estimate)) +
  geom_point() +
  geom_errorbar(aes(ymin = conf.low, ymax = conf.high), width = 0.2) +
  geom_hline(yintercept = 0, linetype = "dashed") +
  theme_minimal() +
  theme(axis.text.x = element_text(angle = 45, hjust = 1)) +
  labs(title = "95% family-wise confidence level", y = "Difference in Means", x = "Comparison")
```

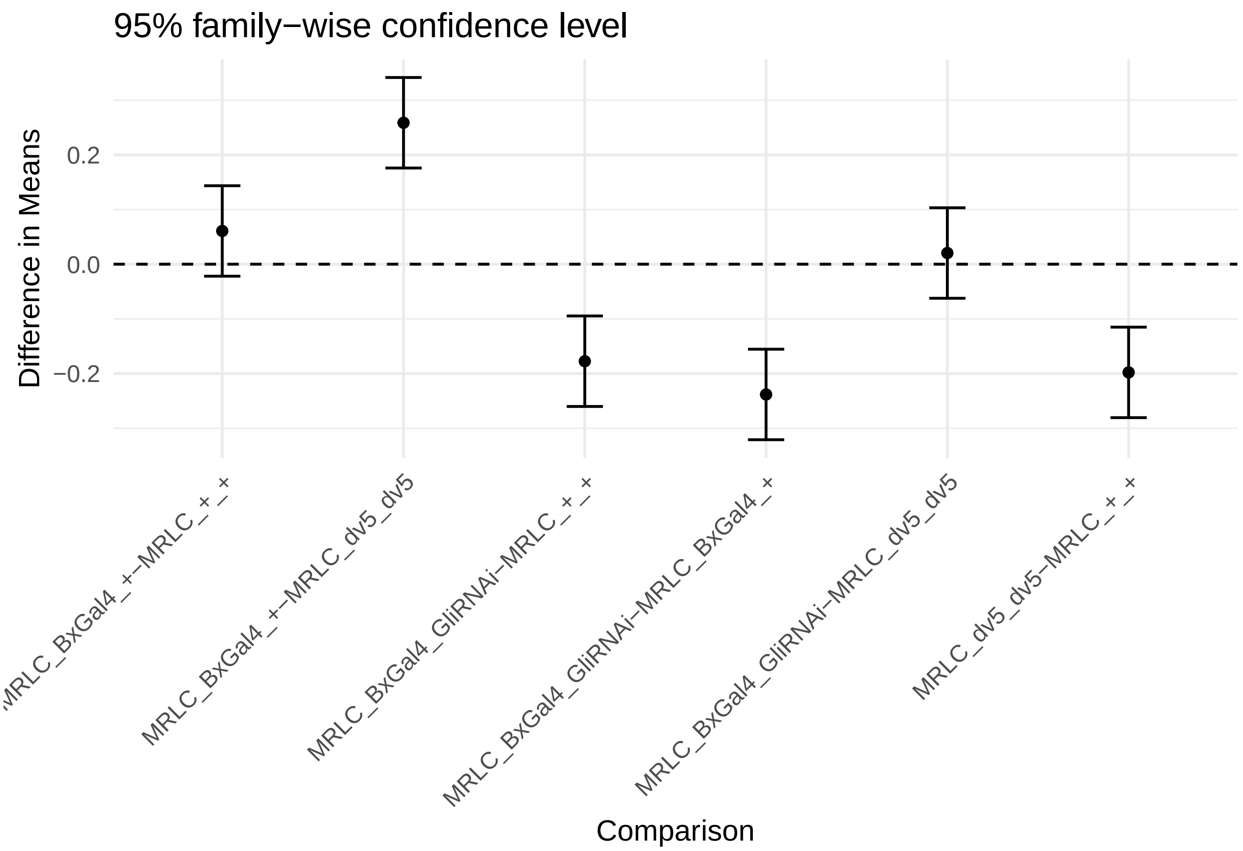

The one-way ANOVA model shows that there is a statistically significant difference between the mean of the

score values across the 4 genotypes. A post-hoc analysis using Tukey's HSD reveals the pairs that show the most significant differences.

All pairings show significant differences except for the comparisons `MRLC_BxGal4_+` to `MRLC_+_+` ($p = 0.194$) and `MRLC_BxGal4_GliRNAi` to `MRLC_dv5_dv5` ($p = 0.893$).

The comparisons of interest are as follows:

- Genotype `MRLC_dv5_dv5` to `MRLC_+_+` shows that the score values drop by 0.198 which is statistically significant.
- Genotype `MRLC_BxGal4_GliRNAi` to `MRLC_BxGal4_+` shows that the score values drop by 0.238 which is statistically significant.

ZIP (zip) Analysis

This section just shows the ZIP analysis.

Explore

This subsection gives some summary statistics and initial visualizations of the dataset.

Means and Standard deviations of Score

```
summary_cell_val_data <- zip_quant_data %>%
  group_by(Genotype) %>%
  summarize(mean_cell_val = mean(score, na.rm = TRUE),
            sd_cell_val = sd(score, na.rm = TRUE)
)

summary_cell_val_data
```

```
## # A tibble: 2 x 3
##   Genotype       mean_cell_val sd_cell_val
##   <fct>                  <dbl>       <dbl>
## 1 ZIPgfp_+_+              1.45       0.217
## 2 ZIPgfp_dv5_dv5          1.13       0.181
```

Plots of Score distributions

```
# Custom labels for ZIP data
custom_labels <- c(
  "ZIPgfp_+_+" = TeX("+/+"),
  "ZIPgfp_dv5_dv5" = TeX("$\\textit{Gli}^{dv5}/\\textit{Gli}^{dv5}$")
)

# Set seed for reproducibility
set.seed(2)

# Plot
p <- ggplot(zip_quant_data, aes(x = Genotype, y = score)) +
  geom_jitter(width = 0.05, alpha = 0.6) +  # jittered points
  stat_summary(fun = mean, geom = "crossbar", width = 0.5, color = "black",
               position = position_dodge(width = 1.0)) +  # mean marker
  labs(title = "Zip Scores by Genotype", x = NULL, y = "Zip center/periphery") +
  geom_hline(yintercept = 1, linetype = "dotted", color = "black") +  # dotted line at y = 1
  scale_x_discrete(labels = custom_labels) +
  scale_y_continuous(
    breaks = seq(0.0, 2, by = 0.5), # Setting breaks for every unit
    labels = seq(0.0, 2, by = 0.5),  # Corresponding labels for the breaks
    minor_breaks = NULL,
    limits = c(0.0, 2)
  ) +
```

```
  theme_minimal() +
  theme(axis.text.x = element_text(angle = 30, hjust = 1))

# Optional: Save the plot
# ggsave("figures/zip_scores.tiff", plot = p, width = 7, height = 3, dpi = 300)
# ggsave("figures/zip_scores.svg", plot = p, device="svg", width = 2, height = 3.1, dpi = 300)

p
```

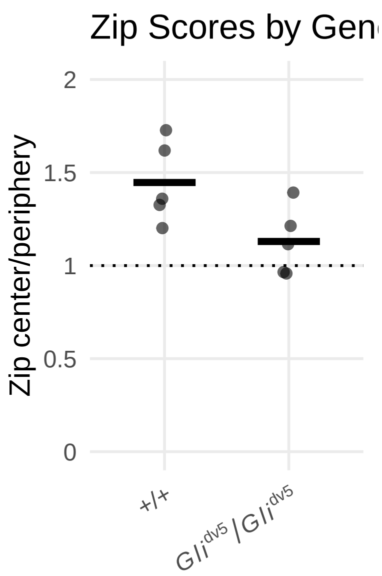

The plot above shows the distribution of the scores in each genotype category. The black points show the actual values and X shows the mean score for that genotype. The plot above shows that there appears to be a difference in the means for each genotype's score values. We investigate if these differences are statistically significant in the next section using an ANOVA test.

Analysis

Check Welch's t-test assumptions

We begin by first checking Welch's t-test assumptions. The plots and analyses below show that the variances of the score values for each genotype are roughly equal. Finally, since the cells were sampled randomly from different fruitflies, the samples in each genotype group are independent of the samples from the other genotype groups and mean of all cells from one fruitfly is independent from the mean of the cells from the other fruitflies within the same genotype group.

```
ttest_model <- t.test(score ~ Genotype, data = zip_quant_data)

bartlett.test(score ~ Genotype, data = zip_quant_data)
```

```
##
##  Bartlett test of homogeneity of variances
##
## data:  score by Genotype
## Bartlett's K-squared = 0.11228, df = 1, p-value = 0.7376
```

The t-test model analysis

```
ttest_model
```

```
##
##  Welch Two Sample t-test
##
## data:  score by Genotype
## t = 2.5055, df = 7.7588, p-value = 0.0375
## alternative hypothesis: true difference in means between group ZIPgfp_+_+ and group ZIPgfp_dv5_dv5 is
## 95 percent confidence interval:
##  0.02363927 0.61008659
## sample estimates:
##     mean in group ZIPgfp_+_+ mean in group ZIPgfp_dv5_dv5
##                     1.446577                     1.129714
```

The t-test shows that there is a statistically significant difference (at the 0.05 level of significance) between the mean of the score values across the 2 genotypes for the ZIP.
